# Live cell imaging reveals 3′-UTR dependent mRNA sorting to synapses

**DOI:** 10.1038/s41467-019-11123-x

**Published:** 2019-07-18

**Authors:** Karl E. Bauer, Inmaculada Segura, Imre Gaspar, Volker Scheuss, Christin Illig, Georg Ammer, Saskia Hutten, Eugénia Basyuk, Sandra M. Fernández-Moya, Janina Ehses, Edouard Bertrand, Michael A. Kiebler

**Affiliations:** 1 BioMedical Center, Medical Faculty, Ludwig Maximilians University, Großhaderner Str. 9, 82152 Planegg-Martinsried, Germany; 2 EMBL, Meyerhofstraße 1, 69117 Heidelberg, Germany; 3 Institut de Génétique Moléculaire de Montpellier, CNRS UMR5535, Université de Montpellier, 1919 route de Mende, 34293 Montpellier, France; 4 Present Address: Institute of Molecular Biotechnology, Dr. Bohr-Gasse 3, 1030 Vienna, Austria; 5 Present Address: MPI of Neurobiology, Am Klopferspitz 18, 82152 Martinsried, Germany; 6 Present Address: Institut de Génétique Humaine de Montpellier, CNRS UMR9002, Université de Montpellier, 141 rue de la Cardonille, 34396 Montpellier, France

**Keywords:** Fluorescence imaging, Intracellular movement, Cell polarity, RNA transport, Synaptic plasticity

## Abstract

mRNA transport restricts translation to specific subcellular locations, which is the basis for many cellular functions. However, the precise process of mRNA sorting to synapses in neurons remains elusive. Here we use *Rgs4* mRNA to investigate 3′-UTR-dependent transport by MS2 live-cell imaging. The majority of observed RNA granules display 3′-UTR independent bidirectional transport in dendrites. Importantly, the *Rgs4* 3′-UTR causes an anterograde transport bias, which requires the Staufen2 protein. Moreover, the 3′-UTR mediates dynamic, sustained mRNA recruitment to synapses. Visualization at high temporal resolution enables us to show mRNA patrolling dendrites, allowing transient interaction with multiple synapses, in agreement with the sushi-belt model. Modulation of neuronal activity by either chemical silencing or local glutamate uncaging regulates both the 3′-UTR-dependent transport bias and synaptic recruitment. This dynamic and reversible mRNA recruitment to active synapses would allow translation and synaptic remodeling in a spatially and temporally adaptive manner.

## Abstract (web summary)

Asymmetric subcellular mRNA distribution is important for local translation of neuronal mRNAs. Here the authors employed MS2 live-cell imaging and showed that the reporter mRNA containing the 3’ UTR of Rgs4 shows an anterograde transport bias, dependent on neuronal activity and the protein Staufen2, and mediates sustained mRNA recruitment to synapses.

## Introduction

Messenger RNAs (mRNAs) display a variety of subcellular localization patterns in a plethora of model systems^1–3^, including the dendritic compartment of the hippocampus^4^. Several distinct mechanisms have been proposed to explain how the sorting of specific mRNAs to subcellular locations can be achieved^2,3,5^, from simple diffusion to the more complex sushi-belt model of dendritic mRNA trafficking^6^. The latter proposes that mRNA granules patrol dendrites in a highly dynamic multidirectional fashion, without being irreversibly anchored at a single specific location. Multiple approaches demonstrated that specific transcripts can be actively transported along cytoskeletal structures^7–9^. Such active and directed transport has been hypothesized to be the driving force that mediates mRNA sorting to specific distal locations in neurons, such as postsynaptic sites or axonal growth cones, where it may become available for local translation^10–15^. This allows the tightly regulated production of the resulting protein, both spatially and temporally. Localization of mRNA and subsequent local translation are particularly important in neurons, where synapses containing a specific proteome can be located at distal dendrites far from the site of transcription. Ultimately, local protein synthesis at synapses is fundamental for learning and the formation of long-term memory^3,6,16,17^.

Previous studies investigated the role of neuronal stimulation on these processes and reported the activity-induced unpacking of mRNAs, allowing local translation in dendrites of primary hippocampal neurons^12,18–20^. In addition, glutamate uncaging induces *ß-actin* mRNA recruitment in dendrites, where it is eventually translated and the newly produced actin participates in dendritic spine remodeling^13^. However, we are only beginning to understand how mRNA is sorted to synapses.

Sorting signals, usually within the 3′-untranslated region (3′-UTR) of mRNA, play a crucial role in mRNA localization^2,21–23^. Such signals are able to interact with specific RNA binding proteins (RBPs), such as ZBP1, FMRP, or Staufen2 (Stau2), to form neuronal RNA granules^15,24,25^. Thereby, Stau2 and ZBP1 regulate the dendritic localization of *Calm3* and *ß-actin* mRNA, respectively^26,27^. Through these processes RBPs significantly contribute to synaptic function^15,28,29^.

The *negative regulator of G protein signaling 4* (*Rgs4*) mRNA is a physiological target mRNA of Stau2 previously identified in the brain^30^. It encodes a GTPase activating protein of the G protein-coupled receptor (GPCR) pathway, and therefore modulates receptor mediated neuronal signaling at the synapse^31–33^. Fluorescent in situ hybridization (FISH) has shown that *Rgs4* mRNA is present in cytosolic RNA granules localized in distal dendrites. Further analysis confirmed the presence of *Rgs4* mRNA in Stau2 granules^30^. As the long *Rgs4* 3′-UTR contains in vivo cross-linking sites for Stau2^27^, it might provide key binding sites for a direct interaction with Stau2. Silencing of Stau2 induces a reduction of endogenous *Rgs4* mRNA both in vitro and in vivo, suggesting an involvement of Stau2 in the regulation of *Rgs4* mRNA levels^30,34^.

To evaluate the role of the 3′-UTR in mediating proper subcellular sorting in mature neurons, we use *Rgs4* as a model, and generate an mRNA reporter combining the *Rgs4* 3′-UTR with an improved MS2 RNA live-cell imaging system^35,36^. This reporter system allows us to perform long-term mRNA tracking in dendrites, to (i) unravel the underlying mRNA transport dynamics mediated by a specific 3′-UTR and investigate (ii) neuronal activity and (iii) Stau2 dependency. Together, our results support a model of active, directed mRNA trafficking in a sushi-belt like fashion, promoting synaptic recruitment of mRNA, which would lead to activated translation. This in turn may trigger synaptic remodeling, which ultimately impacts neuronal function.

## Results

### *MS2 Rgs4* 3′-UTR reporter mRNA localizes to distal dendrites

**Fig. 1:**
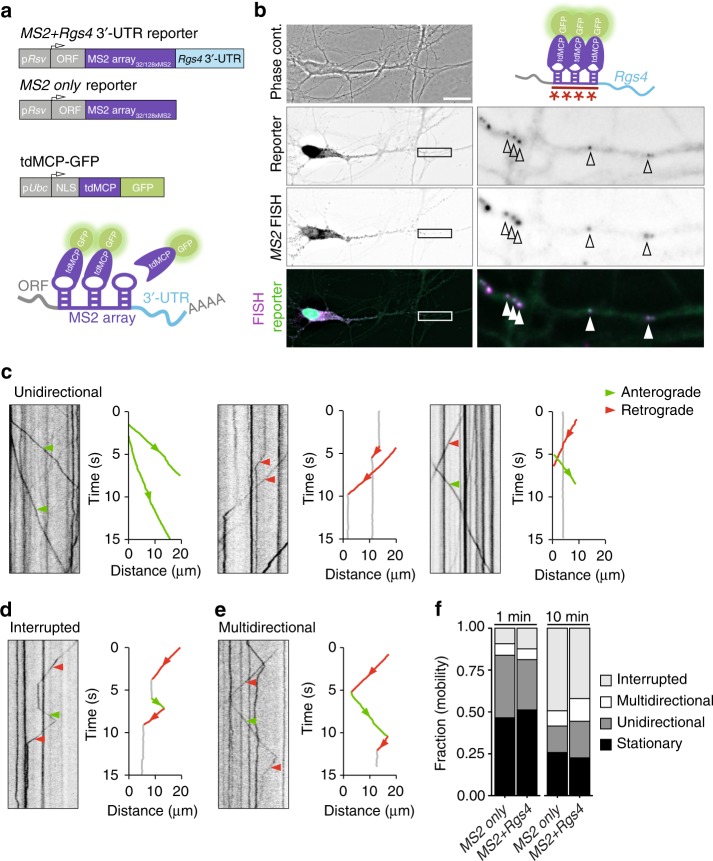
Reporter mRNAs display directed dendritic transport in hippocampal neurons. **a** Scheme of both *MS2 only* and *MS2+Rgs4* reporter constructs and tdMCP-GFP expression cassettes (upper) and the MS2 system (lower). pRSV Rous sarcoma virus promoter, pUBC Ubiquitin C promoter, ORF open reading frame, NLS nuclear localization signal, tdMCP tandem MS2 coat protein, UTR untranslated region. **b** Phase contrast, GFP fluorescence (reporter), MS2 single molecule FISH and overlay in a rat hippocampal neuron expressing both tdMCP-GFP and *MS2+Rgs4* MS2 reporter mRNA (scheme). Arrowheads indicate overlapping tdMCP-GFP bound MS2 reporter mRNA and MS2 smFISH. Fluorescent images were deconvolved to assess overlap (for unprocessed images see Supplementary Fig. 1e). Scale bar 20 µm. Boxed region is magnified in right panels. Representative kymographs (left) and extracted tracks (right) illustrating differences in unidirectional *MS2+Rgs4* 3′-UTR mRNA granule transport speed, displacement and directionality (**c**), as well as interrupted (**d**) and multidirectional transport (**e**). Anterograde and retrograde transport are indicated in green or red arrowheads and lines, respectively. **f** Quantification of relative transport dynamics of *MS2 only* and *MS2+Rgs4* 3′-UTR reporter mRNAs in 1 and 10-min time-series acquisitions, respectively

To test whether the 3′-UTR of *Rgs4* is sufficient to mediate dendritic localization and to unravel the underlying dynamics of subcellular mRNA sorting, we employed the MS2 system^35^ in cultured rat hippocampal neurons. This system makes use of the high affinity and specificity interaction between the MS2 coat protein (MCP) and the *MS2* RNA stem-loop structure. We designed mRNA reporters containing the *LacZ* ORF, followed by a stop codon, an array of either 32 or 128 *MS2* stem-loops^36^ and the *Rgs4* 3′-UTR (Fig. 1a). A second MS2 reporter mRNA lacking the *Rgs4* 3′-UTR was generated (termed ‘*MS2 only*’ throughout). These reporter mRNAs allowed us to assess the specific contribution of the *Rgs4* 3′-UTR to dendritic mRNA transport. To visualize the reporter mRNAs in living cells, we cotransfected each reporter plasmid with an expression vector encoding a C-terminally green fluorescent protein (GFP)-tagged tandem MCP (tdMCP-GFP)^37^. A nuclear localization signal (NLS) was included to sequester excess tdMCP-GFP into the nucleus^9,35,37–39^ (Fig. 1a; Supplementary Fig. 1a). Expression levels of *MS2 only* and *MS2+Rgs4* reporter mRNAs, tested by RT-qPCR, were comparable (Supplementary Fig. 1b). Single molecule FISH (smFISH)^40^, targeting the *MS2* repeats, demonstrated that the *MS2+Rgs4* 3′-UTR reporter mRNA localized to dendrites (Supplementary Fig. 1c), resembling endogenous *Rgs4* mRNA^30^. Control reporter mRNAs with no known function in dendrites, i.e. *MS2 only* or *MS2+histone-3.3* 3′-UTR, displayed dendritic localization (Supplementary Fig. 1d). This suggests that dendritic localization is not exclusively dependent on the 3′-UTR, but that other sequences or different expression levels might contribute as well. To further validate the MS2-MCP system in neurons, we cotransfected both the *MS2+Rgs4* 3′-UTR MS2 reporter and tdMCP-GFP plasmids. GFP fluorescence was clustered in discrete cytoplasmic granules that colocalized with *MS2* smFISH signal (Fig. 1b, Supplementary Fig. 1e), confirming that we reliably detected reporter mRNAs, allowing the visualization of intracellular mRNA transport in living cells. Next, we inquired how specific subcellular sorting within dendrites might be achieved.

### *Rgs4* 3′-UTR-dependent anterograde transport bias in dendrites

**Fig. 2:**
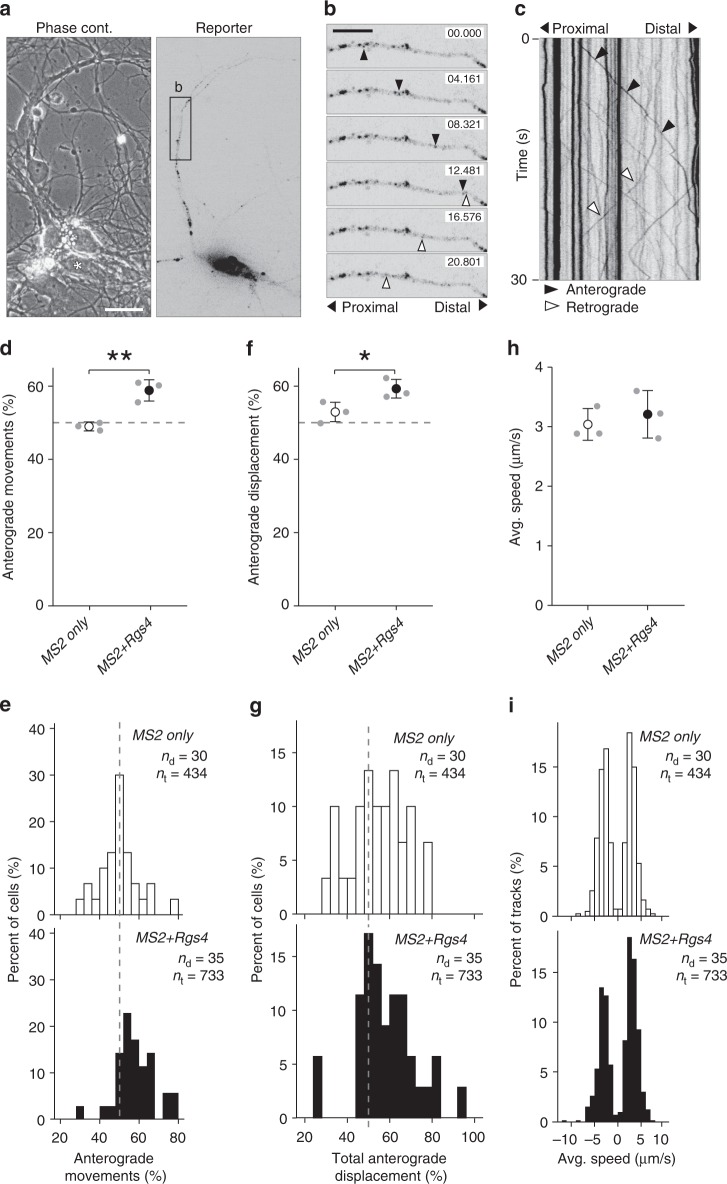
*Rgs4* 3′-UTR mediates an anterograde transport bias. **a** Representative phase contrast and GFP fluorescence of hippocampal neuronal culture cotransfected with the *MS2+Rgs4* 3′-UTR reporter and tdMCP-GFP constructs. Scale bar 20 µm. Asterisk denotes GFP positive cell. **b** Time series of the dendritic boxed region in **a**. Representative anterograde (black arrowheads) and retrograde (white arrowheads) moving mRNA granules are indicated. Scale bars 10 µm. **c** Kymograph of the dendritic region in **b**. Arrowheads indicate mRNA granules signified in **b**. Dot plots (**d**, **f**, **h**) and histograms (**e**, **g**, **i**) displaying percentage of anterograde moving granules (**d**, **e**), percentage of total anterograde displacement (**f**, **g**) and average speed (**h**, **i**) for *MS2 only* or *MS2+Rgs4* 3′-UTR reporter mRNAs, detected by tdMCP-GFP. In (**i**), positive values indicate anterograde and negative values indicate retrograde transport. Data represent mean ± standard deviation of three independent experiments (individual experiments shown as gray dots). Asterisks represent *p*-values obtained by Student’s *t* test (*p < 0.05, **p < 0.01). Data were obtained from 40-µm dendritic segments at a minimal distance of 20 µm from the cell body. At least 10 dendrites/condition/experiment were analyzed. Total number of dendrites (*n*_d_) and tracks (*n*_t_) analyzed per condition are indicated. Only displacements ≥1.5 µm were considered for analysis

To investigate dynamics underlying mRNA transport, time-lapse imaging of single neurons expressing the MS2 system was performed for 1 min at 15.3 fps (frames per second) with a spinning disk microscope. To analyze the characteristics of single trafficking RNA granules, we generated kymographs of dendritic regions at a minimal distance of 20 µm from the soma and traced single trajectories (Figs. 1c–e; 2a–c; Supplementary Movies 1–8). This revealed diverse RNA transport patterns, independent of a 3′-UTR. We observed mobile mRNA granules with differences in transport speed, displacement length and directionality (Fig. 1c, Supplementary Movies 1–3). Furthermore, two additional distinct types of mRNA granule mobility were detected. We found that mRNA granules may interrupt their movement before reinitiating transport (Fig. 1d, Supplementary Movie 4, interrupted) or may display multiple changes in direction without interrupting transport (Fig. 1e, Supplementary Movies 5 and 6, multidirectional). In addition, we observed mRNA granules that reversed direction at dendritic branch points to move between different segments (Supplementary Movie 7). These transport behaviors support the sushi-belt model of dendritic mRNA trafficking, which proposes that mRNA granules patrol dendrites in a highly dynamic multidirectional fashion, without being irreversibly anchored at a single specific location^6^. We quantified the frequencies of these transport behaviors and found that half of the mRNA granules remained stationary during the 1 min acquisition period. In contrast, the mobile fraction of granules traversed the dendrites in a highly dynamic way, including unidirectional, interrupted, and multidirectional movements, independent of the 3′-UTR (Fig. 1f). Increasing the acquisition time to 10 min reduced the fraction of stationary and unidirectional granules in favor of interrupted and multidirectional movements, demonstrating that a large fraction of RNA granules indeed undergo transport in a sushi-belt like fashion. The fraction of the stationary population is consistent with previously published data for the *ß-actin* mRNA as well as for Staufen1 granules in the time frames analyzed^13,41^. Statistical analysis by the Chi-squared test could not establish any difference in the frequency of these events between *MS2 only* or *MS2+Rgs4* 3′-UTR reporter mRNAs, suggesting that the 3′-UTR of the transcript does not regulate the types of motility exhibited by the reporters.

As both *MS2+Rgs4* and *MS2 only* reporter mRNAs were found to be localized in dendrites, we decided to investigate the underlying regulation of dendritic mRNA sorting, which ultimately fine-tunes trafficking to achieve a specific localization upon demand. We examined multiple parameters of 3′-UTR-dependent mRNA granule transport, including speed, displacement and directionality in dendrites (Fig. 2; Supplementary Fig. 2; Supplementary Movie 8). When exploring transport directionality, the *MS2 only* mRNA displayed an equal number of mRNA granules moving in the anterograde (48.9 ± 1.2%) or retrograde direction (Fig. 2d, e). Interestingly, the *MS2+Rgs4* 3′-UTR mRNA mediated a significant anterograde transport bias, with 58.8 ± 2.9% of mRNA granules moving toward more distal dendritic regions (Fig. 2d, e; *p* = 0.0056). Moreover, when the percentage of total anterograde travel distance of all mRNA granules was investigated, we observed a similar transport bias for the *MS2+Rgs4* 3′-UTR (59.1 ± 2.5%) compared to the *MS2 only* (52.8 ± 2.6%) mRNA reporter (Fig. 2f, g; *p* *=* 0.0399). Directional transport has previously been observed for other mRNAs, such as *ß-actin*, *Arc*, and *CaMKIIα*, in hippocampal neurons or *oskar* in the *Drosophila* oocyte, with a preferential transport direction toward the distal or the posterior part of the cell, respectively^9,38,39,42^. In contrast, we observed no differences in either average transport speed or in average displacement length of single events, indicating that the *Rgs4* 3′-UTR did not affect these parameters of mRNA transport (Fig. 2h, i; Supplementary Fig. 2a, b). The distribution of single data points showed that most RNA granules underwent short displacement events and only few particles traveled long distances at a time, often longer than the 40 µm analyzed (Supplementary Fig. 2b). To exclude that the NLS fused to the tdMCP protein might potentially affect transport as previously reported^43^, we generated a tdMCP lacking the NLS and repeated the previous experiment. Although this yielded a higher fluorescent background, we still observed an anterograde transport bias mediated by the *Rgs4* 3′-UTR (Supplementary Fig. 2c, d), showing that the NLS did not affect trafficking in our hands.

In conclusion, our live-cell imaging data suggest that the *Rgs4* 3′-UTR was responsible for the observed anterograde transport bias, affecting both anterograde moving mRNA granule number and anterograde travel distance in dendrites. Importantly, the results establish the 3′-UTR as a key determinant, as the bias was not observed in the absence of the *Rgs4* 3′-UTR.

### Neuronal inhibition abolishes the *Rgs4* 3′-UTR transport bias

**Fig. 3:**
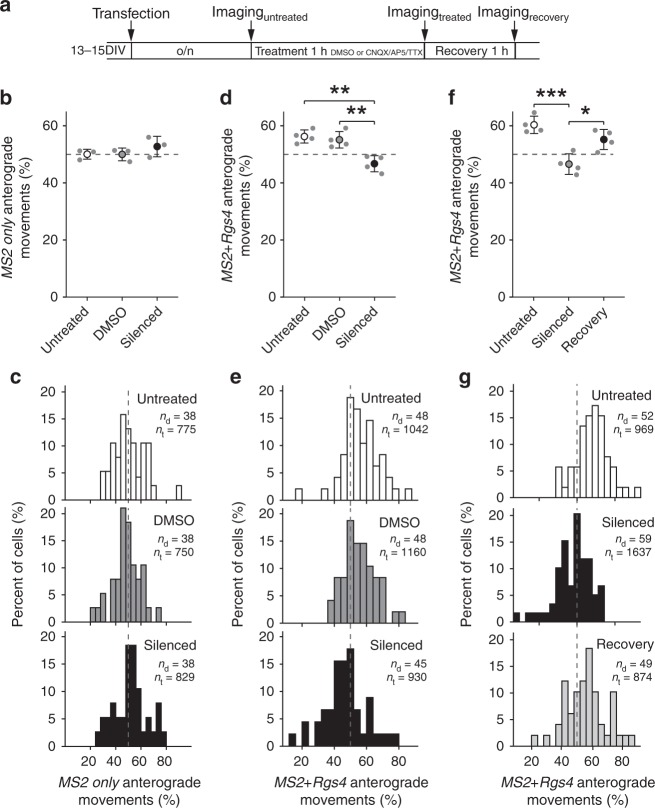
Inhibition of neuronal activity abolishes *Rgs4* 3′-UTR-dependent transport bias. **a** Scheme of experimental outline. Dot plots (**b**, **d**, **f**) and histograms (**c**, **e**, **g**) displaying percentage of anterograde moving *MS2 only* (**b**, **c**) or *MS2+Rgs4* 3′-UTR (**d**–**g**) reporter mRNA granules in rat hippocampal neurons, untreated, vehicle treated (DMSO) or silenced (100 µM CNQX, 50 µM AP5, 1 µM TTX) and after 1-h recovery. Data represent mean ± standard deviation of 3–4 independent experiments (individual experiments shown as gray dots). Asterisks represent *p*-values assessed by Tukey’s test post-hoc to one-way ANOVA analysis (*p < 0.05, **p < 0.01, ***p < 0.001). Data were obtained from 40-µm dendritic segments at a minimal distance of 20 µm from the cell body. At least 10 dendrites/condition/experiment were analyzed. Total number of dendrites (*n*_d_) and tracks (*n*_t_) analyzed per condition are indicated. Only displacements ≥1.5 µm were considered for analysis

Next, we asked whether neuronal activity might regulate dendritic mRNA transport. As mature neurons display endogenous neuronal activity in culture, we chemically silenced activity by simultaneously inhibiting AMPA receptors, NMDA receptors, and voltage-gated sodium channels via bath application of CNQX, AP5, and TTX^27^. Neurons transfected with either *MS2 only* or *MS2+Rgs4* 3′-UTR reporters were left untreated or preincubated for 1 h with either vehicle or CNQX/AP5/TTX, and then imaged during continuous treatment (Fig. 3a). No differences in speed, displacement, or transport directionality were observed for the *MS2 only* mRNA reporter, independent of treatment (Fig. 3b, c, Supplementary Fig. 3a, b, g, h; displacement (µm): untreated 5.6 ± 6.7, DMSO 5.5 ± 6.9, silenced 6.2 ± 7.1). However, when neuronal activity was inhibited the anterograde transport bias mediated by the *MS2+Rgs4* 3′-UTR was completely abolished (46.7 ± 2.3%), compared to vehicle treated (55.1 ± 2.9%, *p* *=* 0.00431) or untreated neurons (56.3 ± 2.3%, *p* *=* 0.00187) (Fig. 3d, e; *F*_2,9_ = 0.00136). Moreover, the inhibition of neuronal activity alleviated the anterograde bias observed in respect to total travel distance (50.2 ± 2.5%), compared to vehicle treated samples (56.0 ± 3.0%, *p* = 0.02884) (Supplementary Fig. 3c, d). Importantly, these effects were not due to neuronal toxicity, as 1 h wash-off of chemical inhibition induced recovery of the transport bias (*F*_2,9_ = 0.00093, *p* = 0.01456 for silenced vs wash-off, *p* = 0.00074 for silenced vs untreated, Fig. 3f, g). Anterograde displacement of *MS2+Rgs4* 3′-UTR mRNA granules partially recovered after 1 h wash-off (*F*_2,9_ = 0.021, *p* = 0.424 for silenced vs wash-off, *p* = 0.017 for silenced vs untreated, Supplementary Fig. 3e, f). Moreover, fractions of mRNA granule mobility, categorized as stationary, unidirectional, interrupted, and multidirectional, remained unaffected by inhibition of neuronal activity (Supplementary Fig. 3i, j). Evaluation of mRNA expression levels in cortical neurons by RT-qPCR and independent quantification of dendritic mRNA granules in single hippocampal neurons showed that neuronal inhibition did not affect either the amount of reporter mRNAs or mRNA granules analyzed (Supplementary Fig. 4a, b). Together, these data demonstrate that the transport bias of the reporter mRNAs not only depended on the *Rgs4* 3′-UTR, but on neuronal activity as well. Importantly, the movement of the *MS2 only* mRNA reporter remained unaffected by synaptic inhibition, suggesting that neuronal silencing did not reduce the mRNA transport bias in general, but that the effect was indeed dependent on the *Rgs4* 3′-UTR.

### *Rgs4* 3′-UTR-dependent mRNA sorting to synapses

**Fig. 4:**
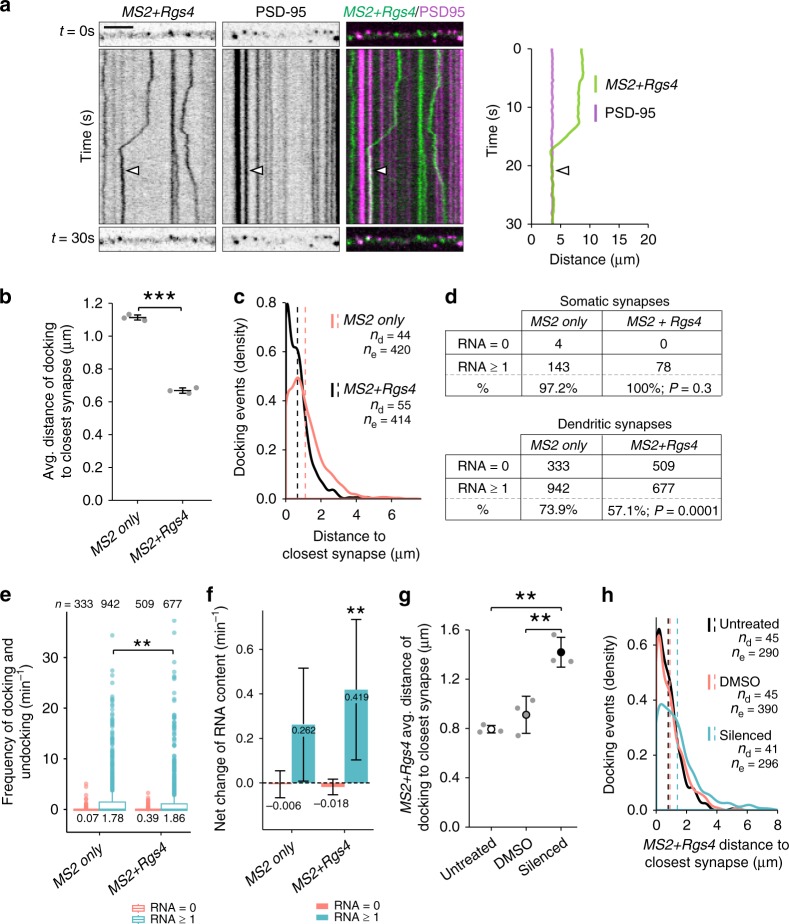
*Rgs4* 3′-UTR mediates mRNA recruitment to synapses dependent on neuronal activity. **a** Representative dual-color kymograph showing *MS2+Rgs4* 3′-UTR reporter mRNA (green) and PSD-95-tagRFPt (magenta) from a dendrite of a rat hippocampal neuron. First and last frames are shown at top and bottom. Scale bar 5 µm. Extracted track (right) of an mRNA granule docking at a PSD-95 positive area indicated by arrowheads. Distance of *MS2 only* or *MS2+Rgs4* 3′-UTR reporter mRNA docking events to closest PSD-95 positive cluster in neurons displayed as dot plot (**b**) and density plot (**c**). **d** Distribution of *MS2 only* or *MS2+Rgs4* reporter mRNA-positive (estimated RNA number ≥1 at *t* = 0 s) and -negative (RNA = 0 at *t* = 0 s) PSD-95-tagRFPt clusters in soma and dendrites. *P-values* of Chi^2^ tests against control are indicated. **e** Integrated frequency of reporter docking and undocking events at dendritic synapses. Number of observations and population means are indicated. **f** Average net change of *MS2 only* or *MS2+Rgs4* mRNA content at mRNA reporter-positive or -negative synapses per min, calculated from estimated reporter molecules docking or undocking at synapses per event. Numbers indicate mean value of net RNA level change. Error bars represent 95% confidence intervals. ** indicates significant (*α* = 0.01) difference compared to zero (null hypothesis, two sided one-sample *t*-test). Distance of *MS2+Rgs4* 3′-UTR reporter mRNA docking events to closest PSD-95 positive cluster under untreated, vehicle treated (DMSO) or silenced (100 µM CNQX, 50 µM AP5, 1 µM TTX) conditions displayed as dot plot (**g**) and density plot (**h**). Data represent mean ± standard deviation of three independent experiments (individual experiments shown as gray dots; **b**, **g**). Dashed lines represent mean values of single data points (**c**, **h**). Asterisks represent *p*-values obtained by Student’s *t* test (**b**), Mann Whitney *U* test (**e**) or Tukey’s test post-hoc to one-way ANOVA analysis (**g**) (**p < 0.01, ***p < 0.001). Data were obtained from 40-µm dendritic segments at a minimal distance of 20 µm from the cell body. At least 10 dendrites/condition/experiment (**a**–**c**, **g**, **h**) or 12 neurons/condition (**d**–**f**) from 3 independent biological replicates were analyzed. Total number of dendrites (*n*_d_), events (*n*_e_) and synapses (*n*) analyzed per condition are indicated

As inhibition of neuronal activity abolished the *Rgs4* 3′-UTR-dependent anterograde transport bias in distal dendrites, we next investigated whether mRNA was recruited to dendritic synapses. To visualize endogenous excitatory synapses in dendrites, we generated a fluorescent synaptic marker by tagging the postsynaptic density protein 95 kD (PSD-95) with tagRFPt (PSD-95-RFP). Mature neurons were cotransfected with PSD-95-RFP, tdMCP-GFP and either the *MS2 only* or *MS2+Rgs4* 3′-UTR mRNA reporters. To analyze whether synapses might affect mRNA granule transport, we coimaged both mRNA reporters and the synaptic marker by time-lapse dual-color microscopy in single cells for 1 min. We generated kymographs for each separate channel and created dual-color overlays (Fig. 4a; Supplementary Movies 9 and 10). We identified the positions, where mRNA granules were found to either interrupt (termed docking) or reinitiate transport (termed undocking), and measured the distance to the closest PSD-95-RFP positive cluster. Importantly, co-expression of the fluorescent reporters together with either *MS2 only* or *MS2+Rgs4* 3′-UTR reporter mRNA did not modify synaptic density (Supplementary Fig. 5a). Moreover, we found no difference in the ratio of mRNA granules docking or undocking between *MS2 only* and *MS2+Rgs4* 3′-UTR reporter mRNAs, respectively (Supplementary Fig. 5b). While both reporter mRNAs were recruited to synapses, *MS2+Rgs4* 3′-UTR reporter mRNAs on average docked closer than *MS2 only* reporter mRNAs (1.11 ± 0.01 µm for *MS2 only* vs 0.67 ± 0.02 µm for *MS2+Rgs4* 3′-UTR; *p* = 4.25E^−6^, Fig. 4b, c). Similar results were obtained for mRNA granules undocking after a previous stationary phase (Supplementary Fig. 5c, d). Together, these results suggest a dynamic sorting process as the *Rgs4* 3′-UTR mediated mRNA recruitment and eventual release close to synapses.

To further investigate how *MS2 only* and *MS2+Rgs4* 3′-UTR RNA reporters behave at the synapse, we acquired longer dual-color videos (3.5 min, at ~4.7 fps) of neurons cotransfected with either *MS2 only* or the *MS2+Rgs4*, tdMCP-GFP and the synaptic marker PSD-95-RFP. We tracked PSD-95-RFP positive clusters over time and measured the GFP fluorescence of reporter mRNAs in equivalent areas. This allowed us to specifically analyze dynamic changes in GFP fluorescence intensity caused by docking or undocking reporter mRNAs at single synapses (see “Methods” for details). During the time analyzed, we observed at least one docking or undocking event of either *MS2 only* or *MS2+Rgs4* reporter mRNAs at ~34% vs ~21% of dendritic synapses, respectively (Supplementary Fig. 5e). This represents a ~30% significant reduction in the frequency of these events for the *MS2+Rgs4* 3′-UTR reporter mRNA (*p* < 0.0001; Supplementary Figs. 5f and 6d). We found that the number of these events showed a moderate correlation (*R* ~ 0.5) with the estimated mRNA copy number within PSD-95-RFP positive synapses (Supplementary Fig. 5g). This suggests that the number of docking/undocking events is related with the total number of mRNA particles at synapses. Analysis of the postsynaptic sites revealed that mRNA-positive synapses were both larger and brighter in their mean PSD-95 signal intensity than mRNA negative synapses (*p* < 0.0001; Supplementary Fig. 5h). We found that the fraction of *MS2+Rgs4* 3′-UTR positive synapses in dendrites was significantly lower than that of *MS2 only* (57.1% vs 73.9%; *p* < 0.0001; Fig. 4d). However, when we focused on mRNA reporter-positive synapses, we found a significant but nonsubstantial difference between the frequency of total docking/undocking events of *MS2 only* and *MS2+Rgs4* 3′-UTR reporter mRNAs at synapses (*p* = 0.002; Fig. 4e).

Subsequently, we analyzed the net directionality of reporter mRNA docking or undocking events separately at postsynaptic densities. We found that the frequency of docking events was significantly higher for the *MS2+Rgs4*, compared to the *MS2 only* reporter (~0.9 min^−1^ for *MS2+Rgs4* vs 0.79 min^−1^ for *MS2 only*, *p* = 0.005), whereas there was no substantial difference in the frequency of undocking events (~0.99 min^−1^ for *MS2+Rgs4* vs 0.97 min^−1^ for *MS2 only*). In agreement with these results, we found a net influx (*p* = 0.009, *α* = 0.01) of *MS2+Rgs4* into mRNA-positive synapses, in contrast to the *MS2 only* reporter mRNA (Fig. 4f, Supplementary Fig. 4i), which was not significantly different from zero (*p* = 0.043, *α* = 0.01). In general, synapses contained mRNA at the beginning and during the experiment regardless of their location and reporter mRNA (Supplementary Fig. 6a–d).

In summary, these data demonstrate that the *MS2+Rgs4* 3′-UTR-mediated docking in closer proximity to synapses, compared to the *MS2 only* 3′-UTR. Furthermore, although the *MS2+Rgs4* reporter interacted with fewer synapses, it displayed a net increase at synapses, while the *MS2 only* reporter did not. These findings suggest that dendritically localized *MS2+Rgs4* mRNA was associated with a specific subset of synapses.

### Neuronal activity recruits *Rgs4* 3′-UTR mRNA to synapses

Next, we investigated whether inhibition of neuronal activity affected recruitment of *Rgs4* 3′-UTR mRNA to synapses in addition to its effect on transport directionality. Therefore, we chemically inhibited neuronal activity in mature rat hippocampal neurons transiently cotransfected with either the *MS2 only* or the *MS2+Rgs4* 3′-UTR mRNA, tdMCP-GFP and the synaptic marker PSD-95-RFP. Neither synaptic density nor the ratio of docking to undocking events was altered when silenced cells were compared with vehicle or untreated controls (Supplementary Fig. 7a, b). In addition, *MS2 only* mRNA granules did not exhibit any significant change in their distance to PSD-95-RFP synapses upon inhibition of neuronal activity when compared to vehicle or untreated cells (Supplementary Fig. 7c, d). However, the inhibition of neuronal activity increased the docking/undocking distance of *MS2+Rgs4* mRNA close to synapses, to values comparable to *MS2 only* mRNA (*F*_2,6_ = 0.001, *p* = 0.004 for silencing vs vehicle, *p* = 0.001 for silencing vs untreated, Fig. 4g, h; *F*_2,6_ = 0.001, *p* = 0.003 for silencing vs vehicle, *p* = 0.00162 for silencing vs untreated, Supplementary Fig. 7e, f).

**Fig. 5:**
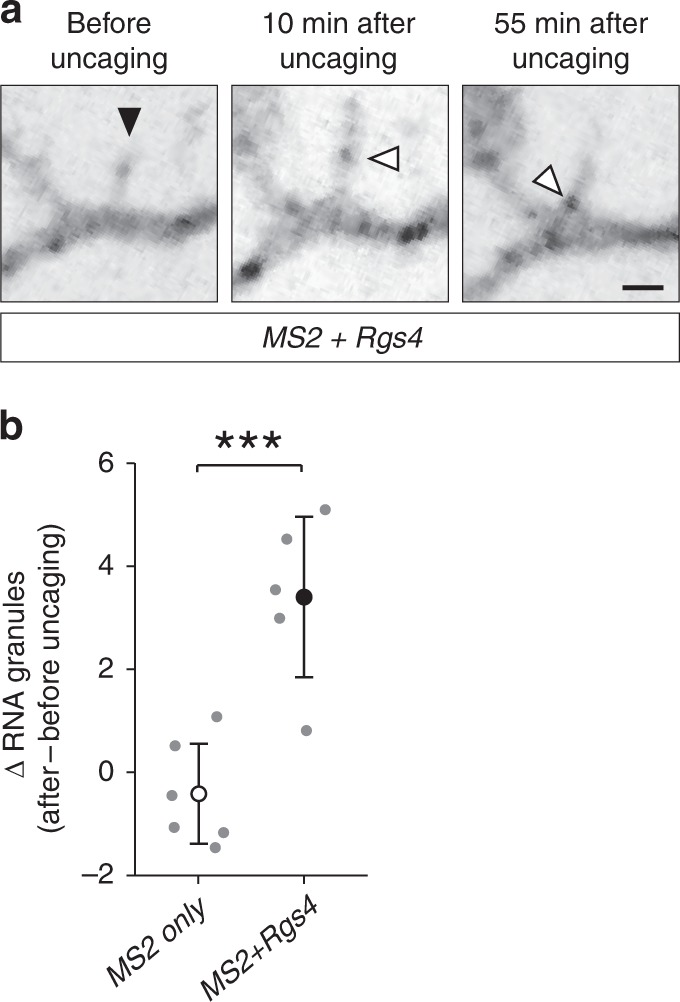
Local glutamate uncaging at spines triggers *Rgs4* 3′-UTR-dependent mRNA recruitment. **a** Representative GFP fluorescence of a hippocampal neuron cotransfected with the *MS2+Rgs4* 3′-UTR reporter and tdMCP-GFP constructs before (left panel) and after (middle, right panels) local glutamate uncaging. Black arrowhead denotes the uncaging spot at dendritic spine. White arrowheads indicate GFP positive *MS2+Rgs4* reporter mRNA granules. Scale bar 2 µm. **b** Dot plot displaying the change in RNA granule number 40–45 min after uncaging compared to before uncaging within 5 µm of the stimulated spine. Data represent mean ± standard deviation (individual neurons shown as gray dots). Asterisks represent *p*-values obtained by Student’s *t* test (***p < 0.001). Data were obtained from six dendrites for *MS2 only* (five neurons of four biological replicates) and five dendrites for *MS2+Rgs4* reporter mRNAs (five neurons of five biological replicates), respectively

Next, we performed local two-photon glutamate uncaging at individual dendritic spines to evaluate whether the stimulation of single spines would be sufficient to recruit mRNA granules. Either the *MS2 only* or the *MS2+Rgs4* reporter mRNAs were cotransfected with tdMCP-GFP and tandem Tomato (tdTomato). Upon glutamate uncaging adjacent to individual spines, we observed an increase in spine size by the volume marker (tdTomato) (Supplementary Fig. 8a). The mRNA granule number before and after uncaging was quantified within a 5 µm radius along dendrites centered at the stimulated spine. We observed an average increase of ~3 RNA granules for the *MS2+Rgs4* reporter mRNA, while there was no increase of *MS2 only* reporter granules (Fig. 5a, b, Supplementary Fig. 8b, Supplementary Movie 11). Together, these data demonstrate that neuronal activity is not only necessary to mediate the *Rgs4* 3′-UTR-dependent mRNA transport bias, but is also required to recruit its mRNA to activated synapses. Furthermore, we provide further experimental evidence that it is the *Rgs4* 3′-UTR that has a direct influence on activity-dependent mRNA docking/undocking, as the *MS2 only* reporter mRNA remained unaffected by neuronal inhibition or local stimulation of dendritic spines.

### Stau2 regulates dendritic transport via the *Rgs4* 3′-UTR

**Fig. 6:**
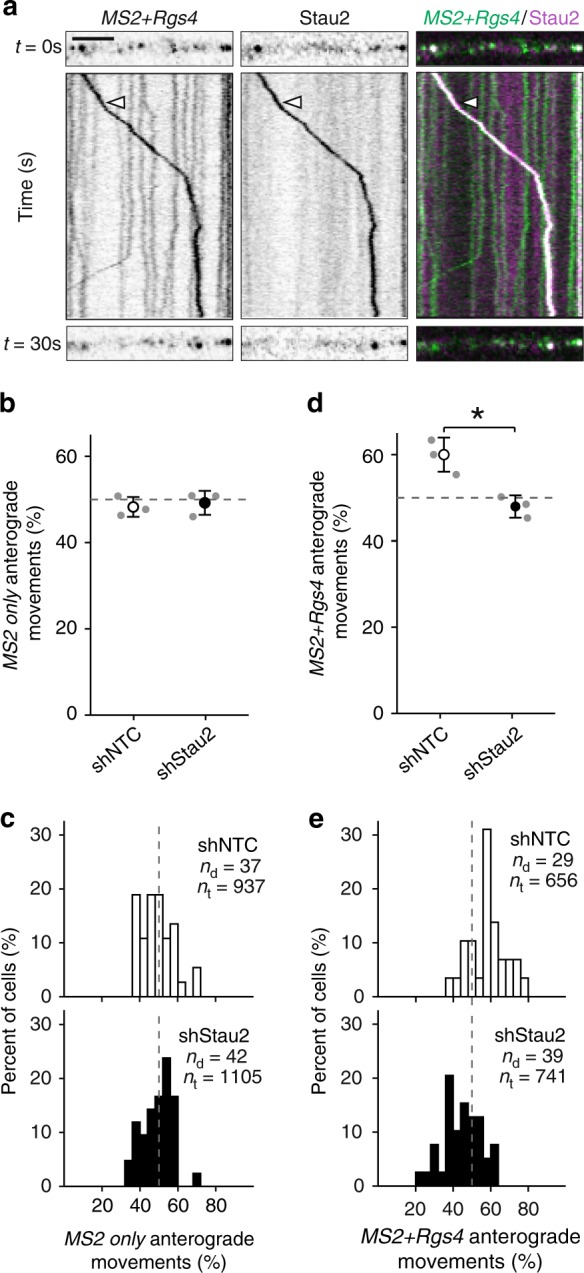
Stau2 regulates *Rgs4* 3′-UTR-dependent transport. **a** Representative dual-color kymograph showing *MS2+Rgs4* 3′-UTR reporter mRNA (green) and tagRFPt-tagged Stau2 (magenta) from a dendrite of a rat hippocampal neuron. First and last frames are shown at top and bottom. Arrowheads indicate an *MS2+Rgs4* reporter and Stau2 positive RNA granule undergoing cotransport. Scale bar 5 µm. Dot plots (**b**, **d**) and histograms (**c**, **e**) displaying percentage of anterograde moving *MS2 only* or *MS2+Rgs4* 3′-UTR reporter mRNA granules in shNTC and shStau2 transduced hippocampal neurons. NTC non-targeting control. Data represent mean ± standard deviation of three independent experiments (individual experiments shown as gray dots). Asterisks represent *p*-values obtained by Student’s *t* test (*p < 0.05). Data were obtained from 40-µm dendritic segments at a minimal distance of 20 µm from the cell body. Total number of dendrites (*n*_d_) and tracks (*n*_t_) analyzed per condition are indicated. Only displacements ≥1.5 µm were considered for analysis

Finally, we investigated whether the RBP Stau2, which is known to bind *Rgs4* mRNA^27,30^, was required for the transport of *MS2+Rgs4* granules. To examine whether mRNA is cotransported with Stau2, we cotransfected the *MS2+Rgs4* reporter with tdMCP-GFP and tagRFPt-tagged Stau2 (RFP-Stau2). We observed multiple instances, where the *MS2+Rgs4* reporter mRNA granules were cotransported with Stau2 (Fig. 6a, Supplementary Movies 12-13). Moreover, overexpression of Stau2 resulted in an increase of dendritic *MS2+Rgs4* reporter density, while the *MS2 only* mRNA was unaffected (Supplementary Fig. 9a). In addition, we performed an experiment involving *MS2* RNA-mediated tethering of Stau2 to the *MS2 only* reporter mRNA. When we cotransfected both tdMCP-Stau2 and tdMCP-GFP together with the *MS2 only* reporter, we observed that the tethering of Stau2 tended to recruit the control mRNA closer to the synaptic marker vesicular glutamate transporter 1 (VGLUT1) (Supplementary Fig. 9b). Together, our data suggest that Stau2 might indeed regulate *Rgs4* 3′-UTR-dependent recruitment to synapses. However, additional work is clearly necessary to substantiate these findings in the future.

To further investigate the involvement of Stau2 in dendritic mRNA transport, we transduced neurons with lentiviral particles expressing either a short hairpin nontargeting control (shNTC) or a short hairpin specific for *Stau2* (shStau2-2)^28^ 4 days prior to cotransfection with tdMCP-GFP and *MS2 only* or *MS2+Rgs4* 3′-UTR reporter mRNAs. Time-lapse imaging revealed that the *MS2 only* reporter mRNA remained unaffected by *Stau2* knockdown (Fig. 6b, c, Supplementary Fig. 9c, d). In contrast, *Stau2* knockdown abolished the anterograde transport bias of *MS2+Rgs4* 3′-UTR mRNA granules in distal dendrites (*p* = 0.012, Fig. 6d, e). Total anterograde displacement remained unaffected for *MS2 only* mRNA, but showed a nonsignificant reduction for the *MS2+Rgs4* 3′-UTR reporter in Stau2 deficient neurons (Supplementary Fig. 9e, f). Different types of *MS2+Rgs4* 3′-UTR mRNA granule mobility, categorized as stationary, unidirectional, interrupted and multidirectional, as well as speed and displacement length were unaffected by *Stau2* knockdown (Supplementary Fig. 9g; speed (µm s^−1^): shNTC 3.2 ± 1.3, shStau2 2.9 ± 1.2; displacement (µm): shNTC 6.9 ± 7.5, shStau2 6.5 ± 6.5). The reduction of Stau2 expression was verified in the imaged samples by immunostaining (Supplementary Fig. 10a). Moreover, quantification by RT-qPCR revealed that *Stau2* knockdown did not affect the expression levels of reporter mRNAs (Supplementary Fig. 10b).

In conclusion, these data provide direct experimental evidence that Stau2, which is cotransported together with *Rgs4* reporter mRNA in distinct RNA granules, is responsible for the observed 3′-UTR-dependent transport bias.

## Discussion

Here, we show that mRNAs travel along dendrites in a dynamic and multidirectional fashion, a process that does not exclusively require a specific 3′-UTR. The presence of the *Rgs4* 3′-UTR, however, conveyed key properties to the reporter mRNA, which fine-tune transport as (i) it introduced a transport bias directed toward distal dendrites, and (ii) regulated the spatio-temporal association with synapses. This was dependent on neuronal activity and the RBP Stau2. Together, our data not only provide experimental support for the sushi-belt model of dendritic mRNA transport^6^, in which mRNAs dynamically patrol synapses, but also allow us to advance this model by the introduction of a regulated transport bias. This sheds mechanistic insight into global RNA sorting processes, including recruitment of RNAs by individual stimulated synapses.

Here, we present evidence that mRNA granules traverse distal dendrites in a directed manner, independently of the presence of a specific 3′-UTR. All *MS2 only*, *MS2+Rgs4* and *MS2+histone-3.3* 3′-UTR mRNA reporters formed dendritic mRNA granules, suggesting that additional factors other than 3′-UTR sequences contribute to dendritic localization. For instance, the poly-A-tail or other mRNAs contained in the same granule^3^ might be involved. However, independently of the 3′-UTR sequence, a proportion of the observed RNA granules remained stationary, while others showed variability in speed, displacement length, and directionality of transport. In addition to both simple anterograde and retrograde transport, some mRNA granules displayed more complex dynamics. Indeed, some granules interrupted and subsequently reinitiated transport or even switched direction without interruption. This interrupted transport has recently been investigated and computationally modeled, providing a basis for future functional studies^44^. In addition, we show here that longer observation time revealed an increase in interrupted movements and a decrease in unidirectional or stationary phases, demonstrating that a majority of mRNA granules may undergo multiple transport phases in different directions. This observation expands our current understanding of mRNA sorting beyond local recruitment, as it proves experimentally that mRNA granules are not irreversibly anchored, but are dynamic, can be targeted to specific sites upon demand, and be released from them later on. Taken together, we provide experimental evidence that the sushi-belt model accurately describes dendritic mRNA transport in live neurons. However, we do not exclude that other factors/mechanisms might contribute as well. Interestingly, a recent computational study provides evidence that the sushi-belt model can achieve complex spatial distribution of cargo in neurons^45^.

A key finding of the current study is that the 3′-UTR of *Rgs4* mRNA mediated an anterograde transport bias in dendrites, i.e. preferential transport directionality toward distal regions. In contrast, neither the *MS2 only* nor the *MS2+histone-3.3* reporter mRNAs displayed this bias (Fig. 2d; *MS2+histone-3.3* anterograde movement (%): 51.2 ± 0.3). Previous studies have observed both anterograde and retrograde mRNA transport. However, a directional bias in transport has been reported only in few cases, e.g. for *oskar* mRNA in the *Drosophila* oocyte, or for *ß-actin* and *Arc* mRNAs in mouse hippocampal neurons^9,38,42,46^. It is worth mentioning that these studies observed a bias of similar magnitude as we report here (~60% toward distal regions), indicating that the dendritic bias of the *MS2+Rgs4* 3′-UTR reporter is within a physiological range.

Interestingly, silencing of neuronal activity abolished the *MS2+Rgs4* 3′-UTR specific transport bias, while the transport of the *MS2 only* reporter remained unaffected. Endogenous neuronal signaling in culture restored the bias, demonstrating its physiological pertinence. In contrast to our observations, *Arc* mRNA transport bias was not affected by neuronal activity^46^. This suggests that anterograde transport is differentially regulated depending on mRNA sequence and that distinct RNA granules may be differently regulated by neuronal activity. Furthermore, we show that knockdown of Stau2 abolished the *Rgs4* 3′-UTR-dependent anterograde transport bias as well. As the *MS2 only* reporter mRNA remained unaffected by Stau2 knockdown, the loss of the anterograde transport bias is specific to the *Rgs4* 3′-UTR and might be caused by the absence of Stau2 in *Rgs4* containing mRNA granules. Therefore, we hypothesize that Stau2 might be recruited in conjunction with neuronal activity to modulate dendritic *Rgs4* mRNA transport. Similarly, previous research has shown how Staufen and other RBPs are not necessary for general transport, but can facilitate or modulate it^47,48^. Along this line, Staufen has been implicated in kinesin-1-dependent posterior localization of *oskar* mRNA in *Drosophila* and has been found in a complex with kinesin-1 in *Xenopus*^47,48^. In line with this finding, we observed directed cotransport of *MS2+Rgs4* granules together with Stau2 in neurons. Importantly, Stau2 depletion results in both morphological and physiological synaptic phenotypes^28,49^. Therefore, it is tempting to speculate that Stau2 might not only regulate the expression of proteins relevant at synapses as previously shown^30^ but also the transport and recruitment of their mRNAs. In turn, deregulation of synaptic proteins, resulting in aberrant synaptic remodeling, might render a synapse incapable to properly recruit relevant transcripts. Together, our data give functional insight into the regulation of 3′-UTR-dependent mRNA transport. We show that it is the *Rgs4* 3′-UTR that facilitates dendritic localization via the anterograde transport bias. Although dendritic sorting might be affected by other factors such as regulated mRNA degradation, this transport bias might enable fast and efficient mRNA recruitment to specific regions such as synapses as needed. Future studies will have to unravel the detailed molecular mechanisms of how Stau2, neuronal activity and 3′-UTR sequences cooperate to mediate this anterograde bias in neurons.

Another key observation is that the *MS2+Rgs4* 3′-UTR facilitated mRNA docking and undocking in closer proximity to synapses compared to *MS2 only*. Upon silencing of endogenous neuronal activity, the distance of docking/undocking was increased for the *MS2+Rgs4* 3′-UTR, while the *MS2 only* reporter remained unaffected. Conversely, local stimulation of individual dendritic spines by glutamate uncaging resulted in increased recruitment of *MS2+Rgs4* granules, but not *MS2 only* mRNAs. Furthermore, the *MS2+Rgs4* 3′-UTR reporter interacted with fewer synapses and displayed more docking events at dendritic synapses than the *MS2 only* reporter. This led to a slower turnover of *MS2+Rgs4* mRNA content at synapses compared to *MS2 only*, indicating *MS2+Rgs4* is more stably associated and might remain at synapses longer. We propose that the presence of *MS2 only* mRNA at synapses might represent a state of nonspecific, default localization. These effects, along with the observed differences in the brightness of PSD-95 clusters could reflect differences in either synaptic activity or in the subtype of synapses. As the Rgs4 protein is a negative regulator of synaptic activity, we speculate that its mRNA is transported to a subtype of synapses, where it might be regulated in situ. Here, the mRNA could be unpacked and locally translated by polysomes localized close to PSD-95 clusters^50^, where the newly synthesized Rgs4 protein would modulate GPCR-mediated neuronal signaling. Our findings are in agreement with a study by the Singer lab that showed that endogenous *ß-actin* mRNA is recruited to glutamate-stimulated dendritic spines, where it is locally translated^13^. Using a similar approach, we now show that glutamate stimulation of individual spines results in 3′-UTR-dependent recruitment of *Rgs4* mRNA, as the *MS2 only* reporter displayed no variation in recruitment.

In conclusion, we hypothesize that the observed anterograde transport bias contributes to synaptic recruitment of mRNAs. As both the transport bias and synaptic recruitment are modulated by the 3′-UTR and neuronal activity, anterograde transport might indeed facilitate synaptic recruitment, especially at endogenous mRNA expression levels. Future work will aim to understand how neuronal activity affects the organization of key cytoskeletal components to mediate *Rgs4* mRNA recruitment and whether it affects their capture on ribosomes at synapses.

We therefore propose a model of *Rgs4* trafficking in neuronal Stau2 mRNA granules, in which its 3′-UTR specifically mediates sorting to distal dendrites. In this model, the *Rgs4* mRNA patrols the dendrite in a dynamic fashion in accordance with the sushi-belt model^6^. Neuronal activity can result in the docking of this mRNA at specific postsynaptic sites and cause unpacking of the mRNA from transport granules. There, the mRNA may be subjected to local translation, making the encoded Rgs4 protein available in a spatially and temporally restricted manner. After the mRNA has fulfilled its function at the synapse, it may undock and reinitiate transport until it is degraded or recruited for a new round of translation. Such processes are the basis of cellular mechanisms in polarized cells involved in, e.g. dendritic arborization, long-term potentiation, and synaptic plasticity and are indispensable for neuronal development, learning, and memory formation.

## Methods

### Neuronal cell culture, transfection and transduction

Rat hippocampal neuronal cell cultures^51^ were generated as follows: hippocampi of embryonic day 17 (E17) embryos of timed pregnant Sprague-Dawley rats (Charles River Laboratories) were isolated, cells dissociated with 2.5% trypsin and plated on poly-l-lysine coated coverslips or glass bottom dishes (WillCo Wells) and cultured in NMEM+B27 medium (Invitrogen) at 37 °C with 5% CO_2_. In case of cortical cultures, E17 cortices were dissociated, the cell suspension sequentially filtered through 100- and 70-μm filters and then plated at a density of 700 cells/mm^2^. Experiments were performed with cultured neurons between 10 and 16 days in vitro (DIV). Neurons were transiently transfected by calcium phosphate coprecipitation^52^ at 10–15 DIV and imaged at 11–16 DIV. For each transfection condition 3–5 μg of DNA was mixed with 6 μl of 2.5 M CaCl_2_, in a final volume of 60 μl. Then, 60 μl of 2× BES buffer (pH = 7.00–7.20) was added to the mixture dropwise and air bubbles were introduced to the solution with the pipette. Each component was added stepwise and slowly. This mixture was added to the neurons and the precipitate was incubated with the cells under atmospheric CO_2_ concentration for 1 h at 37 °C. For experiments involving glutamate uncaging, neurons were plated on poly-l-lysine and laminin (Invitrogen) coated 12-mm-diameter glass coverslips, at a density of 600 cells/mm^2^. Cells were transfected at 15–17 DIV by calcium phosphate coprecipitation and imaged the following day. To knockdown Stau2 expression, 10–11 DIV neurons were transduced overnight with lentiviral suspension, transfected with MCP-GFP and MS2 mRNA reporter constructs at 14–15 DIV and imaged at 15–16 DIV or lysed for RNA extraction. All animals were used according to the German Welfare for Experimental Animals (LMU Munich, *Regierung von Oberbayern*).

Human embryonic kidney 293T (HEK-293; ATCC CRL3216) cells were grown in Dulbecco’s modified Eagle’s medium (DMEM; Invitrogen) supplemented with 10% fetal bovine serum (FBS; Invitrogen), stable 1% l-alanyl-l-glutamine (Biochrom) and 1% sodium pyruvate (Sigma Aldrich), and incubated at 37 °C with 5% CO_2_. One day before transfection, HEK-293 cells were plated in six-well plates at 5 × 10^4^ cells/well and transfected with indicated plasmids using calcium phosphate protocol.

### Plasmids

RNA reporter constructs were placed under the control of an RSV promoter, together with *LacZ* as ORF, a stop codon, and an array of either 32 unique MS2 hairpins or a quadruplication of this array, i.e. 128 MS2 hairpins^36^. The 3′-UTR was either omitted (pRSV-LacZ-MS2) or included (pRSV-LacZ-MS2-*Rgs4* 3′-UTR or pRSV-LacZ-MS2-*H3.3* 3′-UTR) prior to the poly-A signal. Sequences of the 3′-UTRs correspond to the positions 728–2919 nt of rat *Rgs4* mRNA (NM_017214.1) or 537–1087 nt of rat *Histone3.3* mRNA (X73683.1). To generate the pUBC-NLS-ha-tdMCP-GFP plasmid, the nls-HA-tdMCP-gfp sequence from the phage-ubc-nls-ha-tdMCP-gfp construct (Addgene #40649)^37^ was cloned into the pEGFP-C1 vector (Clontech). The pUBC-ha-tdMCP-GFP plasmid was generated by removing the NLS from the original vector. The pUBC-NLS-ha-tdMCP and pUBC-NLS-ha-tdMCP-Stau2 vectors were generated by extracting the UBC-NLS-ha-tdMCP sequence from the original vector and cloning the 62-kD isoform of mouse Stau2 in frame in the pEGFP-C1 vector (Clontech) in place of EGFP. The CMV-PSD95-tagRFPt vector was generated by cloning the PSD-95 ORF into the ptagRFPt-N1 vector (Robert H. Singer, USA). The pCMV-tagRFPt-Stau2 vector was generated by cloning the ORF of the 62-kD isoform of mouse Stau2 into the ptagRFPt-C1 vector (Robert H. Singer, USA). The pCMV-tdTomato vector was generated by cloning the ORF of tdTomato (Dieter Edbauer, Germany) into the ptagRFPt-C1 vector (Robert H. Singer, USA) in place of tagRFPt. The lentiviral packaging plasmids psPAX2 and pcDNA3.1-VSV-G have previously been described^30^. Lentiviral plasmids pFu3a-H1-sh-NTC-pCaMKIIα-tagRFP and pFu3a-H1-sh-Stau2-2-pCaMKIIα-tagRFP were generated by exchanging the UBC promoter for the CaMKIIα promoter from previously published FUW based vectors^30^.

### Lentivirus production

Control sh-NTC and sh-Stau2-2 lentiviral particles were generated from HEK-293 cells cotransfected with the plasmids psPAX2, pcDNA3.1-VSV-G and either pFu3a-H1-sh-NTC-pCaMKIIα-tagRFP or pFu3a-H1-sh-Stau2-2-pCaMKIIα-tagRFP, respectively, using calcium phosphate coprecipitation. Supernatants were filtered (0.45 µm RVDF Millex-HV; Millipore), concentrated by ultracentrifugation (65,000 *g*, 140 min, SW 32 Ti rotor; Beckman Coulter) and resuspended in Opti-MEM™ (Life Technologies)^30^.

### Single molecule FISH

Single molecule fluorescent in situ hybridization (smFISH)^40^ was performed with slight modifications. Briefly, cells were fixed in 4% paraformaldehyde (PFA) for 20 min and permeabilized in 70% ethanol overnight at 4 °C, followed by two rounds of DNase treatment for 1 h each at 37 °C to remove plasmid DNA. Hybridization of ten unique Cy3-labelled antisense-MS2 probes was performed overnight at 37 °C. Coverslips were mounted using Prolong Gold antifade mounting medium (Invitrogen). The following set of ten Cy3 conjugated probes were used (5′–3′, X = aminoallyl-modified T):

Probe 1 (nt 19–65):

AXCGAGCGCATAAACCCXAATGGTGTTTACAAATGGXGGTAGTCCTACCXA;

Probe 2 (nt 86–130):

AXAAACGACCAGAGXGTATTTCTCTCTGATACGCXGCGTACTCGTCAXA;

Probe 3 (nt 136–180):

AXATTGTGCGGTCGCXGACTGATACTTCTAGXCATCCGTTTGTCTAGXA;

Probe 4 (nt 186–231): AXGCTTGTAGTCAXAGCCTTAGCTTGGGTTATTACXCCAAGATCACCGXA;

Probe 5 (nt 238–282): AXGGGTGGAAGCCTTACXGATGCTTCCGGTCCATXCTAATACTATGGXA;

Probe 6 (nt 293–340):

AXCCAGTAGTCTTGGACCCCXTGAATACTACTGTTATTCXAATCCGTCACXA;

Probe 7 (nt 29–74): AXCCTAGTGGTACGCAGAXCATACCGTATTCGTGTAXGATTACATGGGXA;

Probe 8 (nt 80–124): AXAATCATTCTAGTGAXATGATTCTGTGCCGCXACTGCTGGCACCGTXA;

Probe 9 (nt 130–175): AXCGTCCTGATAGGCXGTACTCATGCCTACAACCXTCGATAATTCTGAXA;

Probe 10 (nt 184–229):

AXGTGTATTCATCTXAGCTGAGTGCTCTAAXGATGCACTACAGGACGCXA.

### Immunostaining

For immunostaining^28^ neurons were washed with warm Hanks′ Balanced Salt Solution (HBSS) and then fixed with warm 4% PFA in HBSS for 10 min. Fixed cells were washed with HBSS and permeabilized with 0.1% Triton X-100 in HBSS for 5 min. The following antibodies were used: (i) polyclonal antibodies, i.e. selfmade rabbit anti-Stau2^53^, guinea pig anti-VGLUT1 (Synaptic Systems, 419005); (ii) secondary antibodies, i.e. donkey anti-rabbit and goat anti-guinea pig Alexa555 or Alexa647 conjugated (Life Technologies, A31572, A31573, A21450). Coverslips were mounted on slides with Prolong antifade mounting medium (Invitrogen).

### Chemical treatments

To inhibit neuronal activity, cells were preincubated with 100 µM 6-cyano-7-nitroquinoxaline-2,3-dione (CNQX; Sigma, #C127), 50 µM 2-amino-5-phosphonopentanoic acid (AP5; Sigma, #A8054) and 1 µM tetrodotoxin (TTX; Abcam, #ab120055) in NMEM+B27 for 1 h at 37 °C. Media were exchanged for HBSS supplemented with 20 mM HEPES pH = 7.3, 100 µM CNQX, 50 µM AP5 and 1 µM TTX prior to imaging at the microscope. Vehicle treated cells were incubated with equivalent amount of DMSO. For cortical neurons, transfected cells were treated for 4 h with the aforementioned conditions and then lysed.

### RNA extraction, cDNA synthesis and qRT-PCR

Total RNA was extracted with the RNeasy Mini Kit (Qiagen). cDNA was generated from 400 ng of total RNA, using Superscript III^TM^ reverse transcriptase (Invitrogen) and 2.5-μM T20 primers according to the manufacturer’s instructions (+RT). As control for the presence of genomic DNA, equivalent reactions were made in the absence of reverse transcriptase (−RT). Quantitative real time PCR (qRT-PCR) was performed in duplicate from a 1:5 dilution of the stock cDNA, with a home-made SYBR Green Master Mix, with the LightCycler 96 System (Roche). The concentration of primers in the reaction was previously optimized to achieve 95–105% efficiency. Five microliters of an equimolar solution containing both forward and reverse primers were added to the reaction. Relative differences in RNA levels were calculated according to the 2^−ΔΔCt^ method^54^ and normalized against either *18*S ribosomal RNA, *peptidylprolyl isomerase A* (*ppia*) mRNA or *Kanamycin-resistance* (*Km*) mRNA. The sequences of the primers were (5′–3′): 18S_f: GAAACTGCGAATGGCTCATTAAA; 18S_r: CCACAGTTATCCAAGTAGGAGAGGA; ppia_f: GTCAACCCCACCGTGTTCTT; ppia_r: CTGCTGTCTTTGGAACTTTG; KmR_f: TGCCTGCTTGCCGAATATCA; KmR_r: ATATCACGGGTAGCCAACGC; LacZ_f: CGCTGGATAACGACATTGGC; LacZ_r: TCGTAATCAGCACCGCATCA; Stau2_f: GAACATCTCCTGCTGCTGAAG; Stau2_r: ATCCTTGCTAAATATTCCAGTTGT.

### Microscopy

Live-cell imaging was performed on a Zeiss Cell Observer spinning disk system consisting of a Zeiss Z1 Axio Observer microscope including a Plan-Apochromat 63x objective, a Yokogawa CSU-X1 spinning disk unit with 4 laser lines (405 nm 20 mW; 488 nm 50 mW, 561 nm 75 mW and 638 nm 75 mW) and an Evolve 512 Delta EMCCD Camera. For temperature control, a custom made EMBL environmental chamber (EMBLEM) was used. A 523/610 HC dual-band filter (AHF) was applied to reduce acquisition delay between channels during dual-color imaging. Hippocampal neurons were imaged at 36 °C in HBSS (Life Technologies) supplemented with 20 mM HEPES buffer pH 7.3 (Sigma Aldrich). Time-lapse images were acquired for the duration of 1, 3.5, or 10 min, with an approximate frame rate of ~15.3 fps for single channel acquisitions and ~4.7 fps for two-channel acquisitions with a 80 ms delay between channels. Cells were selected for proper expression of plasmids as well as for cell morphology and cell viability.

Imaging of fixed cells was performed on a Zeiss Z1 Axio Observer microscope including a Plan-Apochromat 63x objective, a COLIBRI.2 LED or a HXP 120 C light source and the Axiocam 506 mono camera.

Two-photon imaging and glutamate uncaging^55,56^ were carried out by using a single laser (Mai Tai HP; Newport-Spectra Physics, Santa Clara, CA, USA), which was tuned to 930-nm excitation wavelength for 2-photon imaging and 720 nm for uncaging. In brief, recordings were performed at 35 °C in ACSF (in mM: 127 NaCl, 2.5 KCl, 25 NaHCO_3_, 1.25 NaH_2_PO_4_, 4 CaCl_2_, 25 d-glucose; in µM: 1 TTX, 10 D-serine; pH 7.4; saturated with carbogen) on a custom two-photon laser-scanning microscope (objective: 60×, 0.9 numerical aperture; Olympus, Tokyo, Japan). In some experiments, the ACSF contained 2 mM CaCl_2_, 1 mM MgCl_2_ and no TTX and d-serine. Data obtained under these conditions were similar and pooled. MNI-caged l-glutamate was applied in the bath solution at 1.25 or 2.5 mM. Uncaging protocol: 30 pulses at 0.5 Hz, 4 ms pulse duration, wavelength 720 nm, 20 mW at the objective back aperture. At least six baseline image stacks (256 × 256 pixels, pixel size 0.105 or 0.125 µm) were recorded every 30 s on two channels (GFP, tdTomato) before glutamate uncaging was performed close to the dendritic spine to be stimulated. At 120 s or 150 s after stimulation, time-lapse imaging was resumed every 30 s until 5 min after stimulation, then every 60 s until 30 min and continued every 5 min until 60 min after stimulation.

### Image data analysis

Time-series image data of reporter mRNAs was analyzed by kymographs. Dendritic 40-µm segments at ≥20 µm from the cell body were selected and straightened in ImageJ. The KymographTracker plugin of the ICY Bioimaging software^57,58^ was used to generate kymographs and to trace and extract single tracks. Only movements longer than 1.5 µm were considered for analysis. Tracks were terminated when a particle stopped, changed direction or left the region of interest (ROI). Average speed and displacement were obtained by calculating the mean. Anterograde and retrograde tracks were counted to calculate the percent of anterograde transport. The sum of anterograde and retrograde displacement lengths was used to calculate the percent of total anterograde displacement. Dual-color kymographs were generated by overlaying identical regions of interest from two separate channels. Events in dual-color kymographs were manually selected and distances were manually measured in ImageJ (line tool), aided by a custom written ImageJ macro script (available upon request). Data were processed and subjected to statistical analyses in R^59^.

RNA signal intensity of PSD-95 labelled postsynaptic densities was analyzed within the synaptic masks generated by the xsParticle Tracker ImageJ plugin^60,61^.Time series of the measured, background corrected reporter RNA signal was fitted with a series of constants using the rpart package of R^62^. Minimum duration of a single constant fit was set to five frames (~1 s). The 5th percentile of the RNA signal intensity distribution measured in every 100 frames (for correction of photobleaching, Supplementary Fig. 6e, f) was used as the signal corresponding to a single reporter RNA molecule. Changes between two adjacent fitted constants, whose absolute value exceeded this threshold were quantified as docking and undocking events, depending on the sign of the change.

For deconvolution, z-stacks with 25 images at an interval of 0.26 µm were acquired, covering a total distance of 6.24 µm. Z-stacks were subjected to deconvolution using the constrained iterative quantitative restoration method of the Zeiss ZEN software deconvolution module.

For analysis of time-lapse series with glutamate uncaging, individual frames from image stacks were median filtered (5 × 5 pixel window) and maximum intensity projections generated. Dendritic spine size (tdTomato fluorescence) was determined as integrated fluorescence within a ROI containing the spine and subtracting integrated background signal from a ROI of the same size placed outside of any structure. To control for stimulus specific changes in spine size, the size of neighboring unstimulated spines or of an adjacent dendritic region was determined in the same way. Data were analyzed with custom routines written in MATLAB (version R2018b, MathWorks, Natick, MA, USA). mRNA granules were manually quantified in a 5-µm dendritic radius centered at the stimulated spine. To compensate for fluctuations due to ongoing transport two time points before (~7–4 and ~2 min) and after (40 and 45 min) uncaging were averaged respectively.

### Statistical analysis

R software or DeltaGraph Prism v8.0 were used for all data processing, plotting and statistical analysis^59,63–65^. Figures represent mean ± standard deviation of at least three independent biological replicates, unless otherwise stated. Asterisks represent *p*-values obtained by either Student’s *t* test, Tukey’s test post-hoc to one-way ANOVA analysis using the average values per experiment or pairwise Mann–Whitney *U* tests (*p < 0.05, **p < 0.01, ***p < 0.001), as indicated. The *F*-value evaluates whether the variance between the means of populations is significantly different (Fisher–Snedecor’s F distribution). The degrees of freedom are indicated as subscript. Significant levels (*α*) are provided for Fig. 4f and Supplementary Fig. 4i.

### Reporting summary

Further information on research design is available in the Nature Research Reporting Summary linked to this article.

## Supplementary information

Supplementary Information

Peer Review

Reporting Summary

Description of Additional Supplementary Files

Supplementary Video 1

Supplementary Video 2

Supplementary Video 3

Supplementary Video 4

Supplementary Video 5

Supplementary Video 6

Supplementary Video 7

Supplementary Video 8

Supplementary Video 9

Supplementary Video 10

Supplementary Video 11

Supplementary Video 12

Supplementary Video 13

## Data Availability

A reporting summary for this Article is available as a Supplementary Information file. The source data underlying all figures are provided as a Source Data file. K.E.B. live-cell imaging reveals 3′-UTR-dependent mRNA sorting to synapses (2019). Available at: osf.io/5ck7w. All data are available from the corresponding author upon request.
