# Supplementary material for: Live cell imaging reveals 3′-UTR dependent mRNA sorting to synapses
Source: Nat Commun. 2019 Jul 18;10:3178. doi: 10.1038/s41467-019-11123-x (PMC6639396; doi:10.1038/s41467-019-11123-x)
Supplement: Supplementary file 1 — Supplementary Information [file 41467_2019_11123_MOESM1_ESM.pdf]

Supplementary information

Live cell imaging reveals 3´-UTR dependent mRNA sorting to synapses

Karl E Bauer, Inmaculada Segura, Imre Gaspar, Volker Scheuss, Christin Illig, Georg Ammer, Saskia Hutten, Eugénia Basyuk, Sandra M Fernández-Moya, Janina Ehses, Edouard Bertrand and Michael A Kiebler

This file contains:

- 10 Supplementary Figure legends
- 10 Supplementary Figures

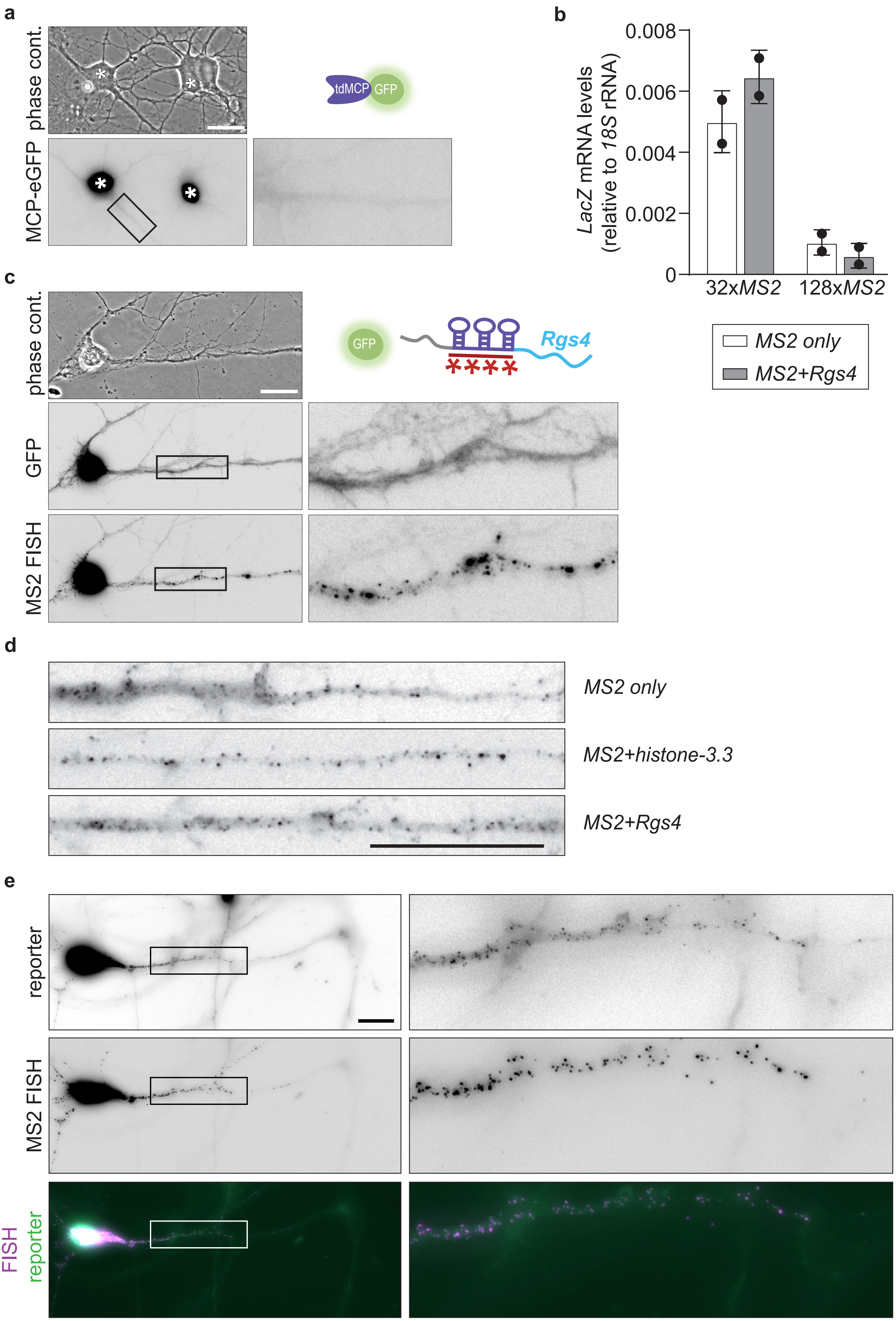
a
phase cont.
MCP-eGFP
tdMCP
GFP
b
LacZ mRNA levels
(relative to 18S rRNA)
0.008
0.006
0.004
0.002
0
32xMS2
128xMS2
MS2 only
MS2+Rgs4
c
phase cont.
GFP
MS2 FISH
GFP
Rgs4
d
MS2 only
MS2+histone-3.3
MS2+Rgs4
e
reporter
MS2 FISH
FISH
reporter

Supplementary Fig. 1 tdMCP-GFP positive granules contain MS2 reporter mRNA and localize to dendrites. **a** Phase contrast and GFP fluorescence of rat hippocampal neurons expressing tdMCP-GFP (scheme top right). Asterisks denote transfected cells. **b** Bar plot displaying *LacZ* mRNA reporter levels evaluated by RT-qPCR from HEK-293 cells transfected with either the *MS2 only* or *MS2+Rgs4* reporters containing either 32x*MS2* or 128x*MS2* repeats. Data represents mean ± standard deviation of 2 independent experiments (individual experiments shown as black dots). **c** Phase contrast, GFP fluorescence and MS2 single molecule FISH of rat hippocampal neurons co-expressing control GFP (not fused to MCP) and the *MS2+Rgs4* reporter mRNA (scheme top right). **d** Straightened dendritic segments, 60 µm from soma expressing tdMCP-GFP and either *MS2 only*, *MS2+histone-3.3* or *MS2+Rgs4* 3´-UTR reporter mRNAs. **e** GFP fluorescence (reporter), MS2 single molecule FISH and overlay in a rat hippocampal neuron co-transfected with tdMCP-GFP and *MS2+Rgs4* reporter mRNA. Unprocessed image of deconvolved data shown in Fig. 1B. Scale bars 20 µm. Related to Fig. 1.

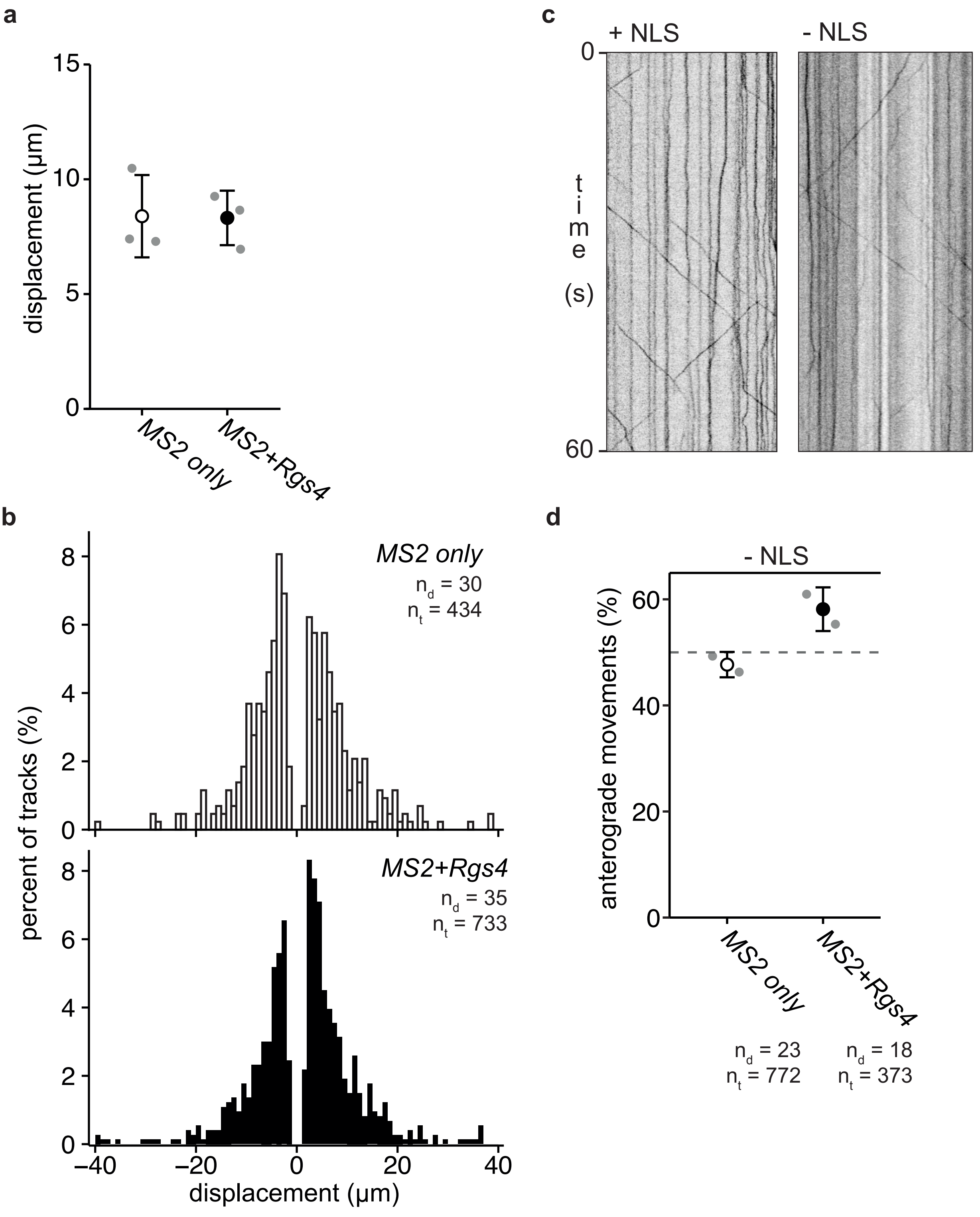

SUPPLEMENTARY FIGURE 2

Supplementary Fig. 2 Displacement and anterograde bias trafficking reporter mRNA granules. **a**-**b** Dot plot (**a**) and histograms (**b**) showing transport displacement of *MS2 only* or *MS2+Rgs4* 3´-UTR reporter mRNAs, detected by tdMCP-GFP in co-transfected rat hippocampal neurons. **c** Representative kymographs of GFP fluorescence of hippocampal neuronal culture co-transfected with the *MS2+Rgs4* 3´-UTR reporter and tdMCP-GFP constructs, the later either containing (+) or lacking (-) a nuclear localization signal (NLS). **d** Dot plot displaying percentage of anterograde moving granules for *MS2 only* or *MS2+Rgs4* 3´-UTR reporter mRNAs, detected by tdMCP-GFP lacking a NLS. Data represents mean ± standard deviation of independent experiments (individual experiments shown as gray dots). Data was obtained from 40 µm dendritic segments at a minimal distance of 20 µm from the cell body. Total number of dendrites (n_d) and tracks (n_t) analyzed per condition are indicated. Only displacements ≥ 1.5 µm were considered for analysis. Related to Fig. 2.

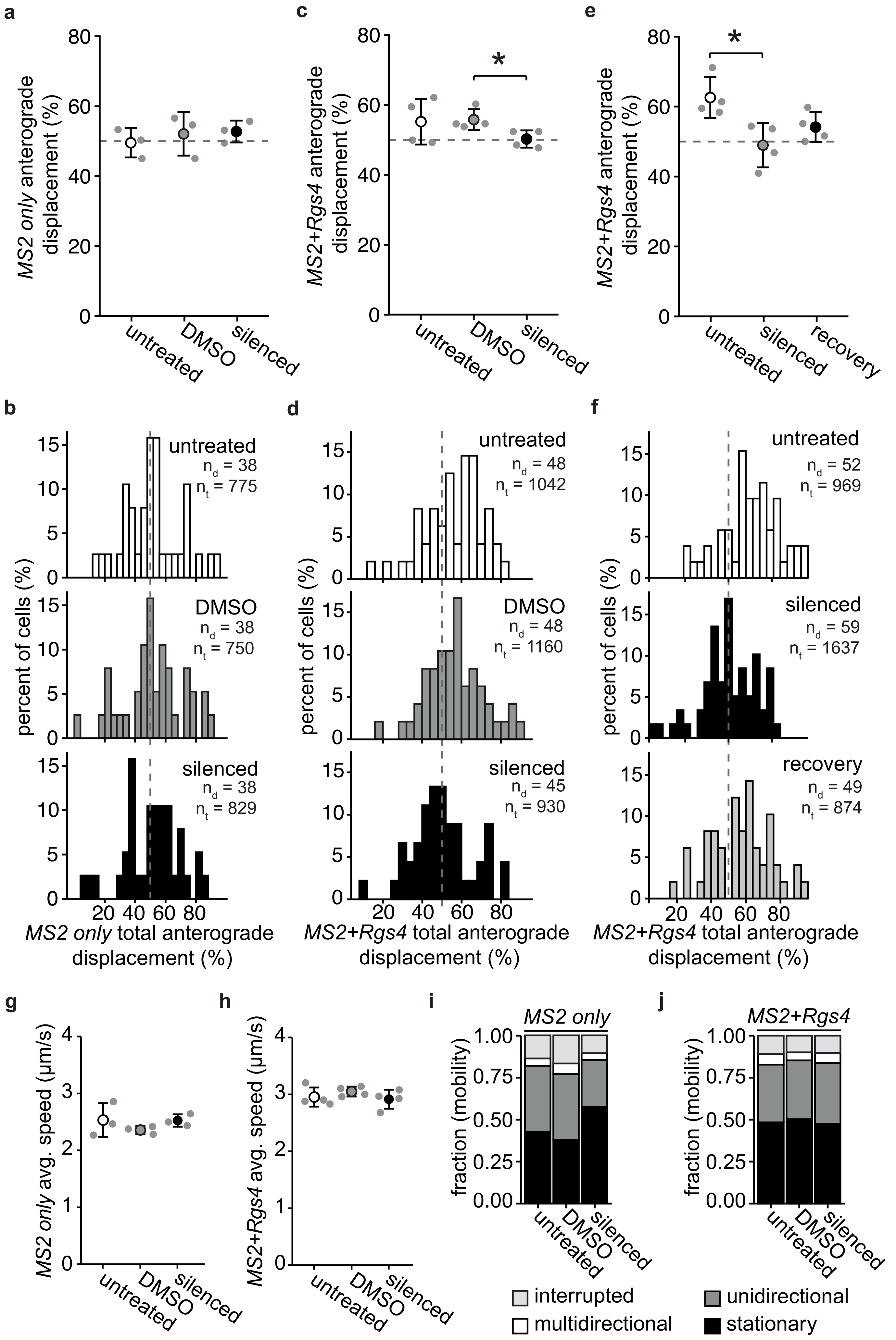

SUPPLEMENTARY FIGURE 3

Supplementary Fig. 3 Chemical inhibition of neuronal activity affects *Rgs4* 3´-UTR dependent total anterograde displacement. **a**-**h** Dot plots (**a**,**c**,**e**,**g**,**h**) and histograms (**b**,**d**,**f**) displaying the percentage of total anterograde displacement (**a**-**f**) and average speed (**g**,**h**) of *MS2 only* (**a**,**b**,**g**) or *MS2+Rgs4* 3´-UTR (**c**-**f**,**h**) reporter mRNAs in rat hippocampal neurons, under untreated, vehicle (DMSO) or silenced (100 µM CNQX, 50 µM AP5, 1 µM TTX) conditions and after recovery for 1 hour. Data represents mean ± standard deviation of 3-4 independent experiments (individual experiments shown as gray dots). Asterisks represent *p*-values assessed by Student's t-test (**c**) or Tukey's test post-hoc to one-way ANOVA analysis (**e**) (* $p < 0.05$). Data was obtained from 40 µm dendritic segments at a minimal distance of 20 µm from the cell body. At least 10 dendrites/condition/experiment were analyzed. Total number of dendrites (n_d) and tracks (n_t) analyzed per condition are indicated. Only displacements ≥ 1.5 µm were considered for analysis. **i**-**j** Quantification of relative transport dynamics of *MS2 only* (**i**, 9-10 dendrites per condition) and *MS2+Rgs4* 3´-UTR (**j**, 35-38 dendrites per condition) reporter mRNAs under untreated, vehicle (DMSO) or silenced (100 µM CNQX, 50 µM AP5, 1 µM TTX) conditions in 1 minute time-series acquisitions. Related to Fig. 3.

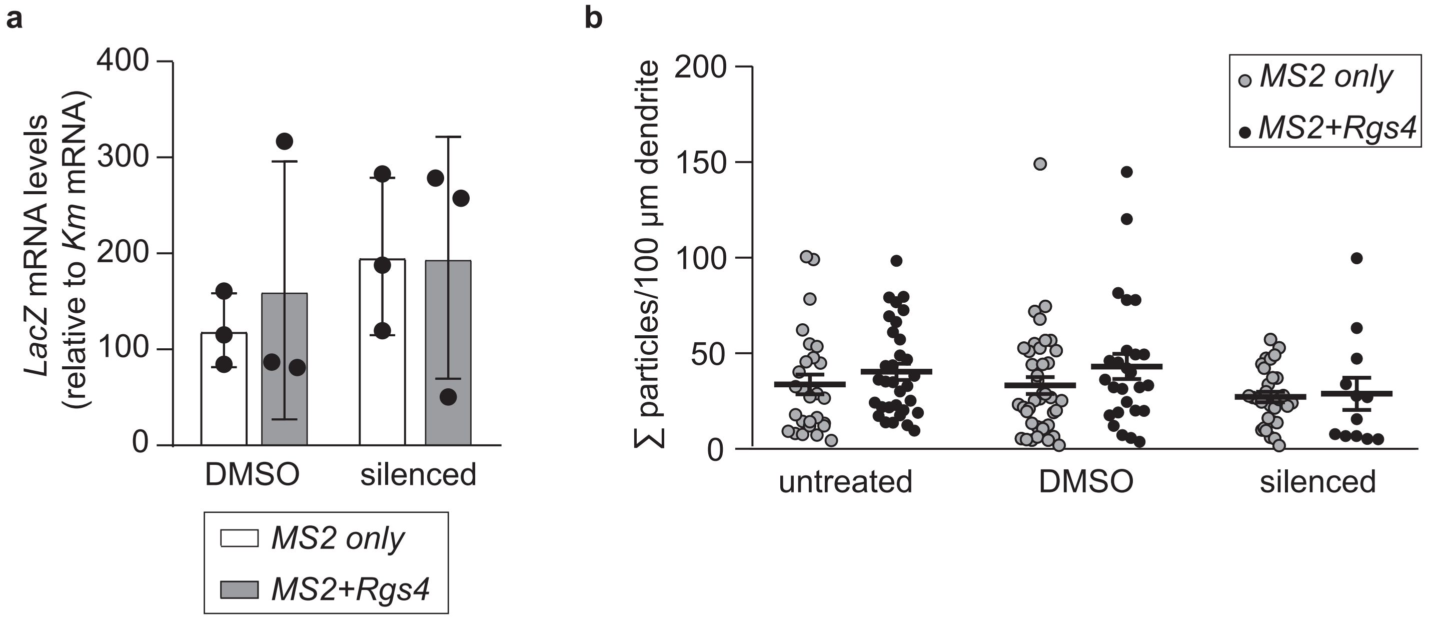

Supplementary Fig. 4 Chemical inhibition of neuronal activity does not affect mRNA expression levels and dendritic mRNA granule number. **a** Bar plot displaying LacZ mRNA reporter levels evaluated by RT-qPCR from 15 DIV cortical neurons transfected with either the MS2 only or MS2+Rgs4 reporters under vehicle treated (DMSO) or silenced (100 µM CNQX, 50 µM AP5, 1 µM TTX) conditions. Data represents mean ± standard deviation of 3 independent experiments (individual experiments shown as black dots). Km = Kanamycin. **b** Bar plot displaying number of dendritic MS2 only or MS2+Rgs4 3´-UTR reporter mRNA granules in rat hippocampal neurons under untreated, vehicle treated (DMSO) or silenced (100 µM CNQX, 50 µM AP5, 1 µM TTX) conditions. Data represents mean ± standard error of the mean of 2 independent experiments (individual dendrites shown as dots, 10-40 dendrites/condition). Related to Fig. 3.

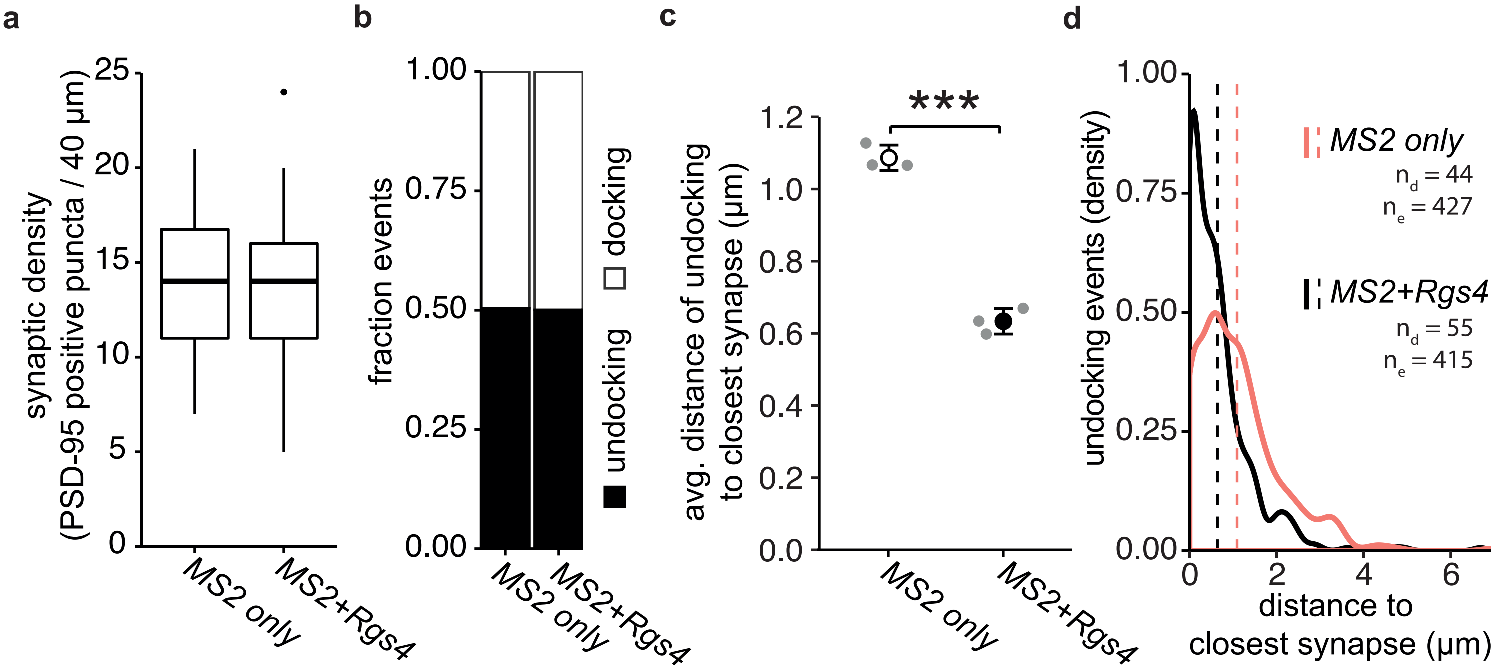

e

	somatic synapses		dendritic synapses	
	dynamic	non-dynamic	dynamic	non-dynamic
MS2 only	112 (76.1%)	35	440 (34.5%)	835
MS2+Rgs4	60 (76.1%), $p = 1$	18	252 (21.2%), $p < 0.0001$	932

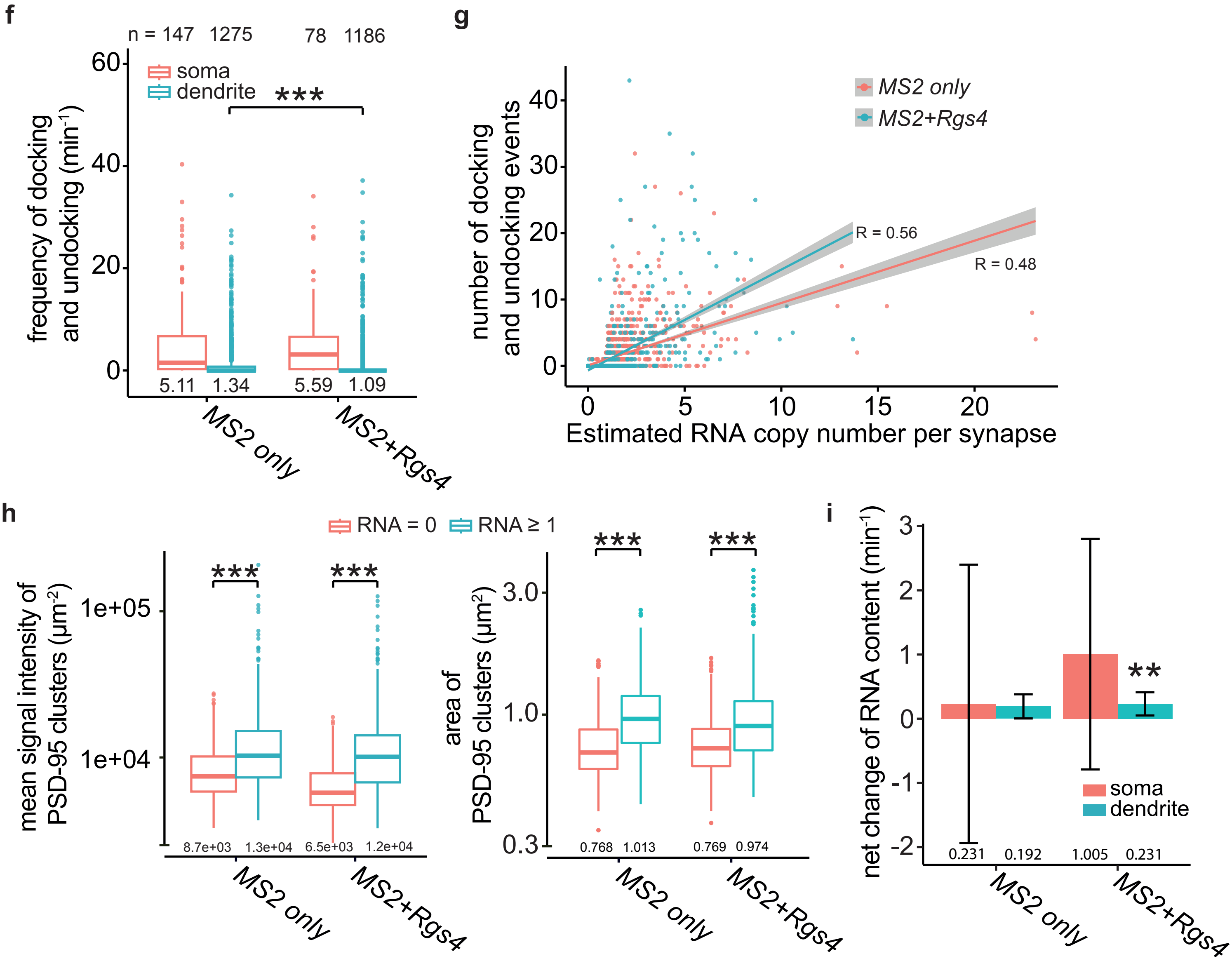

Supplementary Fig. 5 *Rgs4* 3´-UTR mRNA undergoes docking and undocking in the proximity of synapses. **a** Boxplot displaying synaptic density measured as number of PSD-95 positive clusters per 40 µm dendritic segment in hippocampal neurons co-transfected with either *MS2 only* or *MS2+Rgs4* 3´-UTR reporters and PSD-95-tagRFPt. **b** Bar plot showing the fraction of docking and undocking events of *MS2 only* or *MS2+Rgs4* 3´-UTR reporters in hippocampal neurons transfected as in **a**. **c**-**d** Dot plot (**c**) and density plot (**d**) displaying the distance between undocking events and the closest PSD-95 positive cluster in co-transfected rat hippocampal neurons. Data displays both *MS2 only* and *MS2+Rgs4* 3´-UTR reporter mRNAs. Data represents mean ± standard deviation of 3 independent experiments in dot plot (**c**; individual experiments shown as gray dots). Dashed lines represent mean of individual data points (**d**). Data was obtained from 40 µm dendritic segments at a minimal distance of 20 µm from the cell body. Asterisks represent *p*-values obtained by Student's t-test (**c**; *** $p < 0.001$). **e** Distribution of dynamic (at least one docking and/or undocking event of the reporter RNA) and non-dynamic PSD-95-tagRFPt positive clusters located in the soma or in dendrites. *P*-values of Chi^2 tests against the control are indicated. **f** Integrated frequency of the reporter docking and undocking events in somatic and dendritic synapses per minute. Number of observations and the population mean are indicated above and below boxplots, respectively. **g** Integrated number of docking and undocking events as a function of estimated RNA copy number per synapse. Fitted lines and goodness of fit (R) are indicated. **h** PSD-95-tagRFPt signal intensity over synaptic area (left) and PSD-95-tagRFPt positive area (left) in presence or absence of RNA at t = 0s. **i** Average net change of *MS2 only* or *MS2+Rgs4* mRNA content in somatic and dendritic synapses. Numbers indicate the mean value of net RNA level change. Error bars represent the 95% confidence intervals. (** $p < 0.01$; difference compared to zero (no net flux, null

hypothesis)). Asterisks represent *p*-values obtained by Mann Whitney U test (**f**,**h**; *** $p < 0.001$) or two sided one-sample t-test (**i;** ** indicates significant difference compared to zero, $\alpha=0.01$). At least 10 dendrites/condition/experiment (**a**-**d**) or 12 neurons/condition (**e**-**i**) from 3 independent biological replicates were analyzed. Total number of dendrites (n_d), events (n_e) and synapses (n) analyzed per condition are indicated. Related to Fig. 4

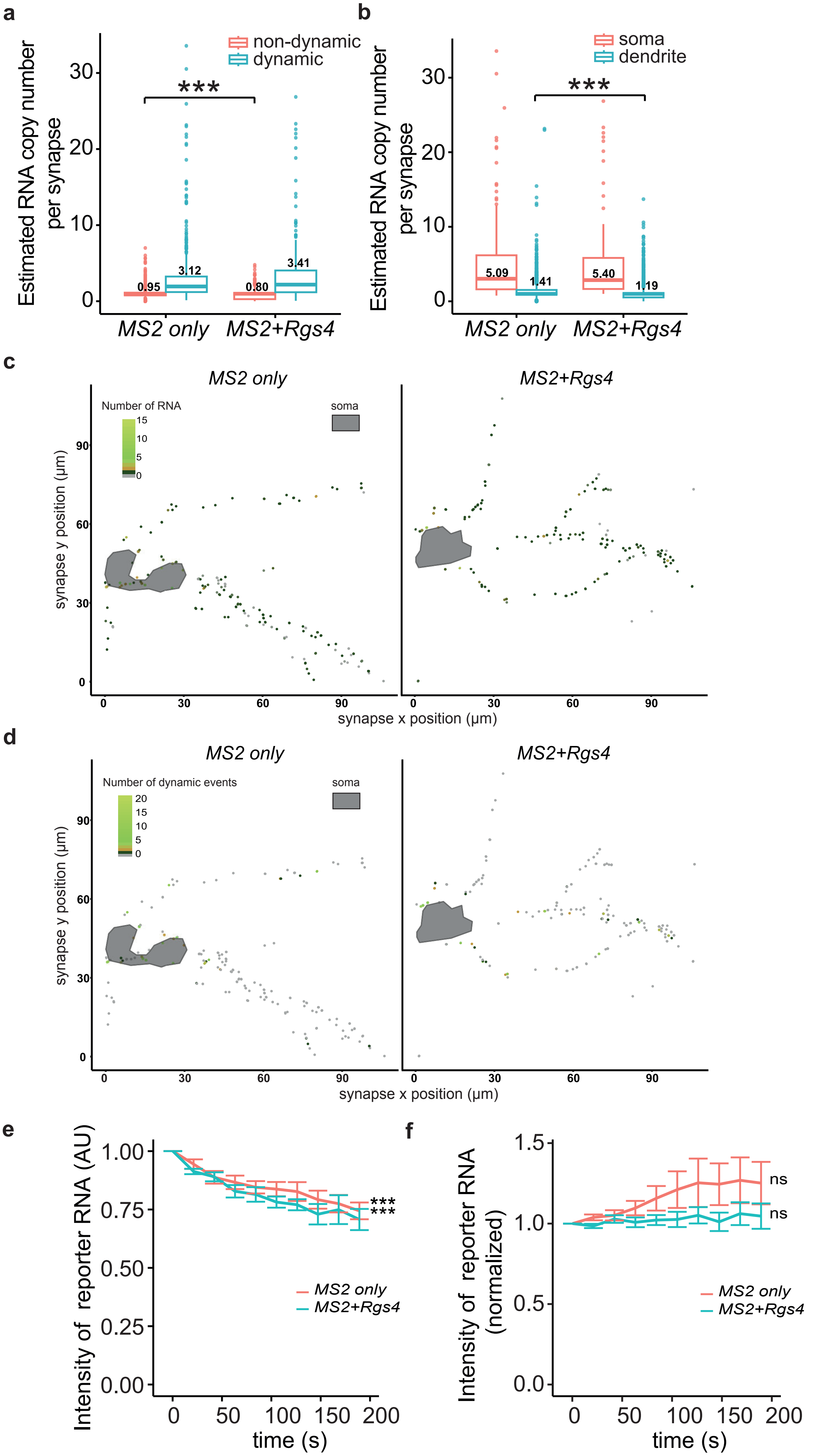

SUPPLEMENTARY FIGURE 6

Supplementary Fig. 6 mRNA copy number in hippocampal neurons and correction of photobleaching. **a**-**b** Estimated *MS2 only* and *MS2+Rgs4* reporter mRNA copy number in dynamic or non-dynamic (**a**) and somatic or dendritic (**b**) synapses. Numbers indicate the mean value of estimated RNA levels. **c**-**d** Representative detection masks of PSD-95-RFP clusters, color-code indicating estimated mRNA content (**c**) or number of docking/undocking events (**d**) with *MS2 only* (left) or *MS2+Rgs4* (right) reporter mRNAs. Gray areas indicate cell soma. **e**-**f** Intensity of reporter RNA (red – *MS2 only*, blue – *MS2+Rgs4*) at synapses normalized to the first frame to correct the effects of photobleaching (**e**). Photobleaching was compensated by adjusting the unit threshold in 100 frame increments (**f**, see also *Methods*). Asterisks represent *p*-values obtained by Mann Whitney U test (*** $p < 0.001$) between different synaptic populations (**a**,**b**) or between the first and last data points of corresponding experiments (**e**); ns indicates non-significant ($p > 0.05$) between the first and last data points of corresponding experiments. 12 neurons/condition from 3 independent biological replicates were analyzed. Related to Fig. 4

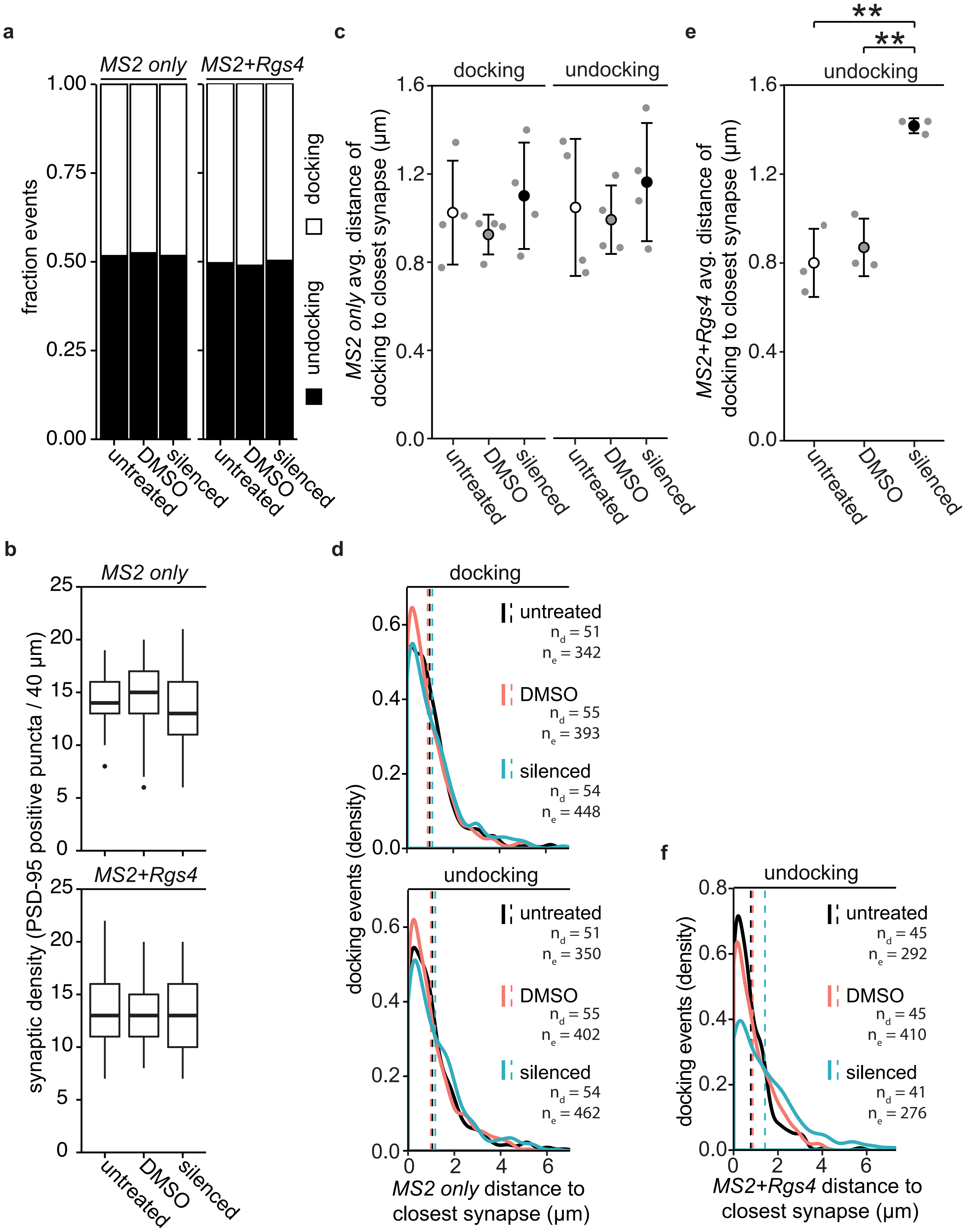

SUPPLEMENTARY FIGURE 7

Supplementary Fig. 7 *Rgs4* 3´-UTR mRNA undergoes docking and undocking in the proximity of synapses, dependent on neuronal activity. **a** Bar plot showing the fraction of docking to undocking events in *MS2 only* or *MS2+Rgs4* 3´-UTR reporter transfected hippocampal neurons under untreated, vehicle (DMSO) or silenced (100 µM CNQX, 50 µM AP5, 1 µM TTX) conditions. **b** Boxplots displaying synaptic density measured as number of PSD-95-tagRFPt positive clusters per 40µm dendritic segment in hippocampal neurons co-transfected with either *MS2 only* (top) or *MS2+Rgs4* 3´-UTR (down) reporters and PSD-95-tagRFPt, treated as in **a**. **c**-**f** Dot plots (**c**,**e**) and density plots (**d**,**f**) displaying the distance between docking (**c**,**d**) or undocking (**c**-**f**) events to the closest PSD-95 positive cluster in co-transfected rat hippocampal neurons with either *MS2 only* (**c**,**d**) or *MS2+Rgs4* 3´-UTR (**e**,**f**) reporters and PSD-95-tagRFPt, under untreated, vehicle (DMSO) and silenced (100 µM CNQX, 50 µM AP5, 1 µM TTX) conditions. Data represents mean ± standard deviation of 3-4 independent experiments in dot plots (**c**,**e**; individual experiments shown as gray dots). Dashed lines represent mean of individual data points (**d**,**f**). Asterisks represent *p*-values obtained by Tukey's test post-hoc to one-way ANOVA analysis (** $p < 0.01$). Data was obtained from 40 µm dendritic segments at a minimal distance of 20 µm from the cell body. At least 10 dendrites/condition/experiment were analyzed. Total number of dendrites (n_d) and events (n_e) analyzed per condition are indicated. Related to Fig. 4.

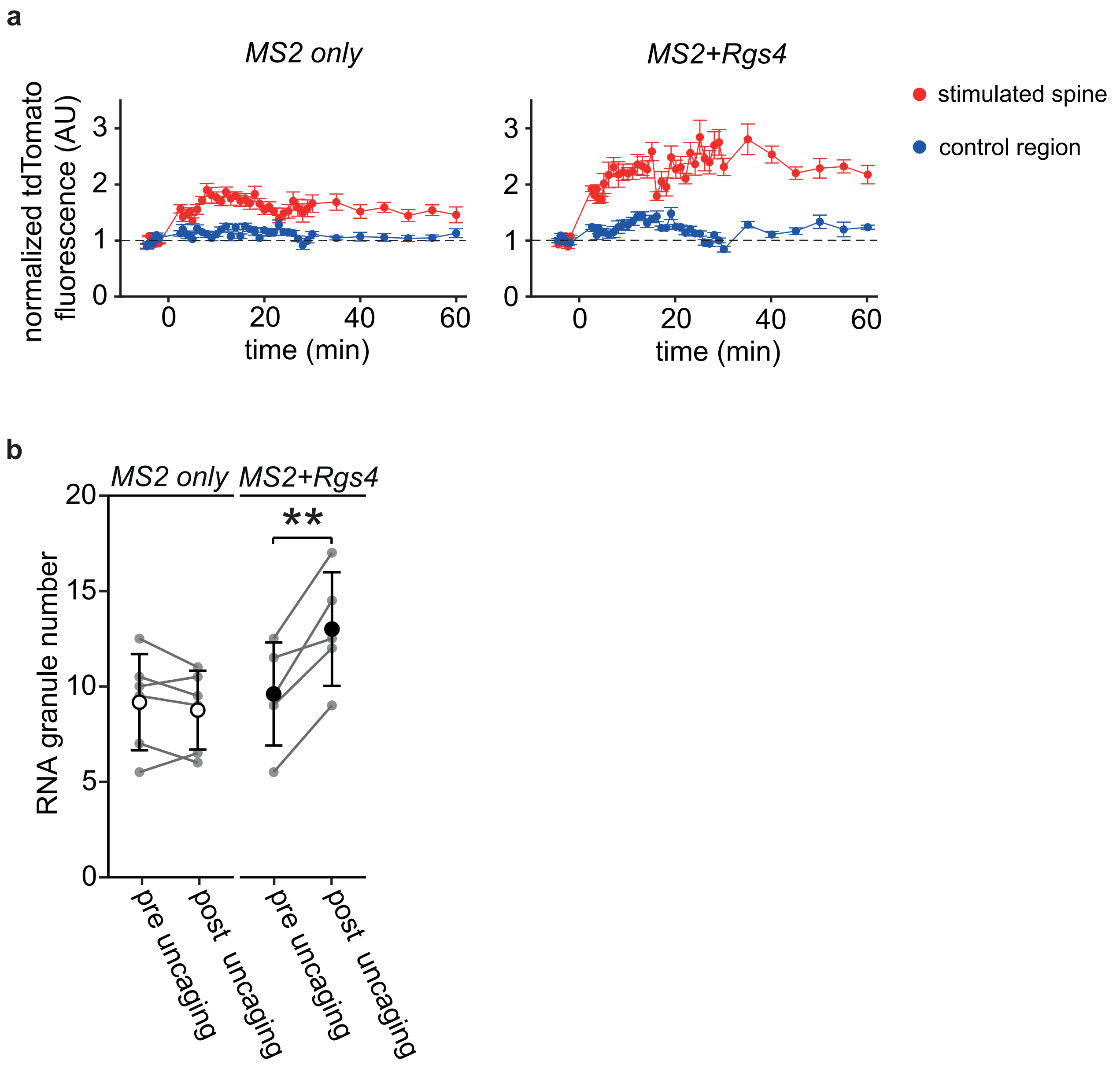
a
MS2 only
MS2+Rgs4
stimulated spine
control region
normalized tdTomato fluorescence (AU)
time (min)
b
MS2 only
MS2+Rgs4
**
RNA granule number
pre uncaging
post uncaging

Supplementary Fig. 8 Local Glutamate uncaging at individual dendritic spines triggers *Rgs4* 3´-UTR dependent mRNA recruitment. **a** Fluorescent intensity of volume marker (tdTomato) in stimulated dendritic spines (red) or control regions (unstimulated spine or dendritic segment, blue) over time, normalized to first measurement, in neurons transfected with tdTomato, tdMCP-GFP and either *MS2 only* or *MS2+Rgs4* reporter mRNAs. Data represents mean ± standard error of the mean. **b** Dot plot displaying the number of RNA granules of *MS2 only* or *MS2+Rgs4* 3´-UTR reporter mRNAs pre (2-7 min before) and post (40-45 min after) uncaging in rat hippocampal neurons within 5 µm of the stimulated spine. Data represents mean ± standard deviation (individual neurons shown as gray dots linked by gray lines). Data was obtained from 6 dendrites (from 5 neurons of 4 biological replicates) and 5 dendrites (from 5 neurons of 5 biological replicates) for *MS2 only* and *MS2+Rgs4* reporter mRNAs, respectively. Asterisks represent *p*-values obtained by paired Student's t-test (** $p < 0.01$). Related to Fig. 5.

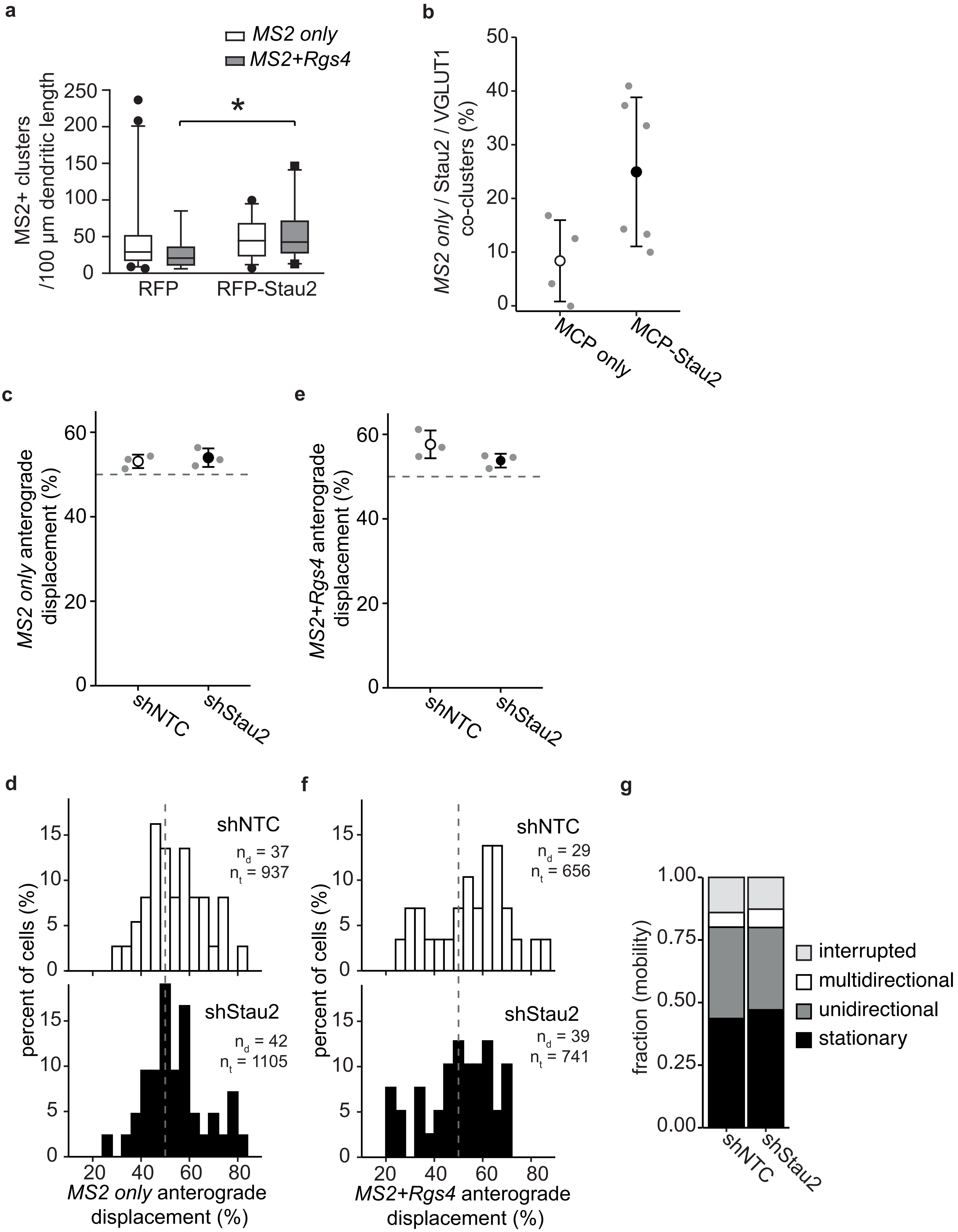

SUPPLEMENTARY FIGURE 9

Supplementary Fig. 9 Effects of Stau2 on *Rgs4* 3´-UTR dependent mRNA localization and transport. **a** Boxplot displaying number of dendritic *MS2 only* or *MS2+Rgs4* 3´-UTR reporter mRNA granules co-transfected with either RFP or RFP-Stau2. Asterisks represent *p*-values obtained by Student's t-test (* $p < 0.05$). **b** Dot plot displaying percent of dendritic *MS2 only* reporter mRNA granules in co-clusters with Stau2 and vesicular glutamate transporter 1 (VGLUT 1), in hippocampal neurons co-transfected with *MS2 only* mRNA, tdMCP-GFP and either tdMCP only or tdMCP-Stau2 and stained with anti-Stau2 and anti-VGLUT 1 antibodies. Data represents mean ± standard deviation (individual neurons shown as gray dots). **c**-**f** Dot plots (**c**,**e**) and histograms (**d**,**f**) displaying the percentage of total anterograde displacement of *MS2 only* or *MS2+Rgs4* 3´-UTR reporter mRNA granules in shNTC and shStau2 transduced hippocampal neurons. NTC = non-targeting control. Data represents mean ± standard deviation of three independent experiments (individual experiments shown as gray dots). Data was obtained from 40 µm dendritic segments at a minimal distance of 20 µm from the cell body. Only displacements ≥ 1.5 µm were considered for analysis. **g** Quantification of relative transport dynamics of *MS2+Rgs4* 3´-UTR reporter mRNA in shNTC and or shStau2 transduced neurons, in 1 minute time-series acquisitions. Related to Fig. 6.

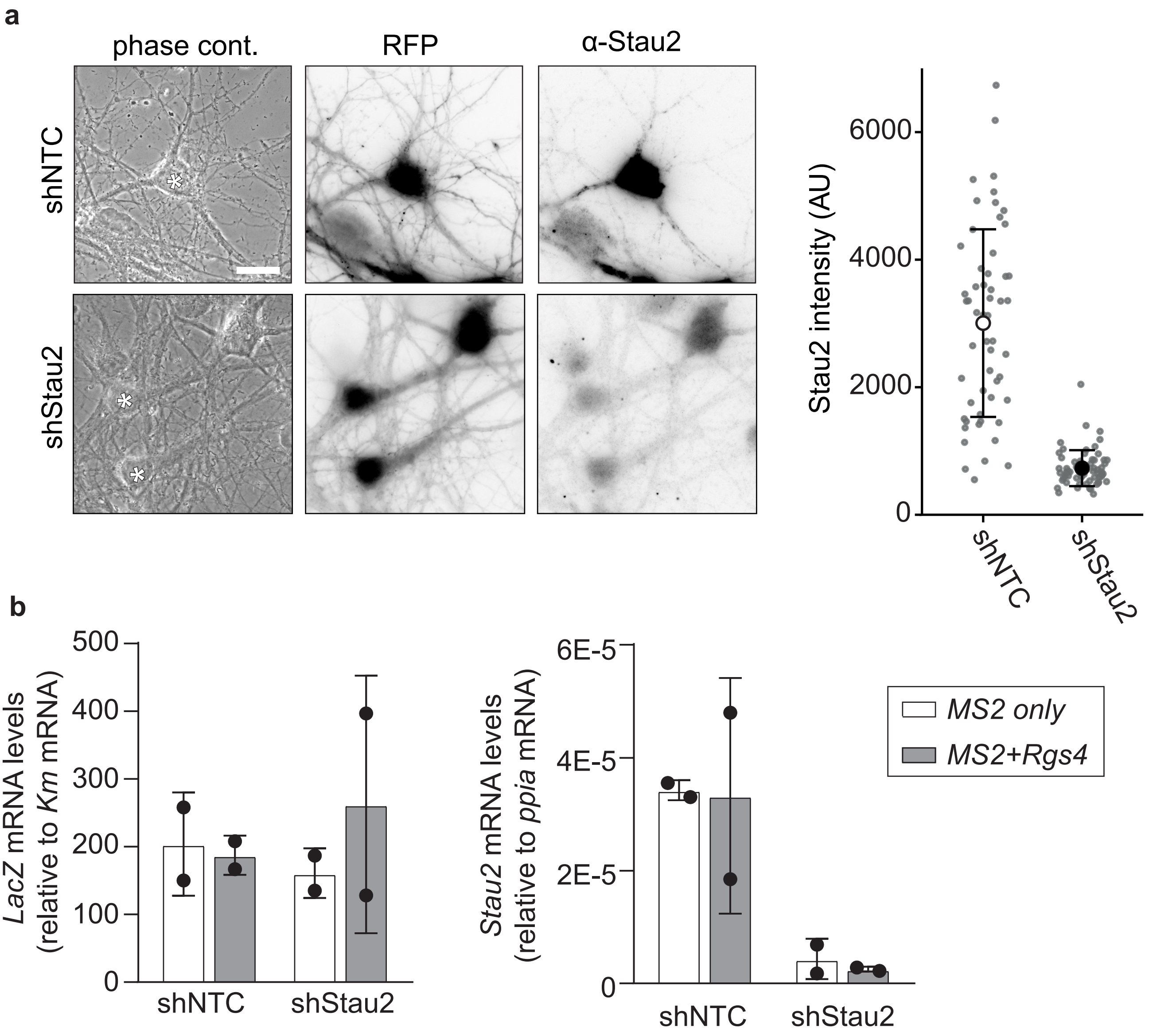

Supplementary Fig. 10 Stau2 knock-down efficiency in hippocampal and cortical neurons in culture. **a** Representative hippocampal neurons transduced with either shNTC or shStau2 lentiviral particles (RFP) and transfected with MS2+Rgs4 3´-UTR reporter and MCP-GFP, were fixed after imaging and stained with anti-Stau2 antibodies (left). Stau2 intensity quantification (right) in rat hippocampal neurons 5 days after viral transduction with either shNTC or shStau2 lentiviral particles. Asterisks denote MCP-GFP positive cells. Scale bar 10 µm. **b** Bar plot displaying LacZ reporter (left) or endogenous Stau2 (right) mRNA levels evaluated by RT-qPCR from 15 DIV cortical neurons transduced with either shNTC or shStau2 lentiviral particles and transfected with either the MS2 only or MS2+Rgs4 3´-UTR reporters. Data represents mean ± standard deviation of 2 independent experiments (individual experiments shown as black dots). Km = Kanamycin; ppia = peptidylprolyl isomerase A. Related to Fig. 6.
