# Supplementary material for: Live cell imaging reveals 3′-UTR dependent mRNA sorting to synapses
Source: Nat Commun. 2019 Jul 18;10:3178. doi: 10.1038/s41467-019-11123-x (PMC6639396; doi:10.1038/s41467-019-11123-x)
Supplement: Supplementary file 3 — Reporting Summary [file 41467_2019_11123_MOESM3_ESM.pdf]

nature research

Corresponding author(s): Michael A Kiebler

Last updated by author(s): Jun 13, 2019

Reporting Summary

Nature Research wishes to improve the reproducibility of the work that we publish. This form provides structure for consistency and transparency in reporting. For further information on Nature Research policies, see Authors & Referees and the Editorial Policy Checklist.

Statistics

For all statistical analyses, confirm that the following items are present in the figure legend, table legend, main text, or Methods section.

n/a	Confirmed	
☐	☒	The exact sample size (n) for each experimental group/condition, given as a discrete number and unit of measurement
☐	☒	A statement on whether measurements were taken from distinct samples or whether the same sample was measured repeatedly
☐	☒	The statistical test(s) used AND whether they are one- or two-sided<br>*Only common tests should be described solely by name; describe more complex techniques in the Methods section.*
☒	☐	A description of all covariates tested
☐	☒	A description of any assumptions or corrections, such as tests of normality and adjustment for multiple comparisons
☐	☒	A full description of the statistical parameters including central tendency (e.g. means) or other basic estimates (e.g. regression coefficient) AND variation (e.g. standard deviation) or associated estimates of uncertainty (e.g. confidence intervals)
☐	☒	For null hypothesis testing, the test statistic (e.g. F, t, r) with confidence intervals, effect sizes, degrees of freedom and P value noted<br>*Give P values as exact values whenever suitable.*
☒	☐	For Bayesian analysis, information on the choice of priors and Markov chain Monte Carlo settings
☒	☐	For hierarchical and complex designs, identification of the appropriate level for tests and full reporting of outcomes
☒	☐	Estimates of effect sizes (e.g. Cohen's d, Pearson's r), indicating how they were calculated

Our web collection on statistics for biologists contains articles on many of the points above.

Software and code

Policy information about availability of computer code

Data collection	n/a
Data analysis	n/a

For manuscripts utilizing custom algorithms or software that are central to the research but not yet described in published literature, software must be made available to editors/reviewers. We strongly encourage code deposition in a community repository (e.g. GitHub). See the Nature Research guidelines for submitting code & software for further information.

Data

Policy information about availability of data

All manuscripts must include a data availability statement. This statement should provide the following information, where applicable:

- Accession codes, unique identifiers, or web links for publicly available datasets
- A list of figures that have associated raw data
- A description of any restrictions on data availability

Provide your data availability statement here.

Field-specific reporting

Please select the one below that is the best fit for your research. If you are not sure, read the appropriate sections before making your selection.

☒ Life sciences ☐ Behavioural & social sciences ☐ Ecological, evolutionary & environmental sciences

For a reference copy of the document with all sections, see nature.com/documents/nr-reporting-summary-flat.pdf

Life sciences study design

All studies must disclose on these points even when the disclosure is negative.

Sample size	For most experiments, at least 10 dendrites/condition/experiment were analyzed. 3-4 Experiments were conducted, according to standard scientific practice.<br>For analysis of single synapses, 10 neurons/condition; >1000 synapses/condition were analyzed.<br>For RT qPCR 2-3 experiments were conducted with 2-3 technical replicates each.
Data exclusions	n/a
Replication	In most cases 3-4 biological replicates were performed.
Randomization	n/a
Blinding	Images and time-lapse movies were acquired and analyzed blindly for experimental condition.

Reporting for specific materials, systems and methods

We require information from authors about some types of materials, experimental systems and methods used in many studies. Here, indicate whether each material, system or method listed is relevant to your study. If you are not sure if a list item applies to your research, read the appropriate section before selecting a response.

Materials & experimental systems

n/a	Involved in the study	
☐	☒	Antibodies
☐	☒	Eukaryotic cell lines
☒	☐	Palaeontology
☐	☒	Animals and other organisms
☒	☐	Human research participants
☒	☐	Clinical data

Methods

n/a	Involved in the study	
☒	☐	ChIP-seq
☒	☐	Flow cytometry
☒	☐	MRI-based neuroimaging

Antibodies

Antibodies used	polyclonal rabbit anti-Staufen2, guinea pig anti-VGLUT1 (Synaptic Systems, 419005)
Validation	Goetze, B. et al. The brain-specific double-stranded RNA-binding protein Staufen2 is required for dendritic spine morphogenesis. J Cell Biol 172, 221-231 (2006).<br>Fritzsche, R. et al. Interactome of two diverse RNA granules links mRNA localization to translational repression in neurons. Cell Rep 5, 1749-1762 (2013)<br>This study (Supplementary Figure 10)

Eukaryotic cell lines

Policy information about cell lines

Cell line source(s)	primary rat hippocampal and cortical neurons, AAV293 (Agilent)
Authentication	in house isolation, AAV293 #240073 Agilent
Mycoplasma contamination	n/a
Commonly misidentified lines (See ICLAC register)	n/a

Animals and other organisms

Policy information about studies involving animals; ARRIVE guidelines recommended for reporting animal research

Laboratory animals	Embryonic day 17 (E17) embryos of timed pregnant Sprague-Dawley rats (Charles River Laboratories)
Wild animals	n/a

Field-collected samples	n/a
Ethics oversight	All animals were used according to the German Welfare for Experimental Animals (LMU-Munich, Regierung von Oberbayern).

Note that full information on the approval of the study protocol must also be provided in the manuscript.
