# Supplementary material for: Live cell imaging reveals 3′-UTR dependent mRNA sorting to synapses
Source: Nat Commun. 2019 Jul 18;10:3178. doi: 10.1038/s41467-019-11123-x (PMC6639396; doi:10.1038/s41467-019-11123-x)
Supplement: Supplementary file 4 — Description of Additional Supplementary Files [file 41467_2019_11123_MOESM4_ESM.docx]

**Description of Additional Supplementary Files**

File Name: Supplementary Movie 1
Description: Representative time-lapse movie of *MS2+Rgs4* 3´-UTR reporter mRNA granules moving at different speed (arrowheads) in a dendrite of a 15 DIV hippocampal neuron. Green arrowheads indicate anterograde movement. Playback speed 2x real time. Scale bar 10 µm. Related to Fig. 1C.

File Name: Supplementary Movie 2

Description: Representative time-lapse movie of *MS2+Rgs4* 3´-UTR reporter mRNA granules moving different displacement lengths (arrowheads) in a dendrite of a 14 DIV hippocampal neuron. Red arrowheads indicate retrograde movement. Playback speed 2x real time. Scale bar 10 µm. Related to Fig. 1D.

File Name: Supplementary Movie 3

Description: Representative time-lapse movie of *MS2+Rgs4* 3´-UTR reporter mRNA granules moving in different directions (arrowheads) in a dendrite of a 15 DIV hippocampal neuron. Green arrowhead indicates anterograde movement, red arrowhead indicates retrograde movement. Playback speed 2x real time. Scale bar 10 µm. Related to Fig. 1E.

File Name: Supplementary Movie 4

Description: Representative time-lapse movie of an *MS2+Rgs4* 3´-UTR reporter mRNA granule interrupting movement (arrowhead) in a dendrite of a 15 DIV hippocampal neuron. Playback speed 2x real time. Scale bar 10 µm. Related to Fig. 1F.

File Name: Supplementary Movie 5

Description: Representative time-lapse movie of an *MS2+Rgs4* 3´-UTR reporter mRNA granule moving in an uninterrupted multidirectional fashion (arrowhead) in a dendrite of a 14 DIV hippocampal neuron. Playback speed 2x real time. Scale bar 10 µm. Related to Fig. 1G.

File Name: Supplementary Movie 6

Description: Representative time-lapse movie of an *MS2+Rgs4* 3´-UTR reporter mRNA granule moving in an uninterrupted multidirectional fashion (arrowhead) in a dendrite of a 14 DIV hippocampal neuron. Playback speed 4x real time. Scale bar 10 µm. Related to Fig. 1.

File Name: Supplementary Movie 7

Description: Representative time-lapse movie of an *MS2+Rgs4* 3´-UTR reporter mRNA granule moving in a multidirectional fashion between dendrites at a branching point (arrowhead) in a 12 DIV hippocampal neuron. Playback speed 2x real time. Scale bar 10 µm. Related to Fig. 1.

File Name: Supplementary Movie 8

Description: Representative time-lapse movie of *MS2+Rgs4* 3´-UTR reporter mRNA granules moving in different directions (arrowheads) in a dendrite of a 14 DIV hippocampal neuron. Green arrowhead indicates anterograde movement, red arrowheads indicate retrograde movement. Playback speed 2x real time. Scale bar 10 µm. Related to Fig. 2A-C.

File Name: Supplementary Movie 9

Description: Representative time-lapse movie of an *MS2+Rgs4* 3´-UTR reporter mRNA granule (green fluorescence and arrowhead) moving to a PSD-95-TagRFPt positive cluster (magenta fluorescence and arrowhead) in a dendrite of a 15 DIV hippocampal neuron. Playback speed 2x real time. Scale bar 10 µm. Related to Fig. 4A.

File Name: Supplementary Movie 10

Description: Representative time-lapse movie of an *MS2+Rgs4* 3´-UTR reporter mRNA granule (green fluorescence and arrowhead) moving to a PSD-95-TagRFPt positive cluster (magenta fluorescence and arrowhead) in a dendrite of a 15 DIV hippocampal neuron. Playback speed 2x real time. Scale bar 10 µm. Related to Fig. 4.

File Name: Supplementary Movie 11

Description: Representative time-lapse movie of *MS2+Rgs4* 3´-UTR reporter mRNA granules in a dendrite of an 18 DIV hippocampal neuron upon glutamate uncaging. Green arrowheads indicate examples of GFP positive *MS2+Rgs4* 3´-UTR reporter mRNA granules, red dot indicates uncaging spot. Playback pre uncaging indicated by negative time, playback post uncaging indicated by positive time. Playback speed variable (increased over time). Scale bar 5 µm. Related to Fig. 5.

File Name: Supplementary Movie 12

Description: Representative time-lapse movie of an *MS2+Rgs4* 3´-UTR reporter mRNA (green fluorescence and arrowhead) and TagRFPt-Stau2 (magenta fluorescence and arrowhead) positive RNA granule undergoing co-transport in a dendrite of a 15 DIV hippocampal neuron. Playback speed 2x real time. Scale bar 10 µm. Related to Fig. 6A.

File Name: Supplementary Movie 13

Description: Representative time-lapse movie of an *MS2+Rgs4* 3´-UTR reporter mRNA (green fluorescence and arrowhead) and TagRFPt-Stau2 (magenta fluorescence and arrowhead) positive RNA granule undergoing co-transport in a dendrite of a 15 DIV hippocampal neuron. Playback speed 2x real time. Scale bar 10 µm. Related to Fig. 6.
