# Supplementary material for: Live cell imaging reveals 3′-UTR dependent mRNA sorting to synapses
Source: Nat Commun. 2019 Jul 18;10:3178. doi: 10.1038/s41467-019-11123-x (PMC6639396; doi:10.1038/s41467-019-11123-x)
Supplement: Supplementary file 2 — Peer Review [file 41467_2019_11123_MOESM2_ESM.pdf]

Reviewers' comments:

Reviewer #1 (Remarks to the Author):

The manuscript by Bauer et al. describes the 3′UTR-dependent transport of Rgs4 mRNA in live neurons. Using the MS2-GFP system, the authors studied the role of the 3′UTR of Rgs4 in the biased transport of RNA granules in neurons. They found that bidirectional transport of RNA in dendrites was independent of the presence of the Rgs4 3′UTR. However, the Rgs4 3′UTR caused (1) a biased transport to the anterograde direction and (2) sustainable recruitment to the synapses. Either shRNA knock-down of Staufen2 or chemical inhibition of synaptic activity abolished these effects of Rgs4 3′UTR.
The study is well-conducted to determine the role of the 3′UTR of Rgs4 mRNA in RNA trafficking in dendrites. However, the novelty of their finding is not very clear from the manuscript. The MS2-GFP system has been used to investigate the trafficking of various reporter mRNAs in neurons (some of the references are listed in my comment #1). Most of the results in the manuscript seem to be consistent with the previous findings in the literature on mRNA transport, although this is the first time to investigate Rgs4 mRNA trafficking.
Nonetheless, the authors performed a thorough analysis on the role of Staufen2 and neuronal activity for the trafficking of Rgs4 mRNA. The analysis schemes used in this study will be useful for the community to compare the effect of 3′UTR or other cis-regulatory elements in many dendritically localized mRNAs. To strengthen the manuscript, I suggest that the authors address a few points described below.

1. In the discussion section, the authors need to compare their results with previous reports on other 3′UTRs labeled with the MS2 system. In addition to the three references (33, 34, 37), there are several more studies using the MS2-labeled mRNA. Here are some examples:
Rook et al., Journal of Neuroscience, 2000
Dynes et al., Journal of Comparative Neurology, 2007
Kao et al., PNAS, 2010
Dictenberg et al., Developmental Cell, 2008
Meer et al., PNAS, 2012

2. Please provide some discussion on the possible molecular mechanism of the Staufen2-mediated biased mRNA transport. Also, more discussion on the known function of Staufen2 in neurons would be informative. For instance, these papers seem to be highly relevant to this study.
Lebeau et al., Staufen 2 regulates mGluR long-term depression and Map1b mRNA distribution in hippocampal neurons. Learning & Memory, 2011
Goetze et al., The brain-specific double-stranded RNA-binding protein Staufen2 is required for dendritic spine morphogenesis. J Cell Biol, 2006

3. In page 16 line 369: there is another recently published computational study related to the sushi-belt model.
Song et al., Neuronal Messenger Ribonucleoprotein Transport Follows an Aging Lévy Walk. Nature communications, 2018

4. In Figure 2D,F,H, Figure 3B,D,F, Figure 4B,G, Figure 5A,C, and other similar figures in the supplementary data, there seem to be only 3 data points, which was alarming to me at first. Only after a careful reading of the figure legends, I understand that these are 3 independent experimental data. To avoid such a misunderstanding, I suggest the authors change these figures as simple bar graphs. Also, it would be informative to provide the number of the mRNAs counted.

5. Figure 1C, D, and E are better to be moved to the supplementary information. The texts “speed”, “displacement”, and “directionality” are not necessary in these figure panels.

Minor comments:
6. The length of the 3′UTRs in Rgs4 and histone-3.3 mRNAs need to be provided in the manuscript.

7. In page 9, what is F_2,9? It needs to be defined in the manuscript.

8. Supplementary Figure 1B seems to be a control experiment with GFP rather than tdMCP-GFP because no puncta are visible in the middle panel. This needs to be explained in the figure legend.

9. The movies need to show the unit of time.

10. 3′UTR needs to be corrected to 3′(prime)UTR.

Reviewer #2 (Remarks to the Author):

The paper provides insights into mRNA sorting mechanisms in dendrites of rodent hippocampal neurons. Dynamics of Rgs4 mRNA movement and synaptic docking are analyzed in detail using MS2-based RNA tagging and high spatiotemporal imaging. There is a small, statistically significant anterograde bias to mRNA transport when the Rgs4 3UTR and Stau2 protein are present. This bias, as well as 3UTR stimulated docking of RNA near synapses, is promoted by neuronal activity.

General evaluation:
This is a high quality study and I have no substantial scientific issues with the presented data or associated interpretations. However, the overall extent to which the data advance our understanding of neuronal mRNA trafficking is diminished by previously published work on the dynamics of mRNAs in hippocampal neurons. Reference 11 in the manuscript describes detailed evaluation of Beta-actin mRNA docking and translation at synapses using RNA tagging and neuronal stimulation, and also includes some analysis of mRNA transport (although does not address directionality). Furthermore, reference 37, as well as two papers by Dynes and Steward (J Comp Neurol. 2007 Jan 20;500(3):433-47 and J Comp Neurol. 2012 Oct 1;520(14):3105-19) demonstrate that there is an anterograde bias in the transport of tagged Arc mRNAs, though this is not activity dependent. Despite this, the work by Bauer et al. is a significant advance as the 3UTR dependence of mRNA dynamics is revealed, Stau2 is identified as a regulator of directionality, and it is shown that hippocampal neurons employ activity-dependent and -independent mRNA sorting mechanisms. Nonetheless, some of the key conclusions overlap with those in published studies (anterograde bias of dendrite localized RNAs and RNA docking at synapses stimulated by activity).

Specific criticisms:
1. The authors truncate the y-axis in many figure panels, which risks misleading readers on the magnitude of effects. Full y-axes should be shown throughout.
2. The sections on the analysis of RNA docking and undocking on pages 11 and 12 are hard to follow. For example, the following is not clear: "In general, synapses contained RNA both at the beginning and during the experiment regardless of their location and the type of the reporter mRNA (Supplementary Fig. 4K-M), indicating that there has to be a net influx of RNA into PSD-95 positive structures". A simplification of the sections on pages 11 and 12, or more intermediate interpretations/conclusions, is needed to clarify this important part of the paper. Why is the number of RNA-associated synapses lower with MS2+Rsg4 3UTR than MS2 alone given there is an anterograde bias to transport and increased docking rate induced by the 3UTR? Is RNA abundance throughout the cell similar for these two mRNAs? If not, it may be appropriate to emphasize normalized data (eg. rate of RNA appearances/disappearance at synapses).
3. The authors discuss the possibility of photobleaching of the mRNA. Unless the contribution of photobleaching can be quantified, it would be more appropriate to refer to appearance and disappearance of RNA signal at synapses rather than docking and undocking.

4. The relationship between net anterograde transport and increased RNA docking at synapses is not clear. At the very least, more speculation on this subject should be included.
5. Although likely a large amount of work, the paper would be stronger if the role of Stau2 in RNA docking was addressed. These experiments may also indicate if the anterograde transport and RNA docking processes can be uncoupled.
6. The authors should state explicitly in the text whether larger or smaller synapses of brighter or dimmer PSD-95 signal intensity correlate with RNA positive and negative synapses.

Reviewer #3 (Remarks to the Author):

In this study, Michael Kiebler's group investigates transport dynamics of mRNAs in dendrites of cultured pyramidal neurons, using live imaging of exogenous mRNAs which are indirectly tagged using the MS2 system. The authors primarily compare the dynamics of two tagged RNAs, a control that lacks a 3'UTR (or ones that include the 3'UTR of transcripts that do not localize to dendrites) and a transcript that includes the 3'UTR of Rgs4, known to localize to neuronal dendrites. Using this paradigm, the authors discover that Rgs4 3'UTR mRNAs show a bias in anterograde transport. This phenomenon depends upon neuronal activity, as pharmacological silencing of cultured neurons abolishes this bias. Next the authors focus on mRNA docking at synapses using fluorescently tagged PSD95 together with MS2 live imaging of mRNAs. With this method they observe that Rgs4 3'UTR mRNAs have a higher probability of docking close to synapses. Taking advantage of endogeous activity of cultured neurons, they show this docking mechanism is mediated by neuronal activity. Finally, the authors employ shRNAs targeting Stau2, which encodes an RNA-binding protein known to bind the Rgs4 3' UTR, to discover that the anterograde bias described above relies on Staufen.
This study is well written and relies on high-quality data. It provides valuable insights into the biological mechanisms by which mRNA transport to dendrites may be coordinated with neuronal activity, and how it could impact activity-related synapse remodeling. With additional controls and some additional experiments, this study will be of impact for the field of mRNA localization in neurons.

1) While the activity- and STAU2-dependent bias towards anterograde movement is a fascinating finding, the significance of this finding for mRNA localization at synapses is a bit unclear. According to the "sushi-belt" hypothesis, the availability of mRNAs close to synapses would be improved without this bias. As noted by the authors, Yoon et al have demonstrated using b-actin reporters that synaptic activity induces transport, translation, and docking close to synapses (Yoon et al, PNAS, 2016). In light of this study, the authors should better clarify the significance and novelty of their findings.

2) The main conclusions of this study rely on overexpression of 2 MS2-tagged constructs. However a major caveat to their interpretations is the levels of expression of these exogenous constructs, Rgs4 3' UTR v MS2 control, which could be vary due to differential transcription or stability. This caveat must be addressed. Perhaps one approach would be to introduce mutation(s) into the 3'UTR for binding sites for Staufen, and compare these to the full length 3' UTR. The authors previously identified Stau2 binding sites on Rgs4. This would also link the 3'UTR analyses to Staufen 2 knockdown, which itself could be indirect.

3) Related to this, to visualize mRNAs transport in dendrites, the authors utilize a version of the MS2 system which relies on the use of tdMCP-EGFP that includes a nuclear localization sequence (NLS) to decrease background noise in the cytoplasm. NLS sequences have been shown to promote microtubule binding and transport of proteins (Salman et al, Biophysical Journal, 2005). The presence of the NLS could thus potentially lead to non-physiological results. This may be especially true given the high number of MS2 loops that the authors used in their constructs. To address this concern, the authors could employ a different mRNA-tagging paradigm that relies on MS2/PP7 split-FPs. The

authors wouldn't need to redo all experiments but just provide evidence that some aspects of mRNA transport are phenocopied (anterograde movement bias OR preferential docking close to synapses).

4) The authors show that neuronal silencing affects anterograde movement and docking, and use endogenous activity in cultures and washout studies to implicate activity in promoting the movement. This point would be better supported if the authors were to stimulate activity and show how responsive (temporally and also qualitatively) the RNAs are to targeting? (For example using glutamate uncaging of hippocampal neurons, as recently used to induce RNA localization to synapses by Yoon et al, 2016). Further, with the current data, there is incomplete rescue of anterograde movements in Figure 3. If they waited longer to do analysis following washout, would the authors see more complete recovery or do they speculate some cells are not responsive?

Minor concerns
1) The authors do not define in the methods or main text how long the 3' UTR is for Rgs4.

2) Please define sushi-belt in introduction, when first mentioning it. Alternatively, please remove.

3) The authors should show graphs reporting both speed and fraction of RNAs moving, when activity is blocked for both MS2 and Rgs4 constructs in Figure 3.

4) The authors should include a control to show that overexpression of tagged PSD95 mimics endogenous synapses in fixed cells as it could potentilaly produce ectopic synapses.

5) The role of STAU2 in transport is interesting. Does STAU2 also regulate preferential docking close to synapses? If tagged would STAU2 move with RNAs? It could be valuable to flesh this part of the story out more to establish a direct relationship, as pointed out in point 1 above.

6) For a better assessment of the quality of the data, the authors should better define the nature of their biological replicates? How many neurons where analyzed for each replicates? How many movements?

7) In two instances, it would be very valuable to merge graphs together and adjust statistical analyses accordingly. This would provide a more powerful way to interpret results. This should be done for Figure 3: B,D,F and Figure 5:A,C.

Response to the reviewers

Please allow us – before we provide a detailed point-by-point response to the individual reviewers – to address a general issue that is relevant to all. Overall, it was suggested that "the authors should better clarify the significance and novelty of their findings." In line with this general point comes the question from two reviewers (Ref. #2 minor point 5 and Ref. #3, point 4), whether we could potentially provide additional experiments to investigate whether Stau2 and/or neuronal activity contribute to the observed recruitment of *Rgs4* mRNA to synapses in more detail.

We appreciate the chance to clarify the significance and the novelty of our study. Of course, our study is not the first to investigate the trafficking of a targeted mRNA in living neurons. A key breakthrough in the field was the introduction of the MS2 RNA imaging system by Ed Bertrand and Rob Singer for *ASH1* mRNA (Ref. #34, Mol Cell 1998, cited). Since then, a series of studies have investigated trafficking of other mRNAs, such as *β-actin* (Ref. #13, Yoon & Singer, 2016, *PNAS;* Ref. #37*,* Park*, 2014*), *CaMKIIα* (Ref. 15, Dictenberg 2008, *Dev Cell*), *Arc* (Refs. #9,14, Dynes & Steward 2007, 2012, Ref. #45, Das & Park, 2018, *Sci. Adv.*)*, sensorin* (Ref. #22, Meer & Martin, 2012 in fixed cells by FISH), and *Drosophila oskar* mRNAs (Ref. #41, Zimyanin & St Johnston, 2008, *Cell*). Of course, we are more than happy to include the additional suggested references (see below) in their context within our manuscript. Few studies (*e.g.* Ref. #37, Park, 2014; Ref. #9, Dynes & Steward, 2007; Ref. #45, Das & Park, 2018) have actually performed sophisticated time-lapse videomicroscopy involving high spatial and temporal resolution. This is, however, very important since it allows to track individual mRNA particles and **to identify various modes of trafficking behavior,** as reported in **Figures 1**, **2, S3 and S5** of our manuscript (see **movies 1-8**). As correctly pointed out by the reviewers, others have also reported a transport bias (we had already cited Ref. 41, Zimyanin & St Johnston, 2008, *Cell*, in the *Drosophila* oocyte as well as Ref. #37, Park 2014, *Science* and Ref. #45, Das & Park, 2018, *Sci. Adv.*). In our revised manuscript, we are now explicitly citing the two studies by Steward (Refs. #9,14, 2007, 2012), in which an anterograde bias was reported for *Arc* mRNA. We agree with the reviewers that the addition of these citations will help the reader to appreciate what findings have been reported before and in which aspects our live cell study exceeds the current understanding of RNA trafficking in neurons. Overall, we made three main advances in our study.

1- Demonstration of the sushi-belt model: bidirectional movement of single mRNAs and transient localization at synapses

The sushi-belt model was originally hypothesized by us in 2011 (Ref. #6, Doyle & Kiebler, *EMBO J.*), and it stipulates that mRNAs move back and forth along dendrites and stops only transiently near synapses, instead of being irreversibly anchored there. Since then, a computational model has been reported supporting the sushi-belt model (Ref. #44, Williams, 2016, *eLife,* see also an alternative model proposed for RNA transport: Ref. #43, Song et al., *2018, Nature Comm.*). However, experimental proof was lacking and we believe that our study is the first to provide a thorough demonstration of this model. For this, we took advantage of a novel RNA tagging variant containing 32 to 128 MS2 stem-loops, instead of the 6 to 24 used previously. This yields large improvements in signals (see also Tantale et al. 2016, *Nature Comm.*). Thanks to this, we could track mRNAs for up to 10 minutes at high frame rates (>10 images/seconds). This was not possible before and it enabled us to precisely characterize the movements of mRNA particles (including the fainter particles that are highly motile). Previous studies used either a low imaging rate (one image every 5 to 10 seconds; Refs. #9,14, Steward 2007, 2012), short observational periods (1 minute; Ref. # 37, Park 2014; Ref. #45, Das and Park 2018), or both (Ref. #13, Yoon 2016). All these studies provided useful information that recapitulated some aspects of the sushi-belt model. For instance, they occasionally reported single particles with transient stop or bidirectional movements. Bidirectional movements, as opposed to particles showing either separate anterograde or retrograde movements, are important, as they rule out the possibility that there are several populations of particles with different fate. However, the low frame rate/short observation window limited the previous analyses and it was therefore unclear whether these behaviors were the exception or the rule, *even though they are the hallmark of the sushi-belt model*. Here, our time-lapse movies (**sample movies 4-6 and quantifications in Figure 1F**) **unequivocally demonstrate that bi-directional movements and transient stops are the norm in mature neurons**. Indeed, increasing the observation period from 1 to 10 minutes augments the frequency of these movements by three fold, such that 60% of the particles show one or the other.

Another important aspect of our original study was the investigation of synaptic recruitment of mRNAs by dual-color time-lapse video-microcopy. We agree with the reviewers that we were indeed not the first ones to report synaptic localization in neurons (*e.g.* Ref. #13, Yoon, 2016; Refs. #9,14,

Dynes and Steward 2007, 2012; Ref. #15, Dictenberg 2008). The cited work has either reported mRNA puncta mostly at the neck of dendritic spines in fixed neurons or in movies that were indicative of synaptic recruitment. However, due to long-term imaging at the detriment of low frame rates, the authors could not follow mRNA fate in real time. Here, we used the brighter MS2 system together with a fluorescently labeled synaptic marker, *i.e.* PSD95-tagRFPt (**Figure 4**), and we believe that we are the first to perform **dual-color, video-rate microscopy to track live synaptic recruitment of mRNAs**. The resulting high resolution time-lapse movies **showed reversible docking of mRNA granules at PSD95 positive synapses** (**movies 9, 10**), confirming another key property of the sushi-belt model: the transient synaptic recruitment of mRNAs (Ref. #6, Doyle & Kiebler, 2011).

2-The 3'-UTR confers activity-dependent synaptic anchoring and activity-dependent directionality bias

While anterograde transport bias was previously reported for *Arc* mRNAs (Ref. #45, Das & Park, 2018, *Sci. Adv;* Refs. #9, Steward, 2007), the mechanisms involved were not determined. Here, we went on to show that it is the 3'-UTR of the *Rgs4* transcript – together with neuronal activity and Staufen2 – that conveys the observed transport bias. We are not aware of another study that has reported **3'-UTR dependent mRNA directionality in dendrites of mature neurons, and the role of an RBP and synaptic activity in regulating this feature of the 3'-UTR.**

Similarly, we have been able to study the dynamicity of mRNAs localizing at synapses, and how this is altered depending on the somatic/dendritic location, the 3'-UTR of the transcript and a possible preceding association of an mRNA at this particular synapse. By analyzing neurons under basal activity or chemical inhibition, we report that it is the **3'-UTR that confers activity-dependent recruitment at synapses.**

To further substantiate these results, we now performed – as suggested by one of the reviewers – glutamate uncaging using two-photon-microscopy and tracked MS2 particles on their way to a stimulated dendritic spine (**new Fig. 5**). Some were recruited to the base of the spine and eventually entered into the neck or sometimes even into the head. Importantly, our pilot experiments showed that 4 out of 5 glutamate-stimulated spines recruited 3'-UTR-containing *Rgs4* mRNA, but none out of 6 spines appeared to recruit control *MS2-only* mRNA (**new Fig. 5**). As we have cited in our manuscript, a similar approach was taken by Yoon and colleagues, who reported activity dependent *ß-actin* mRNA recruitment to activated areas (Ref. #13, Yoon et al., 2016, *PNAS*). In our study, we not only show that ***Rgs4* mRNA can be recruited to single stimulated spines but also that this process is dependent on its 3'-UTR**. This substantiates our initial findings as we now have **spine resolution** and we report time-lapse movies (**movie 11**) of mRNA particles that are temporarily being recruited by activated dendritic spines. **Overall, our results reveal new mechanisms for how 3'-UTRs can control trafficking: (i) by creating a transport bias in directionality and making it activity-dependent; and (ii) by achieving specific recruitment to *active* synapses.**

3-Staufen2 is a key regulator of mRNA directionality and synaptic anchoring

Finally, as suggested by the reviewer, we completed our previous observations by investigating whether the recruitment of mRNAs to synapses was indeed Staufen2-dependent. Here, we would like to remind the reviewers that Staufen2 is actually critically required for dendritic spine morphogenesis and synaptic function (Ref. #27, Goetze & Kiebler, 2006, *JCB;* Ref #33, *Berger et al., 2017, Genome Biol.*). This important finding clearly indicated that Stau2 plays an important role at mature synapses. However, it did not reveal whether this loss of synapses is due to reduced synaptic recruitment of mRNAs. Here, we addressed this question by down-regulating Stau2 in neurons and investigating synaptic docking/undocking of mRNAs to synapses by dual-color live cell microscopy. However, due to the complexity of the experiment (1 viral transduction followed by three plasmid transfections in mature primary neurons) and the experimental limitation of Stau2 affecting synapses in the first place, we were unable to get reliable results in time for this revision. We therefore performed three alternative lines of research to address this important question. First, we now provide dual-color time-lapse videomicroscopy using *MS2-Rgs4* and tagRFPt-Stau2 to show cotransport of Staufen2 and its target mRNA, *Rgs4*, in dendrites (**Fig. 6A**, **movies 12, 13**). Second, when Stau2 was overexpressed in neurons, we observed a significant increase of dendritic mRNA particles, which were dependent on the Rgs4 3'-UTR (**Suppl. Fig. 6A**). Third, we performed a pilot experiment involving MS2 RNA tethering of Stau2 to the reporter mRNA using Stau2-MCP together with *MS2 only* control mRNA. Interestingly, tethering of Stau2 to the control mRNA brought it closer to synapses more frequently (**Suppl. Fig. 6B**). While Stau2 was previously involved in RNA localization in the dendrites of neurons (Ref. #26, Sharangdhar & Kiebler, 2017 *EMBO Rep.*; Ortiz & Gallego 2017 *Cell Rep.*), the mechanisms were not characterized and **our results now demonstrate a role of Stau2 in two key aspects of RNA trafficking: enforcing a directionality bias and synaptic anchoring.**

Point-by Point answers to the Reviewers' comments:

Reviewer #1 (Remarks to the Author):

The manuscript by Bauer et al. describes the 3′UTR-dependent transport of Rgs4 mRNA in live neurons. Using the MS2-GFP system, the authors studied the role of the 3′UTR of Rgs4 in the biased transport of RNA granules in neurons. They found that bidirectional transport of RNA in dendrites was independent of the presence of the Rgs4 3′UTR. However, the Rgs4 3′UTR caused (1) a biased transport to the anterograde direction and (2) sustainable recruitment to the synapses. Either shRNA knock-down of Staufen2 or chemical inhibition of synaptic activity abolished these effects of Rgs4 3′UTR.

The study is well-conducted to determine the role of the 3′UTR of Rgs4 mRNA in RNA trafficking in dendrites. However, the novelty of their finding is not very clear from the manuscript. The MS2-GFP system has been used to investigate the trafficking of various reporter mRNAs in neurons (some of the references are listed in my comment #1). Most of the results in the manuscript seem to be consistent with the previous findings in the literature on mRNA transport, although this is the first time to investigate Rgs4 mRNA trafficking.

Thank you for your positive assessment of our work. We agree with this referee that the novelty has not become clear to the reader and we spent a significant effort to improve this part (see our detailed response, which addresses comments from all three referees to a certain extent, at the beginning of this file).

Nonetheless, the authors performed a thorough analysis on the role of Staufen2 and neuronal activity for the trafficking of Rgs4 mRNA. The analysis schemes used in this study will be useful for the community to compare the effect of 3′UTR or other cis-regulatory elements in many dendritically localized mRNAs. To strengthen the manuscript, I suggest that the authors address a few points described below.

1. In the discussion section, the authors need to compare their results with previous reports on other 3′UTRs labeled with the MS2 system. In addition to the three references (33, 34, 37), there are several more studies using the MS2-labeled mRNA. Here are some examples:

- Rook et al., J. Neurosci., 2000: *CaMKIIalpha 3' untranslated region-directed mRNA translocation in living neurons: visualization by GFP linkage*
- Dynes et al., J. Comp. Neurol., 2007: *Dynamics of Bidirectional Transport of Arc mRNA in Neuronal Dendrites*
- Kao et al., PNAS, 2010: *Altered mRNA transport, docking, and protein translation in neurons lacking fragile X mental retardation protein FMRP and CaMKIIalpha RNA*
- Dictenberg et al., Dev. Cell, 2008: *A Direct Role for FMRP in Activity-Dependent Dendritic mRNA Transport Links Filopodial-Spine Morphogenesis to Fragile X Syndrome*
- Meer et al., PNAS, 2012: *Identification of a cis-acting element that localizes mRNA to synapses.*

Of course, we agree with this referee and added all indicated references from this as well as those from the other referees.

2. Please provide some discussion on the possible molecular mechanism of the Staufen2-mediated biased mRNA transport. Also, more discussion on the known function of Staufen2 in neurons would be informative. For instance, these papers seem to be highly relevant to this study.

- Lebeau, Staufen 2 regulates mGluR LTD and Map1b mRNA distribution in hippocampal neurons. Learn. & Memory, 2011
- Goetze et al., The brain-specific dsRBD Staufen2 is required for dendritic spine morphogenesis. J Cell Biol, 2006

We agree with this referee and discussed these key papers, which we had cited, in this context in the discussion.

3. In page 16 line 369: there is another recently published computational study related to the sushi-model:

- Song et al., Neuronal Messenger Ribonucleoprotein Transport Follows an Aging Lévy Walk. Nature communications, 2018

Thank you for pointing out this interesting publication. We have included it in the paragraph suggested. This very interesting study addressed the run distribution of movements more specifically to test whether it would fit a \`Levy walk´, which could lead to optimized target searches. Note that the experimental data are derived from 1 minute long single-color movies and do not address two important properties of the sushi-belt model (that we measure in our study): bi-directional movements of single particles and transient stops at synapses.

4. In Figure 2D,F,H, Figure 3B,D,F, Figure 4B,G, Figure 5A,C, and other similar figures in the supplementary data, there seem to be only 3 data points, which was alarming to me at first. Only after a careful reading of the figure legends, I understand that these are 3 independent experimental data. To avoid such a misunderstanding, I suggest the authors change these figures as simple bar graphs. Also, it would be informative to provide the number of the mRNAs counted.

Thank you for sharing your concern with us. We agree that we should be more clear on this important point and changed the text accordingly. To visualize where the individual data points lie more accurately, we chose – and still prefer – to show this type of representation (dots plots) as also the scientific field (and most journals) have a strong tendency to correctly represent the underlying single experiments of the means provided. However, we leave this at the final discretion of the reviewer and the editor.

5. Figure 1C, D, and E are better to be moved to the supplementary information. The texts “speed”, “displacement”, and “directionality” are not necessary in these figure panels.

We appreciate the comment of the reviewer. We have deleted the indicated text parts from the figure. To be able to compare the upcoming figures more directly, we prefer to keep the three panels in the main figure, as they directly relate to the graphs in Figs. 1F, 2 and Suppl. Figs. S4 and S6. However, we leave this at the final discretion of the reviewer and the editor.

Minor comments:

6. The length of the 3′UTRs in Rgs4 and histone-3.3 mRNAs need to be provided in the manuscript.

Thank you for comment, we have now included this information in the Methods section of the revised manuscript.

7. In page 9, what is F_2,9? It needs to be defined in the manuscript.

Thank you for pointing this out to us, we have now included this information in the Methods section of the revised manuscript.

8. Supplementary Figure 1B seems to be a control experiment with GFP rather than tdMCP-GFP because no puncta are visible in the middle panel. This needs to be explained in the figure legend.

We agree and have now improved the figure legend of **Suppl. Figure 1B**. It now says: “(B) Phase contrast, GFP fluorescence and MS2 single molecule FISH of rat hippocampal neurons co-expressing control GFP (***not fused to MCP***) and the *Rgs4* MS2 reporter mRNA (schematic representation top right)”.

9. The movies need to show the unit of time.

Thank you for this suggestion. We have modified the figure legends of the movies (Supplementary information) and the movies themselves. It now includes “**s**” for seconds after the time display.

10. 3’UTR needs to be corrected to 3′(prime)UTR.

Thank you for bringing this to our attention. This has been corrected throughout as suggested.

Reviewer #2 (Remarks to the Author):

The paper provides insights into mRNA sorting mechanisms in dendrites of rodent hippocampal neurons. Dynamics of Rgs4 mRNA movement and synaptic docking are analyzed in detail using MS2-based RNA tagging and high spatiotemporal imaging. There is a small, statistically significant anterograde bias to mRNA transport when the Rgs4 3UTR and Stau2 protein are present. This bias, as well as 3'UTR stimulated docking of RNA near synapses, is promoted by neuronal activity.

General evaluation:

This is a high quality study and I have no substantial scientific issues with the presented data or associated interpretations. However, the overall extent to which the data advance our understanding of neuronal mRNA trafficking is diminished by previously published work on the dynamics of mRNAs in hippocampal neurons. Reference 11 in the manuscript describes detailed evaluation of beta-actin mRNA docking and translation at synapses using RNA tagging and neuronal stimulation, and also includes some analysis of mRNA transport (although does not address directionality). Furthermore, reference 37, as well as two papers by Dynes and Steward (J Comp Neurol. 2007 Jan 20;500(3):433-47 and J Comp Neurol. 2012 Oct 1;520(14):3105-19) demonstrate that there is an anterograde bias in the transport of tagged Arc mRNAs, though this is not activity dependent. Despite this, the work by Bauer et al. is a significant advance as the 3'UTR dependence of mRNA dynamics is revealed, Stau2 is identified as a regulator of directionality, and it is shown that hippocampal neurons employ activity-dependent and -independent mRNA sorting mechanisms. Nonetheless, some of the key conclusions overlap with those in published studies (anterograde bias of dendrite localized RNAs and RNA docking at synapses stimulated by activity).

Thank you for your positive assessment of our work. We agree with this referee that the original manuscript should have done a better job in implementing the cited work and discussing in detail, what are – at least in our opinion – the novel observation. We spent now a significant effort to improve this part (see our detailed response, which addresses comments from all three referees to a certain extent, at the beginning of this response (page 1-2).

Specific criticisms:

1. The authors truncate the y-axis in many figure panels, which risks misleading readers on the magnitude of effects. Full y-axes should be shown throughout.

Thanks for the suggestion, this has been done as suggested.

2. The sections on the analysis of RNA docking and undocking on pages 11 and 12 are hard to follow. For example, the following is not clear: "In general, synapses contained RNA both at the beginning and during the experiment regardless of their location and the type of the reporter mRNA (Supplementary Fig. 4K-M), indicating that there has to be a net influx of RNA into PSD-95 positive structures". A simplification of the sections on pages 11 and 12, or more intermediate interpretations/conclusions, is needed to clarify this important part of the paper.

Thank you for bringing this to our attention. We agree and have therefore simplified the text for the indicated text passage as suggested. Importantly, we have revised the data processing algorithm to assess the effects of photobleaching correction (**Supplementary Fig. 4N-O**). During this revision, we noticed that when calculating the net flux of the reporter RNA into or out of synapses, we considered only the vectorial sum of arrivals and departures – which was negative for both datasets – but not the actual amount of RNA that arrives or leaves with a single event. Now, we included the corrected figure panel (**Fig. 4F**), which shows that in the case of *Rgs4* 3´-UTR containing reporter RNA we observe a net positive (significantly larger than zero) flux into synapses. In contrast, such net positive influx of the *MS2 only* mRNA is not detected with the current, large dataset (N=942). We thank the reviewer for making us revise this part of the manuscript.

Why is the number of RNA-associated synapses lower with MS2+Rsg4 3'UTR than MS2 alone given there is an anterograde bias to transport and increased docking rate induced by the 3'UTR?

We hypothesize that the observed anterograde transport bias contributes to the synaptic recruitment of mRNA. As both the transport bias and synaptic recruitment are dependent on the 3'-UTR and neuronal activity, the anterograde transport might indeed facilitate synaptic recruitment, especially under endogenous mRNA expression levels. However, this is not immediately obvious from our data.

Our data shows that *MS2+Rgs4* mRNA is indeed present at fewer synapses that *MS2 only* mRNA. However, we also observe a stronger net influx of MS2+Rgs4 at synapses. One possibility to explain this difference could be that processes taking place at much longer time scales erase the expected higher accumulation of *MS2+Rgs4* mRNA at synapses. Another possibility, which we favor, is that the *Rgs4* 3´-UTR mediates defined localization to specific synapses, dependent on neuronal activity. This idea is supported by recent experiments involving glutamate uncaging performed during the revision process and now included in the manuscript (**Fig. 5**, **Suppl. Fig. 5**, and **movie 11, Fig. R5A-C, for the reviewers only**). Here, local glutamate uncaging recruited *MS2+Rgs4* mRNA, but not *MS2 only* mRNA. Therefore, we propose that the recruitment of *MS2 only* mRNA to synapses might represent a state of 'non-specific', default localization, while the *Rgs4* 3'-UTR mediates regulated localization to a defined subset of synapses.

Is RNA abundance throughout the cell similar for these two mRNAs? If not, it may be appropriate to emphasize normalized data (e.g. rate of RNA appearances/disappearance at synapses).

Concerning the levels of expression of the reporter constructs: throughout our study, we have analyzed cells with moderate levels of expression of the reporters. Cells with elevated levels of particles were excluded of the analysis to avoid artifacts due to the high levels of expression *per se*. Moreover, the levels of expression and the average number of particles per dendrite were similar in both MS2-control and *Rgs4* 3'UTR, under basal conditions or treated with DMSO or the silencing mix (**Figure R1**; ***for reviewers only***).

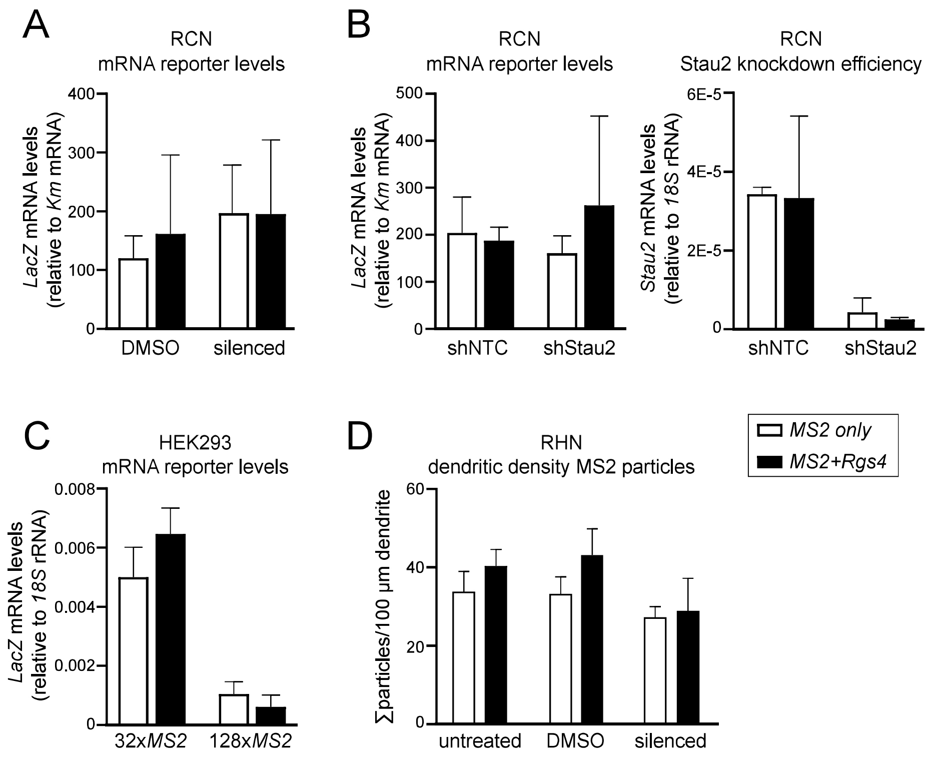

Figure R1: Evaluation of the mRNA reporter levels. **(A)** Rat cortical neurons (RCN) were transfected at 14DIV with either the *MS2 only* or the *MS2+Rgs4* reporter and the *LacZ* mRNA reporter levels were evaluated by RT-qPCR at 15 DIV, after vehicle (DMSO) or silencing (100µM CNQX, 50µM AP5, 1µM TTX) treatment (n=3 experiments; mean+SEM). **(B)** RCN were transduced at 10 DIV with lentiviral particles coding for either short hairpin non-targeting control (shNTC) or Staufen2 (shStau2). Cells were transfected at 14DIV with either the *MS2 only* or the *MS2+Rgs4* reporter and *LacZ* mRNA reporter levels (left panel) or *Stau2* mRNA levels were evaluated by RT-qPCR at 15 DIV (n=3 experiments; mean+SEM). **(C)** HEK293 cells were transfected with either the *MS2 only* or the *MS2+Rgs4* reporter containing either 32xMS2 or 128xMS2 repeats. The *LacZ* mRNA reporter levels were evaluated by RT-qPCR after one day of expression (n=2 experiments; mean+SEM). **(D)** Rat hippocampal neurons (RHN) were co-transfected with the constructs expressing RFP together with MCP-GFP and either the *MS2 only* or the *MS2+Rgs4* reporter. The following day, cells were either untreated, treated with vehicle (DMSO) or silenced (100µM CNQX, 50µM AP5, 1µM TTX) for 1h. Cells were fixed and the RNA granule density in dendrites was evaluated (n=2 experiments, ≥20 dendrites/condition; mean+SEM).

In addition, we have performed further control experiments to evaluate the levels of expression of the different reporters that we have used in our study. Either 293 cells or rat cortical neurons (RCN) were transfected with the reporters and expression was allowed for 24h. Cell lysates were collected and total RNA was purified. Quantitative RT-PCR for the LacZ sequence (ORF included in the reporter) revealed that the *MS2 only* control reporters are expressed at similar levels to the *MS2+Rgs4* reporters (**Figure R1A-C**; ***for reviewers only***). This was the case for both 32xMS2 and 128xMS2 mRNA reporters (**Figure R1C**). In agreement with these findings we found no difference in mRNA reporter granules number in distal dendrites (**FigureR1D**).

3. The authors discuss the possibility of photobleaching of the mRNA. Unless the contribution of photobleaching can be quantified, it would be more appropriate to refer to appearance and disappearance of RNA signal at synapses rather than docking and undocking.

We have extensively analyzed photobleaching during our time-lapse movies. Briefly, we measured the RNA signal under the synapse masks and took the mean for every frame per experiment and the average per reporter (**Suppl. Figure 4N**). For simplicity, we only show every 100th data-point. We observed that there was about 10-fold intensity difference between the dimmest and the brightest experiment and there was an ~ 25% drop in the signal intensity by the end of the experiments.

We normalize this raw RNA signal by the threshold that we have previously defined to represent the intensity of a single unit of the RNA (**Suppl. Figure 4O**). This threshold was calculated for every 100 frame package (*i.e.* for 1-100, 101-200, etc.). More importantly, however, this graph indicates that the drop of signal intensity by photobleaching was corrected in the analysis suggesting that the variations were due to the arrival or departure of MS2+ particles to the synapses.

4. The relationship between net anterograde transport and increased RNA docking at synapses is not clear. At the very least, more speculation on this subject should be included.

Thank you for the suggestion. We agree and added a few sentences on this issue in the new discussion section. In short, we hypothesize that the observed anterograde transport bias contributes to the synaptic recruitment of mRNA. As both the transport bias and synaptic recruitment are modulated by the 3´-UTR and neuronal activity, anterograde transport might indeed facilitate synaptic recruitment, especially under endogenous mRNA expression levels.

5. Although likely a large amount of work, the paper would be stronger if the role of Stau2 in RNA docking was addressed. These experiments may also indicate if the anterograde transport and RNA docking processes can be uncoupled.

We agree with this referee and have performed the requested experiments (see our detailed response at page 2 of this file above). Please be reminded that **Staufen2 is actually critically required for dendritic spine morphogenesis and synaptic function** (Ref. #27, Goetze & Kiebler, 2006, *JCB;* Ref. #33, *Berger et al., 2017, Genome Biol.*). Nevertheless, we performed the obvious experiment down-regulating Stau2 in neurons and investigating synaptic docking/undocking of mRNAs to synapses by dual-color live cell microscopy. As this involves 1 viral transduction followed by three plasmid transfections in mature primary neurons), we were unable to get reliable results for this revision.

We therefore performed **three alternative lines of research** to find out whether Stau2 might indeed be involved in RNA docking. First, Staufen2 and its target mRNA, *Rgs4*, are co-transported the same RNA granules in dendrites (**Fig. 6A**, see new **movies 12, 13**). Second, Stau2 overexpression increases the number of dendritic mRNA particles, which were dependent on the 3'-UTR (**Suppl. Fig. 6A**). Third, a pilot experiment tethering Stau2 to the reporter mRNA tends to recruit the mRNA closer to synapses (**Suppl. Fig. 6B, Fig. R2**). Together, these experiments provide a good support for a direct role of Stau2 in regulating RNA trafficking.

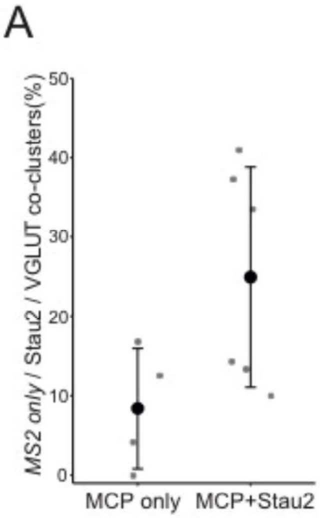

Figure R2 (= Suppl. Figure 6B): Effect of Stau2 on mRNA particle recruitment to synapses. **(A)** Rat hippocampal neurons (RHN) were co-transfected with the constructs expressing *MS2 only*, MCP-GFP and either "MCP only" or "MCP-Stau2". The following day, cells were fixed, and immunostained for Stau2 and the presynaptic marker VGLUT. The percentage of MS2−MCP-GFP + Stau2 + VGLUT dendritic co-clusters, over total MS2−MCP-GFP clusters was quantified. Data represents mean ± standard deviation (individual dendrites shown as gray dots). Data was obtained from 40 µm dendritic segments at a minimal distance of 20 µm from the cell body.

6. The authors should state explicitly in the text whether larger or smaller synapses of brighter or dimmer PSD-95 signal intensity correlate with RNA positive and negative synapses.

Thank you for the suggestion. We agree and the text now clearly states "that mRNA positive synapses were both larger and brighter in their mean PSD-95 signal intensity than mRNA negative synapses" (see **Suppl. Fig. 4H**).

Reviewer #3 (Remarks to the Author):

In this study, Michael Kiebler's group investigates transport dynamics of mRNAs in dendrites of cultured pyramidal neurons, using live imaging of exogenous mRNAs which are indirectly tagged using the MS2 system. The authors primarily compare the dynamics of two tagged RNAs, a control that lacks a 3'UTR (or ones that include the 3'UTR of transcripts that do not localize to dendrites) and a transcript that includes the 3'UTR of Rgs4, known to localize to neuronal dendrites. Using this paradigm, the authors discover that Rgs4 3'UTR mRNAs show a bias in anterograde transport. This phenomenon depends upon neuronal activity, as pharmacological silencing of cultured neurons abolishes this bias. Next the authors focus on mRNA docking at synapses using fluorescently tagged PSD95 together with MS2 live imaging of mRNAs. With this method, they observe that Rgs4 3'UTR mRNAs have a higher probability of docking close to synapses. Taking advantage of endogenous activity of cultured neurons, they show this docking mechanism is mediated by neuronal activity. Finally, the authors employ shRNAs targeting Stau2, which encodes an RNA-binding protein known to bind the Rgs4 3' UTR, to discover that the anterograde bias described above relies on Staufen.

This study is well written and relies on high-quality data. It provides valuable insights into the biological mechanisms by which mRNA transport to dendrites may be coordinated with neuronal activity, and how it could impact activity-related synapse remodeling. With additional controls and some additional experiments, this study will be of impact for the field of mRNA localization in neurons.

Thank you for your positive assessment of our work.

1) While the activity- and STAU2-dependent bias towards anterograde movement is a fascinating finding, the significance of this finding for mRNA localization at synapses is a bit unclear. According to the "sushi-belt" hypothesis, the availability of mRNAs close to synapses would be improved without this bias. As noted by the authors, Yoon et al have demonstrated using β-actin reporters that synaptic activity induces transport, translation, and docking close to synapses (Yoon et al, PNAS, 2016). In light of this study, the authors should better clarify the significance and novelty of their findings.

We agree with this referee that the novelty has not become clear to the reader. We therefore spent a significant effort to improve this part (see our detailed response, which addresses comments from all three referees to a certain extent, at the beginning of this file). We also modified the main text, particularly

the introduction (now referencing explicitly our sushi model) and the discussion accordingly. Briefly, we believe that our novelty is to: (i) provide an exhaustive demonstration of the sushi-belt model, with experimental verification of two of its key properties (bi-directional movements of single mRNAs, transient pausing at synapses); (ii) a role of the 3'-UTR in enforcing an activity-dependent directionality bias, as well as a specific association at activated synapses; (iii) a direct role of Staufen2 in promoting the directionality bias and synaptic association.

2) The main conclusions of this study rely on overexpression of 2 MS2-tagged constructs. However, a major caveat to their interpretations is the levels of expression of these exogenous constructs, Rgs4 3' UTR v MS2 control, which could be vary due to differential transcription or stability. This caveat must be addressed.

We appreciate the concern of this referee (as well as Ref. #2). Please see our detailed response for Ref. #2, main criticism 2, lower part, including **Fig. R1** (for the reviewers only) and also **Fig. R3A** (see below)

Perhaps one approach would be to introduce mutation(s) into the 3'UTR for binding sites for Staufen, and compare these to the full length 3' UTR. The authors previously identified Stau2 binding sites on Rgs4. This would also link the 3'UTR analyses to Staufen 2 knockdown, which itself could be indirect.

We have previously reported that the *Rgs4* mRNA 3´-UTR (ENSRNOG0000002773) contains two Type III Staufen recognition sites, termed SRSs (Heraud-Farlow, 2013). Moreover, endogenous Rgs4 and Rgs4 reporter expression significantly decreases upon Stau2 downregulation, suggesting that Stau2 stabilizes *Rgs4* mRNA via its 3´-UTR (Heraud-Farlow, 2013). Following your suggestion, we have generated several deletion mutants of the Rgs4 3'UTR-luciferase reporter and tested whether Stau2 expression regulated the expression levels of the respective reporter. However, we did not find differences in expression between wt *Rgs4* 3´-UTR-luciferase reporter and the mutant versions (**Figure R3, for reviewers only**), suggesting that these (local) SRSs are not solely responsible for mediating the binding of Stau2 to Rgs4 3´-UTR. We are currently favoring the working hypothesis that mammalian Staufen proteins might bind to long-range RNA duplexes instead of short SRSs as originally anticipated (see the paper by our collaborator on that: Sugimoto & Ule, 2015, *Nature*). Consequently, we are currently performing hiCLIP from brain with the Ule lab to identify Stau2 binding sites. However, this is beyond the scope of this manuscript.

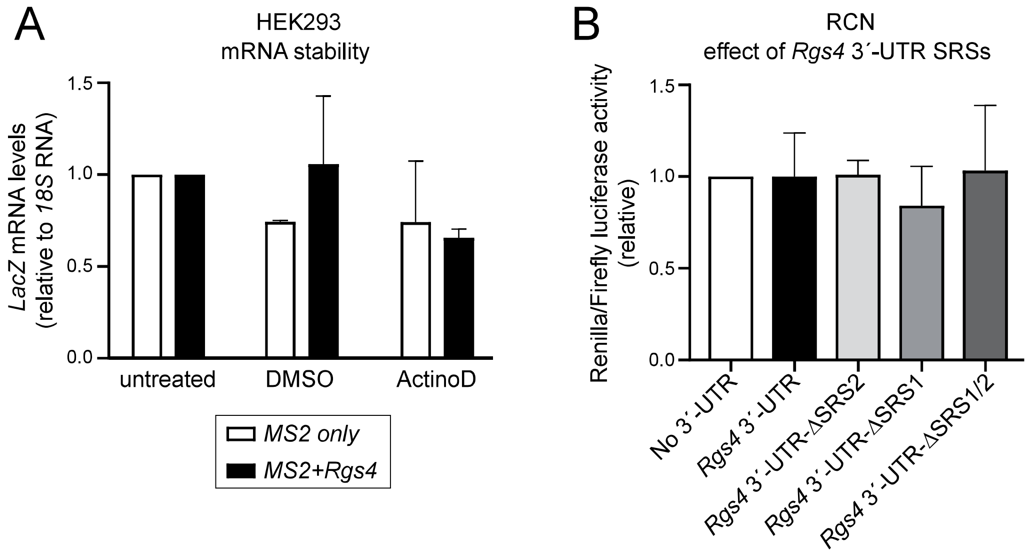

Figure R3: Analysis of mRNA stability and effect of the *Rgs4* 3´-UTR SRSs on translation. (**A**) HEK293 cells were transfected with either the *MS2 only* or the *MS2+Rgs4* reporter. The following day, cells were either untreated, treated with vehicle (DMSO) or actinomycin D for 3-4h. Then, *LacZ* mRNA reporter levels were evaluated by RT-qPCR (n=2 experiments; mean+SEM). (**B**) Quantification of luciferase activity expressed at 1DIV in rat cortical neurons (RCN) that were nucleofected on the day of isolation with a dual plasmid co-

expressing control *Firefly* and *Renilla* luciferase containing either no 3´-UTR, wt *Rgs4* 3´-UTR or mutated *Rgs4* 3´-UTR lacking SRS2 and/or SRS1 (as described in Heraud-Farlow, 2013) (n≥4 experiments; mean+SEM).

3) Related to this, to visualize mRNAs transport in dendrites, the authors utilize a version of the MS2 system which relies on the use of tdMCP-EGFP that includes a nuclear localization sequence (NLS) to decrease background noise in the cytoplasm. NLS sequences have been shown to promote microtubule binding and transport of proteins (Salman et al, Biophysical Journal, 2005). The presence of the NLS could thus potentially lead to non-physiological results. This may be especially true given the high number of MS2 loops that the authors used in their constructs. To address this concern, the authors could employ a different mRNA-tagging paradigm that relies on MS2/PP7 split-FPs. The authors wouldn't need to redo all experiments but just provide evidence that some aspects of mRNA transport are phenocopied (anterograde movement bias OR preferential docking close to synapses).

- Salman H et al., Biophys J. 2005 Sep;89(3):2134-45. *Nuclear localization signal peptides induce molecular delivery along microtubules.*

We appreciate this important concern of this referee. We performed the following experiment and wrote this sentence in the results section: "As an NLS might potentially affect transport as previously reported (Salman 2005), we generated a tdMCP lacking the NLS and repeated the previous experiment. Importantly, we still observed an anterograde transport bias mediated by the Rgs4 3´-UTR, showing that the NLS did not affect trafficking in our hands" (**Fig. R4A, for reviewers only**).

In an initial experiment investigating the differences in MS2+Rgs4 mRNA reporter transport either with (+NLS) or without (-NLS) an NLS, we were able to reproduce the Rgs4 3'-UTR dependent anterograde transport bias independent of an NLS and at two different transfection ratios (**Fig. R4B**). Moreover, the MS2 only reporter mRNA showed no anterograde bias compared to MS2+Rgs4, in the absence of an NLS (**Fig. R4C** and **Suppl. Fig. 2D**). This is in line with experiments including an NLS (**Fig. R4D** and **Fig. 2D**), demonstrating that use of an NLS in our system does not affect our observations and is indeed a valid tool to reduce fluorescent background in the MS2 system.

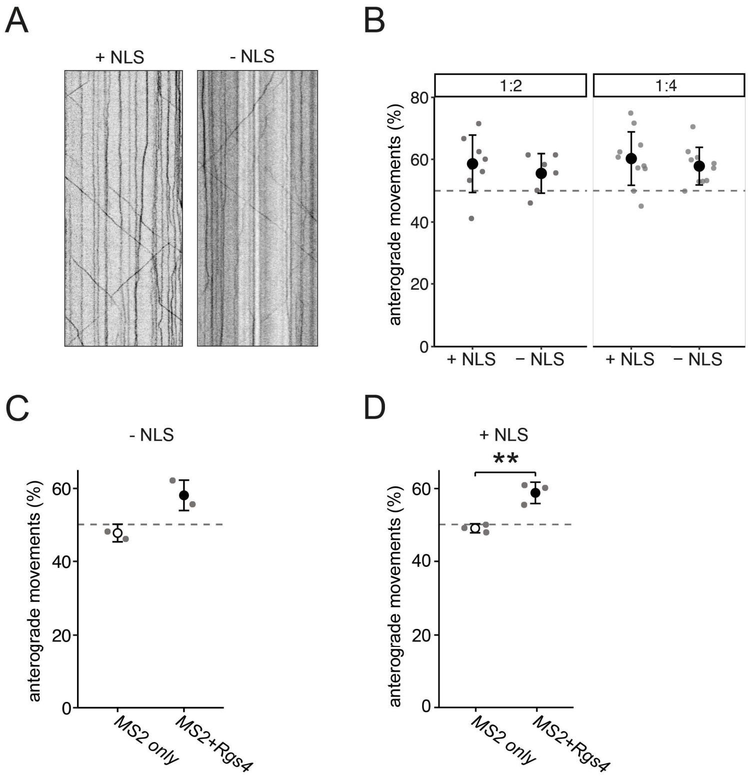

Figure R4: Effect of the NLS on tracking mRNA particle movement. (**A**) Representative kymographs of GFP fluorescence of hippocampal neurons co-transfected with the *MS2+Rgs4* 3´-UTR reporter and tdMCP-GFP constructs either with (+NLS) or without (-NLS) a nuclear localization signal (NLS) **(= Suppl. Fig. 2C**). (**B**) Dot plot displaying percentage of anterograde moving granules for *MS2+Rgs4* 3´-UTR reporter mRNA, detected by either tdMCP-GFP (-NLS) or NLS-tdMCP-GFP (+NLS). The indicated plasmids were transfected at a ratio 1:2 or 1:4 (MCP:reporter). (**C**,**D**) Dot plot displaying percentage of anterograde moving granules for *MS2 only* or *MS2+Rgs4* 3´-UTR reporter mRNAs, detected by either tdMCP-GFP (-NLS) or NLS- tdMCP-GFP (+NLS). **C** = Suppl. Fig. 2D, **D** = Fig. 2D). (**B**,**C**,**D**) Data represents mean ± standard deviation of independent experiments (mean of each experiment is shown as gray dot). Data was obtained from 40 µm dendritic segments at a minimal distance of 20 µm from the cell body. Only displacements ≥ 1.5 µm were considered for analysis.

4) The authors show that neuronal silencing affects anterograde movement and docking, and use endogenous activity in cultures and washout studies to implicate activity in promoting the movement. This point would be better supported if the authors were to stimulate activity and show how responsive (temporally and also qualitatively) the RNAs are to targeting? (For example, using glutamate uncaging of hippocampal neurons, as recently used to induce RNA localization to synapses by Yoon et al, 2016).

We appreciate this important concern of this referee. We have now performed glutamate uncaging ourselves and followed the fate of the visualized RNA granules in real-time. Interestingly, we observed **an average increase of ~ 3 RNA granules** for the *MS2+Rgs4* reporter mRNA, while there was no additional increase for *MS2 only* reporter (**new Fig. 5A-B**, **Suppl. Fig. 5B**, **movie 11, Fig R5A-C**). In conclusion, this provides strong additional experimental evidence that it is the Rgs4 3´-UTR that has a direct influence on the activity-dependent mRNA docking/undocking, and that the docking more specifically occurs on activated synapses.

Further, with the current data, there is incomplete rescue of anterograde movements in Figure 3. If they waited longer to do analysis following washout, would the authors see more complete recovery or do they speculate some cells are not responsive?

We thank you for pointing this out. First, we investigate if we saw an increased recovery in our existing data set during the imaging time (**Fig.R5D**), however this was not the case. Therefore we decided to allow recovery for 6h before imaging. Though we were able to reproduce our original data set we did not see additional recovery (**Fig. R5E**). Importantly, though we did not gain full recovery, there was no statistical difference between vehicle treatment and recovery.

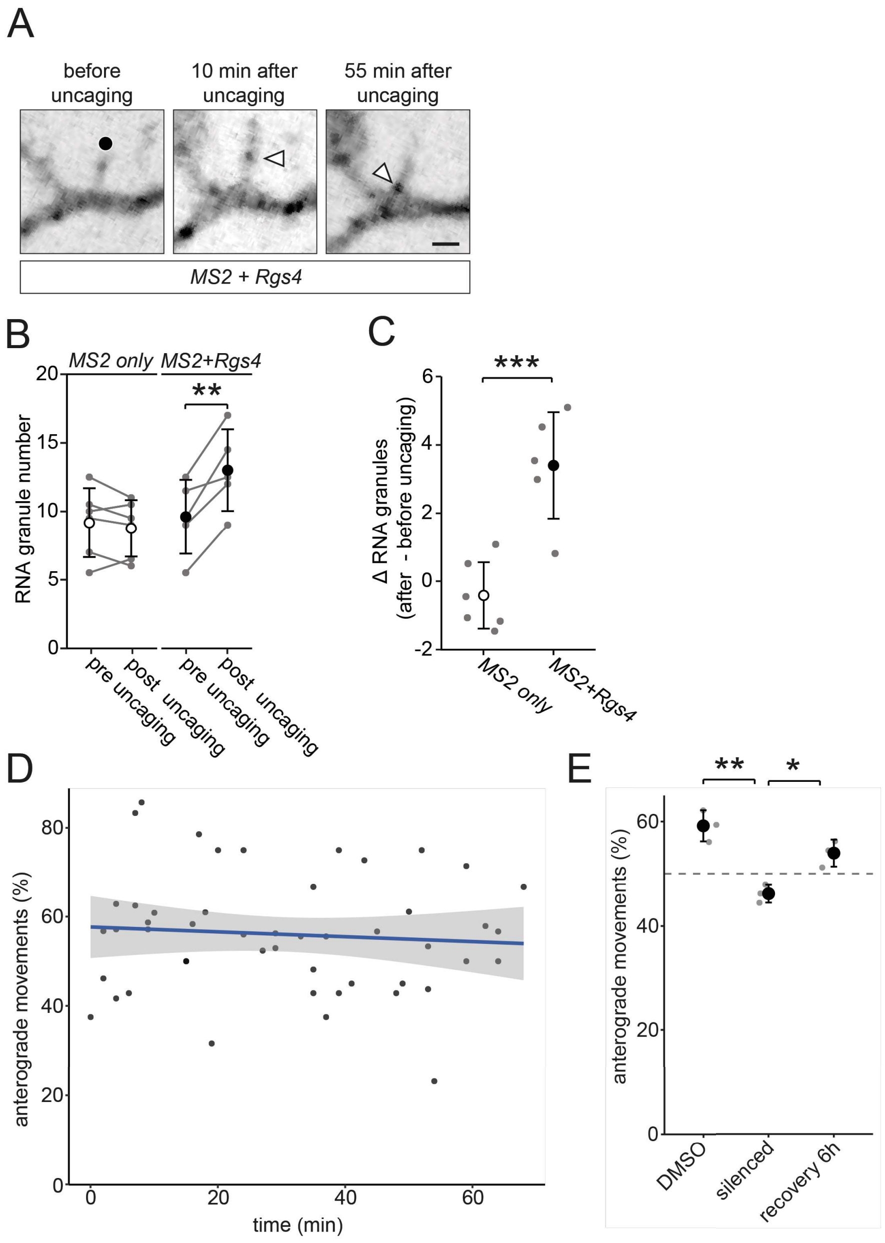

Figure R5: Effect of synaptic stimulation on mRNA recruitment. Local glutamate uncaging at individual dendritic spines triggers *Rgs4* 3´-UTR dependent mRNA recruitment. (**A, = Fig. 5A**) Representative GFP fluorescence of a hippocampal neuron co-transfected with the *MS2+Rgs4* 3´-UTR reporter and tdMCP-GFP constructs before (left panel) and after (middle, right panels) glutamate uncaging. Arrowheads indicate GFP positive MS2 reporter mRNA granules. Scale bar = 2 µm. (**B, = Suppl. Fig. 5B**) Dot plot displaying the number of RNA granules of *MS2 only* or *MS2+Rgs4* 3´-UTR reporter mRNAs pre (7-2 min before) and post (40-45 min after) uncaging in hippocampal neurons within 5 µm of the stimulated spine. (**C, = Fig. 5B**) Dot plot displaying the change in RNA granule number 40-45 min after uncaging compared to the RNA granule number 2-7 min before uncaging within 5 µm of the stimulated spine. Data represents mean ± standard deviation (individual neurons shown as gray dots). Asterisks represent *p*-values (****p* < 0.001, ***p* < 0.01). Data was obtained from 6 dendrites (5 neurons of 4 biological replicates) and 5 dendrites (5 neurons of 5 biological replicates) for *MS2 only* and *MS2+Rgs4* reporter mRNAs, respectively. (**D**) Dot plot and linear regression displaying percentage of anterograde moving granules for *MS2+Rgs4* 3´-UTR reporter mRNAs in neurons that were silenced (100 µM CNQX, 50 µM AP5, 1 µM TTX, 1 h), and then recovered for 1 h before imaging. Dots represent individual cells. (**E**) Dot plot displaying percentage of anterograde moving granules of *MS2+Rgs4* 3´-UTR reporter mRNAs in neurons, either vehicle-treated (DMSO) or silenced (100µM CNQX, 50µM AP5, 1µM TTX) and upon 6h recovery. Data represents mean ± standard deviation of 3 independent experiments (mean of each experiment is shown as gray dot). Asterisks represent *p*-values assessed by Tukey's test post-hoc to one-way ANOVA analysis (**p* < 0.05, ***p* < 0.01). Data was obtained from 40 µm dendritic segments at a minimal distance of 20 µm from the cell body. At least 10 dendrites/condition/experiment were analyzed.

Minor concerns

1) The authors do not define in the methods or main text how long the 3' UTR is for Rgs4.

Thank you for pointing this out to us, we have now included this information in the Methods section of the revised manuscript.

2) Please define sushi-belt in introduction, when first mentioning it. Alternatively, please remove.

Thank you for this suggestion, of course we are happy to include this. We have therefore modified the introduction accordingly.

3) The authors should show graphs reporting both speed and fraction of RNAs moving, when activity is blocked for both MS2 and Rgs4 constructs in Figure 3.

Thanks. We have included the indicated quantifications (**Suppl. Figure 3**G-J).

4) The authors should include a control to show that overexpression of tagged PSD95 mimics endogenous synapses in fixed cells as it could potentially produce ectopic synapses.

Thank you for the suggestion, we have performed the experiment and found that there were no significant changes in synapse number, as vGLUT clusters did not change (**Figure R6**).

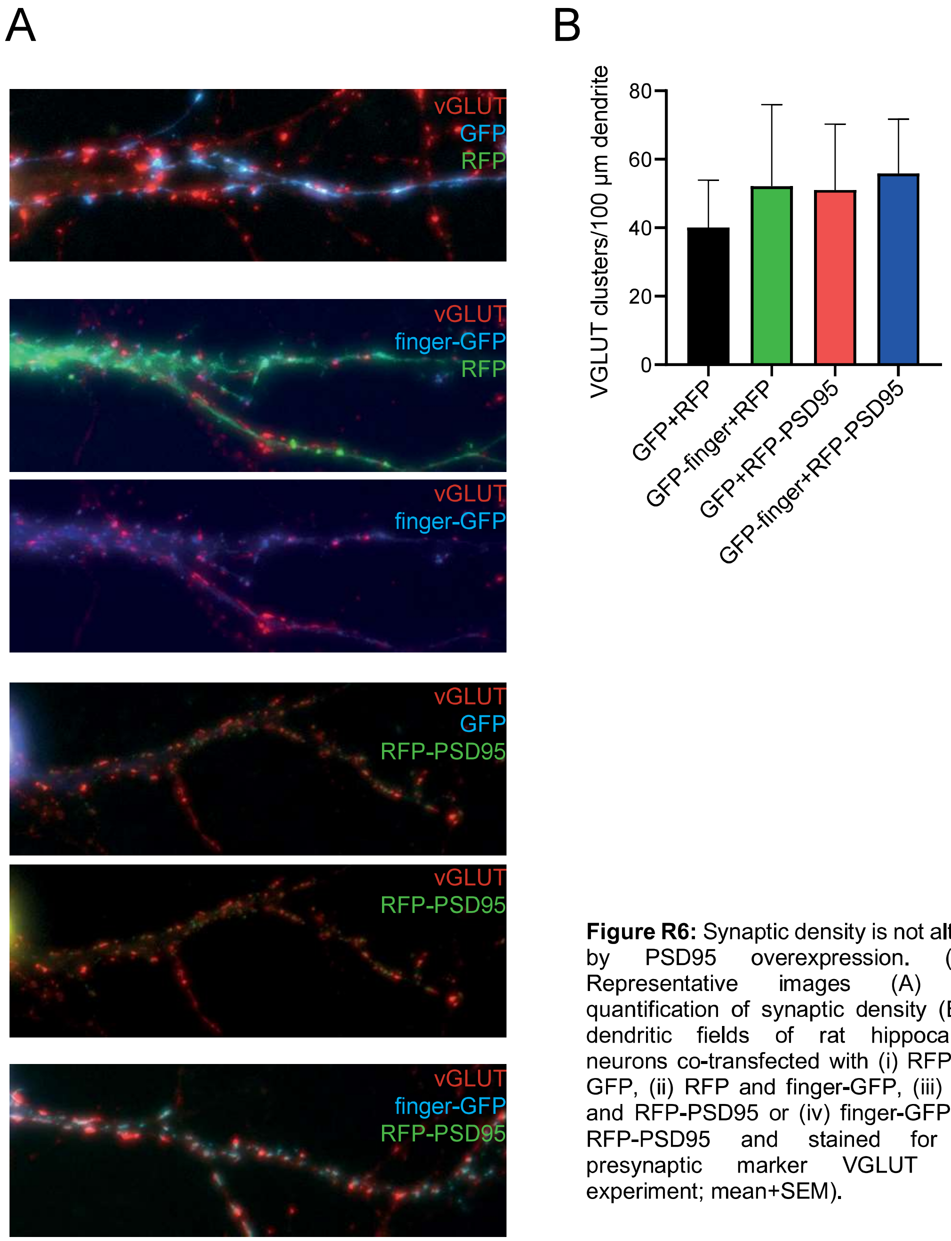

Figure R6: Synaptic density is not altered by PSD95 overexpression. (**A,B**) Representative images (A) and quantification of synaptic density (B) of dendritic fields of rat hippocampal neurons co-transfected with (i) RFP and GFP, (ii) RFP and finger-GFP, (iii) GFP and RFP-PSD95 or (iv) finger-GFP and RFP-PSD95 and stained for the presynaptic marker VGLUT (n=1 experiment; mean+SEM).

5) The role of STAU2 in transport is interesting. Does STAU2 also regulate preferential docking close to synapses? If tagged would STAU2 move with RNAs? It could be valuable to flesh this part of the story out more to establish a direct relationship, as pointed out in point 1 above.

Thank you for pointing this out. As also Ref. #2 asked for the experiment, we have performed a series of experiments that are described in detail (see above, our response to Ref. #2, point 5, Fig. R2, Fig6A, Supplementary Fig.6A-B). But also see the general comment on page 1 of this document.

6) For a better assessment of the quality of the data, the authors should better define the nature of their biological replicates? How many neurons where analyzed for each replicate? How many movements?

We appreciate the suggestion of the reviewer. The values of the number of experiments, neurons and particles are now included for all figures.

7) In two instances, it would be very valuable to merge graphs together and adjust statistical analyses

accordingly. This would provide a more powerful way to interpret results. This should be done for Figure 3: B,D,F and Figure 5:A,C.

We appreciate the suggestion of the reviewer. We have generated the requested figures and show both in the **Fig. R7** (**for the reviewers only**). Statistical analysis of this merged data indeed supports our previous interpretation. As the data points are from different biological replicates, we would strongly opt to show them separate in the manuscript. However, we leave this at the final discretion of the reviewer and the editor.

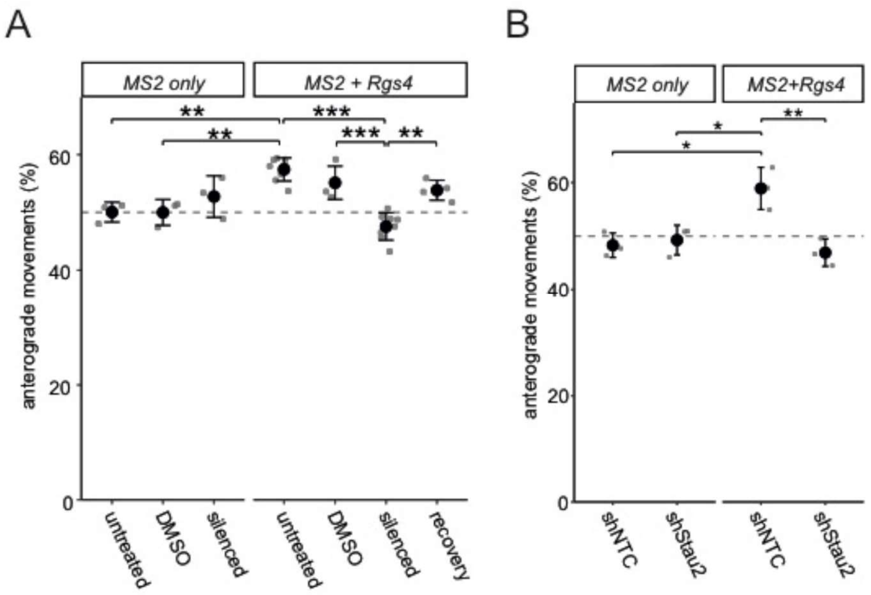

Figure R7: Dot plots combining the values displayed in Figures 3B,D,F (**A**) and 6B,D (**B**). Data represents mean ± standard deviation of 3-8 independent experiments (mean of each experiment is shown as gray dot). Asterisks represent *p*-values assessed by Tukey's test post-hoc to one-way ANOVA analysis (**p* < 0.05, ***p* < 0.01, ****p* < 0.001).

REVIEWERS' COMMENTS:

Reviewer #1 (Remarks to the Author):

The authors have addressed all of my concerns. I am satisfied with the revised manuscript.
I only suggest the authors need to add details of how they classify “RNA=0” and “RNA>=1” synapses. If there are RNA docking and undocking events at a synapse, how can you say RNA=0 at that synapse?

Reviewer #2 (Remarks to the Author):

The authors have done a commendable job of addressing the reviewer's comments. Overall, the findings complement and extend previous work on this topic. I recommend publication of the manuscript but have some suggestions for improving the manuscript.

1. The data in Figure R1 (in the rebuttal letter) are valuable (and address a point raised by two reviewers) and the authors should consider including the key panels in the supplementary figures.

2. Figure 4: the panels are not displayed in an order that is intuitive alphabetically. Can this be corrected?

3.Figure 5A. The black dot that shows the site of uncaging is not immediately distinguishable from the black puncta in the neuron. The authors could use an asterisk to mark the uncaging site, or a colored dot.

4. Figure S4 is huge and should be split into different supplementary figures.

5. Line 291. The statement is weak: “These findings suggest that dendritically localized MS2+Rgs4 mRNA was probably associated with a specific subset of synapses”. The authors could omit the word ‘probably’ as the statement is already qualified by ‘suggest’.

6. Line 385: As this is clearly a model, the word ‘might’ is not necessary

7. Line 336: Why the term ‘pilot’ for this experiment? Multiple data points are included. The authors are cautious with the interpretation of the experiment and the word ‘pilot’ may not be necessary.

Reviewer #3 (Remarks to the Author):

The authors are to be commended for their thorough attention to all concerns raised by reviewers with additional new data and significant clarifications to the text. This paper will make an outstanding addition to the field and provides a nice paradigm for other investigations of RNA localization and translation in synapses and beyond. I have no remaining concerns.

Response to the reviewers

We would like to sincerely thank all three reviewers for the productive review process and the positive comments. Please fin our remarks to the second round of comments below.

Reviewer #1 (Remarks to the Author):

The authors have addressed all of my concerns. I am satisfied with the revised manuscript.
I only suggest the authors need to add details of how they classify “RNA=0” and “RNA>=1” synapses. If there are RNA docking and undocking events at a synapse, how can you say RNA=0 at that synapse?

Thank you for your kind and positive comments.
To clarify that “RNA=0” and “RNA>=1” indicated in the figures refers to the RNA content at the beginning of the acquisition, we modified the figure legends. They now include “(estimated RNA number ≥1 at t=0s)” and “(RNA=0 at t=0s)”.

Reviewer #2 (Remarks to the Author):

The authors have done a commendable job of addressing the reviewer's comments. Overall, the findings complement and extend previous work on this topic. I recommend publication of the manuscript but have some suggestions for improving the manuscript.

Thank you for your positive remarks.

1. The data in Figure R1 (in the rebuttal letter) are valuable (and address a point raised by two reviewers) and the authors should consider including the key panels in the supplementary figures.

We have included all panels where appropriate (new Supplementary Fig. 1, 4 and 10).

2. Figure 4: the panels are not displayed in an order that is intuitive alphabetically. Can this be corrected?

We agree and believe we have found a new more intuitive arrangement (see adapted Fig. 4).

3. Figure 5A. The black dot that shows the site of uncaging is not immediately distinguishable from the black puncta in the neuron. The authors could use an asterisk to mark the uncaging site, or a colored dot.

Thank you for pointing this out. As an asterisk is already used to indicate statistical significance in the same figure we decided to exchange the dot for an arrow.

4. Figure S4 is huge and should be split into different supplementary figures.

Yes, we split up the figure.

5. Line 291. The statement is weak: "These findings suggest that dendritically localized MS2+Rgs4 mRNA was probably associated with a specific subset of synapses". The authors could omit the word 'probably' as the statement is already qualified by 'suggest'.

We agree and edited the line.

6. Line 385: As this is clearly a model, the word 'might' is not necessary

We believe the reviewer possibly intended to point out line **485** here. We adapted this line (that was referring to our model) accordingly.

7. Line 336: Why the term 'pilot' for this experiment? Multiple data points are included. The authors are cautious with the interpretation of the experiment and the word 'pilot' may not be necessary.

We agree and edited the line.

Reviewer #3 (Remarks to the Author):

The authors are to be commended for their thorough attention to all concerns raised by reviewers with additional new data and significant clarifications to the text. This paper will make an outstanding addition to the field and provides a nice paradigm for other investigations of RNA localization and translation in synapses and beyond. I have no remaining concerns.

Thank you for your positive remarks to the review process. We are happy we were able to address the concerns.
